# Epigenomic dysregulation-mediated alterations of key biological pathways and tumor immune evasion are hallmarks of gingivo-buccal oral cancer

**DOI:** 10.1186/s13148-019-0782-2

**Published:** 2019-12-03

**Authors:** Debodipta Das, Sahana Ghosh, Arindam Maitra, Nidhan K. Biswas, Chinmay K. Panda, Bidyut Roy, Rajiv Sarin, Partha P. Majumder

**Affiliations:** 1 National Institute of Biomedical Genomics, P.O.: N.S.S, Kalyani, 741251 India; 2 Chittaranjan National Cancer Institute, Kolkata, India; 3 Indian Statistical Institute, Kolkata, India; 4 Advanced Centre for Treatment Research and Education in Cancer, Mumbai, India

**Keywords:** Gingivo-buccal oral squamous cell carcinoma, OSCC, Epigenomic, Transcriptomic, Integrative analysis, Immunotherapeutic marker

## Abstract

**Background:**

Gingivo-buccal oral squamous cell carcinoma (OSCC-GB) is the most common cancer among men in India and is associated with high mortality. Although OSCC-GB is known to be quite different from tongue cancer in its genomic presentation and its clinical behavior, it is treated identically as tongue cancer. Predictive markers of prognosis and therapy that are specific to OSCC-GB are, therefore, required. Although genomic drivers of OSCC-GB have been identified by whole exome and whole genome sequencing, no epigenome-wide study has been conducted in OSCC-GB; our study has filled this gap, and has discovered and validated epigenomic hallmarks of gingivobuccal oral cancer.

**Methods:**

We have carried out integrative analysis of epigenomic (*n* = 87) and transcriptomic (*n* = 72) profiles of paired tumor-normal tissues collected from OSCC-GB patients from India. Genome-wide DNA methylation assays and RNA-sequencing were performed on high-throughput platforms (Illumina) using a half-sample of randomly selected patients to discover significantly differentially methylated probes (DMPs), which were validated on the remaining half-sample of patients.

**Results:**

About 200 genes showed significant inverse correlation between promoter methylation and expression, of which the most significant genes included genes that act as transcription factors and genes associated with other cancer types. Novel findings of this study include identification of (a) potential immunosuppressive effect in OSCC-GB due to significant promoter hypomethylation driven upregulation of *CD274* and *CD80*, (b) significant dysregulation by epigenetic modification of *DNMT3B* (upregulation) and *TET1* (downregulation); and (c) known drugs that can reverse the direction of dysregulation of gene expression caused by promoter methylation.

**Conclusions:**

In OSCC-GB patients, there are significant alterations in expression of key genes that (a) regulate normal cell division by maintenance of balanced DNA methylation and transcription process, (b) maintain normal physiological signaling (PPAR, B cell receptor) and metabolism (arachidonic acid) pathways, and (c) provide immune protection against antigens, including tumor cells. These findings indicate novel therapeutic targets, including immunotherapeutic, for treatment of OSCC-GB.

## Background

Cancer of oral cavity and lip has the highest incidence (http://cancerindia.org.in/globocan-2018-india-factsheet/) and the highest mortality among men in India [1]. About 90% of the oral malignancies are squamous cell carcinomas (SCC) — one of the most common histologic forms of head and neck squamous cell carcinoma (HNSCC) [2]. Oral squamous cell carcinoma of the gingivo-buccal region (OSCC-GB), comprising buccal mucosa, gingivo-buccal sulcus, lower gingiva, and retromolar trigone, is the major subtype in India [3, 4]. Chewing of betel-quid containing areca nut (a*reca catechu)* with or without tobacco, and also use of tobacco in other smokeless forms are prominent risk factors [4]. Delayed presentation (~ 60% patients report at T3 or T4 stages) due to lack of awareness and a high rate of locoregional recurrence are major impediments to efficient management of oral cancer in India [5]. Currently, the treatment of OSCC-GB is surgical resection followed by adjuvant radiotherapy with or without chemotherapy [2]. There has been no significant decrease in the incidence of oral cancer for many decades [2]. Despite aggressive multi-modality treatment, advanced oral cancer has a high rate of locoregional recurrence and poor prognosis with an overall 5-year survival rate of ~ 60%, which reduces further if associated with metastasis [5]. There is a felt need to identify better prognostic and predictive markers to complement clinico-pathological findings in OSCC-GB.

In addition to genetic alterations, significant epigenetic alterations have been found to be associated with many cancer types [6]. Promoter hypermethylation, mainly in CpG islands, can lead to downregulation of tumor suppressor genes and also dysregulation of downstream signaling pathways; these indications can serve as predictive markers of cancer [7]. Global epigenomic alterations during the initiation and progression of cancer are indeed hallmarks of cancer [8].

We have previously identified a set of driver genes for OSCC-GB, with significantly enhanced burden of somatic mutations and copy number alterations, some of which are unique compared to drivers of other subtypes of head and neck cancers [9, 10]. Unique findings in OSCC-GB have also been reported by transcriptome-wide studies [11, 12]. OSCC-GB, therefore, deserves to be investigated separately. Differential methylation profiles of squamous cell carcinoma of different anatomic sites of the head and neck region have also been described [13–18], but none pertaining to the gingivo-buccal region of oral cavity. Among cancers of the oral cavity, tongue cancer is the most prevalent in western countries. Although OSCC-GB, the predominant type of oral cancer in India, is known to be quite different from tongue cancer in its molecular genetic presentation and its clinical behavior, it is treated identically as tongue cancer. Therefore, in addition to gaining deeper basic insights on epigenomic and transciptomic alterations in OSCC-GB, it is necessary to obtain predictive markers of prognosis and therapy that are specific to OSCC-GB.

## Methods

### Study participants

Tumor and adjacent histologically normal tissue samples were collected in RNAlater, with written informed consent, from 101 OSCC-GB patients (details in Table 1), recruited at ACTREC, RADCH, and CNCI. TNM staging of each tumor sample was done following the 7th edition of the American Joint Committee on Cancer (AJCC) [19]. Because the quality of biospecimens collected from the 101 patients varied, methylation and transcriptomic analyses could be performed on samples from 87 and 72 patients, respectively.

**Table 1:** Demographic and clinical characteristics of gingivo-buccal oral squamous cell carcinoma patients included in this study

Clinical characteristics	Methylation discovery set	Methylation validation set	Expression discovery set	Expression validation set
	(*n* = 43)	(*n* = 44)	*(n* = 36)	(*n* = 36)
Age (in years)				
Range	26–74	26–65	26–70	32–72
Mean	50.81 ± 12.77	48.7 ± 9.9	50.33 ± 11.19	49.08 ± 10.43
< 40	9	7	7	5
40–45	7	11	6	12
46–50	6	8	4	6
51–55	3	7	7	4
56–60	5	5	5	1
> 60	13	6	7	8
Gender				
Male	39	36	31	28
Female	4	8	5	8
Risk-habit				
Tobacco chewing	16	22	17	18
Tobacco chewing and (smoking and/or alcohol)	25	17	15	13
Smoking and/or alcohol	1	4	3	4
None	1	1	1	1
Tumour stage*				
T1	0	10	10	8
T2	12	13	11	10
T3	4	0	1	1
T4	27	21	14	17
Lymph node invasion*				
N0	18	22	17	14
N+	25	22	19	22

*All patients were M0 (no metastasis) at first presentation when tissue samples were collected for analysis

### DNA extraction and bisulfite treatment

DNA was isolated using DNeasy Blood and Tissue Kit (QIAGEN). The purity and concentration was estimated using NanoDrop 2000 (Thermo Fisher Scientific). Approximately 500 ng genomic DNA from each sample was used for sodium bisulfite conversion using the EZ DNA methylation Gold Kit (Zymo Research, USA). Genome-wide DNA methylation was assayed using iScan (Illumina), for paired tumor and adjacent normal samples of 25 patients (all belonging to the “discovery” subset) using the Infinium Human Methylation450 BeadChip and of 62 patients (18 belonging to the “discovery” and 44 to “validation” subsets) using the Infinium MethylationEPIC BeadChip; these chips interrogate 485577 and 865918 CpG sites, respectively, of which 452512 CpG sites are common. For all assays, we followed the manufacturer’s protocols.

### Processing of DNA methylation data

Raw array data (.IDAT files) were analyzed using the R package “minfi” and also using Illumina GenomeStudio Methylation module. The CpG-specific methylation level (*β* value), for each sample, was calculated as a ratio of fluorescent signal intensity of the methylated (*m*) and total of signal intensities from the methylated (*m*) and unmethylated (*u*) alleles as:

$$ \beta =\frac{\mathit{\operatorname{Max}}\left(m,0\right)}{\mathit{\operatorname{Max}}\left(u,0\right)+\mathit{\operatorname{Max}}\left(m,0\right)+\alpha };\kern0.5em 0\le \upbeta \left(\mathrm{unmethylated}\right)\le 1\left(\mathrm{fully}\kern0.3em \mathrm{methylated}\right) $$

We have used the recommendation of the BeadChip manufacturer (Illumina) and set *α* = 100.

To reduce false positive inference, we have ignored any CpG probe (a) for which detection *p* was > 0.01; (b) “NA”- masked value; (c) that mapped to multiple locations on the human reference genome (hg19 with decoy sequence) when aligned using Bowtie 2 (v. 2.3.4.1) with “end-to-end” alignment mode and allowing for maximum 2 mismatches; (d) overlapped with a repetitive element [repeat masker (v. 4.0.5) (http://www.repeatmasker.org/) from UCSC hg19]; (e) polymorphic (MAF > 0.01) SNPs (dbSNP build 150) located within 10 bp of the interrogated CpG site; (f) spanned known regions of small insertions and deletions (indels) in the human genome (UCSC hg19); or (g) was located on a sex chromosome. Both intra-array (Infinium I and Infinium II) and inter-array (between samples) normalizations were performed: (a) subset quantile within array normalization (SWAN) and (b) inter-array quantile normalization (using *β* values).

### Identification of epigenome-wide differential DNA methylation

Of the 87 patients, approximately 50% (43 patients, randomly selected) were used as a discovery cohort and the remaining (44 patients) were used as a validation cohort. We computed *β* values of the 452512 probes common to the two types of beadchip used for the 43 patients. *β* values of the additional 380341 probes for 18 patients whose samples were assayed using the MethylationEPIC BeadChip were computed and considered as “EPIC only” discovery-set. Wilcoxon signed rank test, corrected for multiple testing by the Benjamini-Hochberg method, was performed for each CpG site to test equality of distributions of *β* values between tumor and adjacent normal samples of patients. The CpG sites with Benjamini-Hochberg corrected *p* < 0.05 (merged set) or 0.02 (EPIC only set) and average |Δβ| ≥ 0.2 (tumor vs adjacent normal) were considered as significantly differentially methylated sites (DMPs). The stringency of the criteria used by us to identify significant DMPs is higher than those popularly used [13, 18]; this was done to avoid false positive inferences. A CpG site was considered hypermethylated if average Δ*β* was ≥ 0.2 or hypomethylated if average Δ*β* was ≤ − 0.2. Probes that were significantly differentially methylated in tumors compared with adjacent normal samples were used for the validation. A DMP was considered to be validated if for that DMP, in the validation cohort, the Benjamini-Hochberg corrected *p* was < 0.05 and the average |Δ*β*| was ≥ 0.2 (tumor vs adjacent normal). A significantly differentially methylated region (DMR) was defined as a gene region with a single or multiple unidirectional DMPs. The methylation level of a DMR was quantified as average of *β* values of DMPs that mapped to it.

### RNA extraction and library preparation

RNA sequencing was performed on tumor and paired adjacent normal samples from 72 OSCC-GB patients. From each patient, total RNA was extracted from tumor and normal tissue samples using AllPrep DNA/RNA Mini Kit (QIAGEN). The quality and concentration of isolated total RNA were checked using Agilent 2100 Bioanalyzer and NanoDrop 2000 (Thermo Fisher Scientific). The OD260/OD280 ratio was ≥ 2, and RNA Integrity Number (RIN) was ≥ 7.0 for all sequenced samples. Ribosomal RNA (rRNA) was removed from the RNA samples, using Ribo-Zero Magnetic Kit (epicentre). Sequencing libraries were prepared from rRNA-depleted samples using TrueSeq RNA Sample Preparation Kit (Illumina). Each triplex cDNA library pool was sequenced as 100-bp paired-end on HiSeq-2000 or HiSeq-2500 (Illumina). Protocols suggested by the manufacturers were used for all assays.

### Identification of transcriptome-wide differential gene expression

The 100-bp paired-end reads were aligned to the human reference transcript (build hg19) and reference genome (hg19 with decoy sequence) using the Tuxedo suite (TopHat2 (v. 2.1.1) [20], Bowtie 2 (v. 2.3.4.1) [21]), and SAMtools (v. 0.1.19) [22], with default settings. To minimize false positive inferences, multi-mapped and non-concordant reads were removed using SAMtools [22]. Duplicate reads were identified using MarkDuplicates from PICARD (https://github.com/broadinstitute/picard) software (v 2.17.0) and discarded. Mapped transcripts were assembled using Cufflinks (v. 2.2.1) [23], with default parameters.

The set of 72 patients were randomly split into two equal subsets; these subsets were separately used for discovery and validation. Normalized gene expression (FPKM) values were estimated for the samples in both discovery and validation cohorts (each cohort *n* = 36) using cuffnorm. In the discovery cohort (*n* = 36), differential gene expression analysis between tumor and normal pairs was performed using cuffdiff with default values. Genes discovered to be significantly differentially expressed were considered for validation. Such a gene was declared as validated if the paired sample *t* test to compare mean expression levels in tumor and normal was significant (*p* < 0.05, after Benjamini-Hochberg multiple-testing correction).

### Integrative analysis of DNA methylation and gene expression

To investigate the correlation between DNA methylation and gene expression in OSCC-GB patients, we have considered only those genes for which differential methylation in the promoter and differential expression level were both statistically significant. Such a gene was further examined to ascertain whether there was an opposite relationship between mean values of promoter methylation and gene expression in tumors compared to normals (i.e., hypomethylation and overexpression, or hypermethylation and underexpression). A validated gene that did not satisfy this property was discarded from further analysis. For each accepted gene, the Spearman correlation coefficient (*ρ*) was then calculated between the methylation level of DMRs (*β* values) and expression (FPKM) values observed in the patients. Genes for which the correlation coefficients were significant (Benjamini-Hochberg multiple-testing corrected *p* < 0.05) were used in pathway enrichment analysis.

### Pathway enrichment analysis

A gene found to be significantly dysregulated and differentially methylated in the promoter was functionally annotated using the ClueGO plugin of Cytoscape (v. 3.7.0) [24]. Gene Ontology (GO) terms and KEGG pathways were used in this analysis. To obtain significantly enriched categories of the GO terms and KEGG pathways, right-sided hypergeometric tests were performed; *p* values were corrected for multiple-testing using the Benjamini-Hochberg method.

### Relationship with drug-induced gene expression

To investigate whether expression levels of genes found epigenetically altered can be altered in the opposite direction by a known drug, we have mined the Comparative Toxicogenomics Database (http://ctdbase.org/) of the DSigDB conglomerate database (http://tanlab.ucdenver.edu/DSigDB/DSigDBv1.0/).

## Results

### DNA methylation and expression patterns

**Fig. 1:**
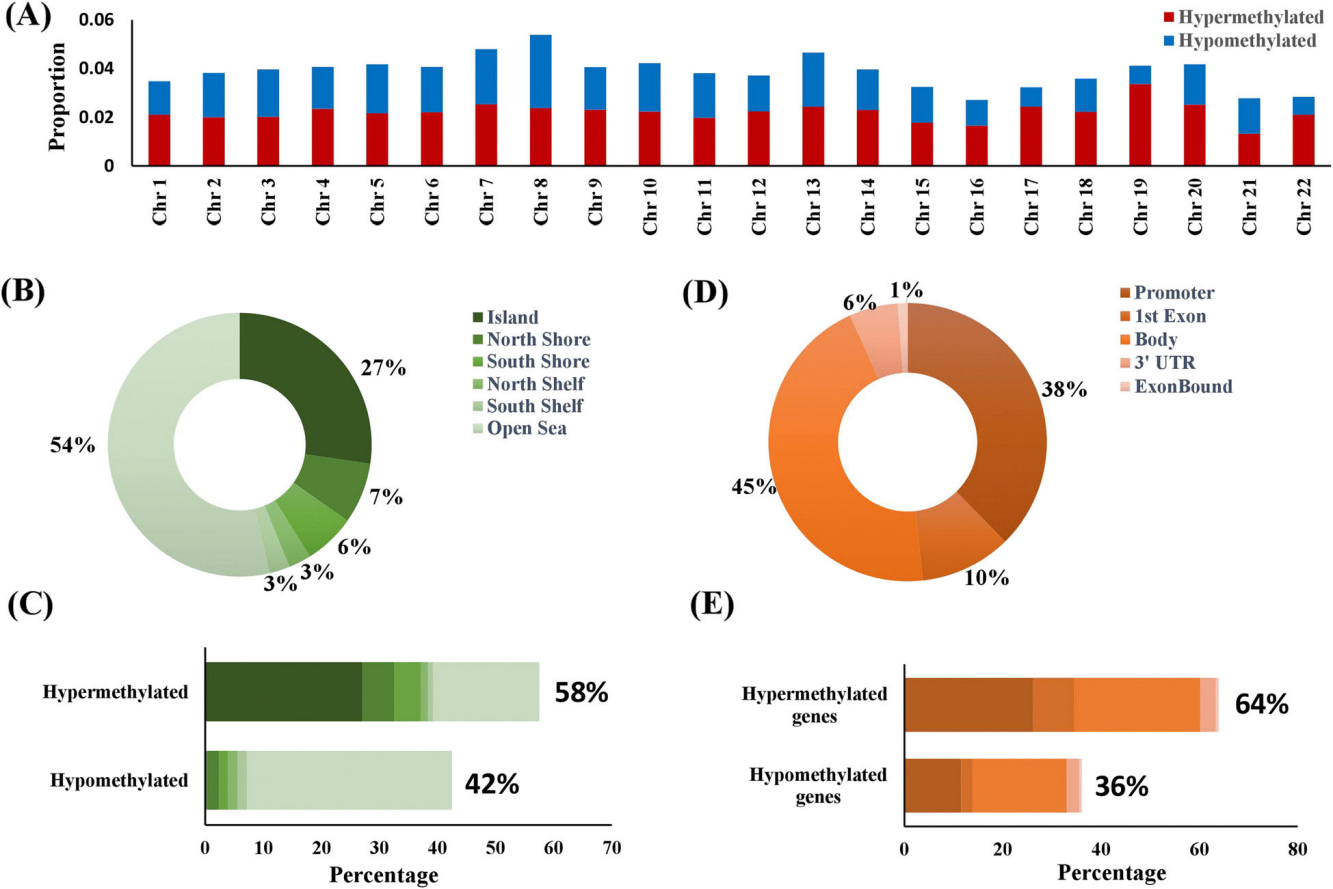
Distribution of differentially methylated probes (DMPs) (*n* = 20023) and regions (DMRs) (*n* = 4861). Percentage distribution of **a** hyper- and hypo-methylated DMPs on different autosomal chromosomes, **b** percentages of all DMPs located in different genomic regions, and **c** separately for DMPs that were hyper- and hypo-methylated. **d** Percentages of all DMRs located in different gene regions and **e** separately for DMRs that were hyper- and hypo-methylated

Epigenome-wide DNA methylation, assayed on paired tumor and adjacent normal tissue samples from 43 OSCC-GB patients (discovery cohort) and validated in an independent set of 44 patients (validation cohort), identified 25321 significant DMPs, of which 20023 were validated, of which 11387 were present on both Infinium 450 K and EPIC BeadChip arrays, while the remaining 8636 DMPs were present on EPIC BeadChip array only. Of these 20023 validated DMPs, 11522 (~ 58%) were hypermethylated and 8501 (~ 42%) were hypomethylated (Fig. 1). There is considerable variation in the proportion of DMPs across chromosomes; this proportion was highest for chromosome 8 and lowest for chromosome 16. With the exception of chromosomes 8 and 21, on all other chromosomes, the proportion of hypermethylated DMPs was higher than those hypomethylated (Fig. 1a); particularly high proportions of hypermethylated DMPs were observed for chromosomes 19 (82%), 17 (75%), and 22 (74%). Distinctly different patterns were observed between CGI (CpG islands) and non-CGI regions of the genome. Nearly half of the DMPs (~ 54%; 10744 of 20023) were enriched in non-CGI, open sea region of the genome. While about two-thirds of the DMPs in non-CGI regions were hypomethylated, almost all (~ 99%) DMPs located in the CGIs (~ 27% of all DMPs) were hypermethylated (Fig. 1b, c).

About 23% (4653 of 20023) of all DMPs mapped to promoter regions [1500 nt upstream of transcription start site (TSS) and from TSS to first exon] of known protein-coding genes. DNA methylation profiling identified and validated 6156 gene-regions that map to 4861 protein-coding genes. (It may be noted that multiple gene-regions can map to the same gene.) Significant differential methylation was observed in the promoter regions of 2318 genes, of which 1606 were hypermethylated and 712 were hypomethylated. About 38% (= 2318 of 6156) differentially methylated gene-regions belong to the promoter region of genes (Fig. 1d, e).

We discovered 2207 protein-coding genes to be significantly differentially expressed, at least by two-fold (mean of |log2 (fold change)| > 1), in tumors compared with paired normal samples, of which 1734 (78.6%) genes [804 (~ 46%) upregulated and 930 (~ 54%) downregulated] were validated (corrected *p* < 0.05; mean of |log2 (fold change)| > 1). Of the 1734 significantly differentially expressed genes, the maximum number of genes were on chromosome 1 (184 genes), followed by chromosomes 2 (127), 19 (125), 3 (110), and 11 (109).

### Significant alteration of gene expression by epigenetic alteration associated with OSCC-GB

We validated that 307 genes were both significantly differentially methylated in promoters and also significantly differentially expressed. Of these, 209 genes were found to have significant negative correlation between *β* and FPKM values (Additional file 1: Table S1). Of these 209 genes, epigenetic downregulation due to significant promoter methylation was observed in 156 genes. The 10 genes with strongest negative correlation included *ZNF132*, *ZNF626*, *ZSCAN18*, *ZNF844*, *SH2D2A*, *PHYHD1*, *IGF2BP2*, *ZNF829*, *ZNF880*, and *ZNF229* (Additional file 1: Table S1)*.* Except *SH2D2A* and *IGF2BP2*, all genes were epigenetically downregulated.

**Fig. 2:**
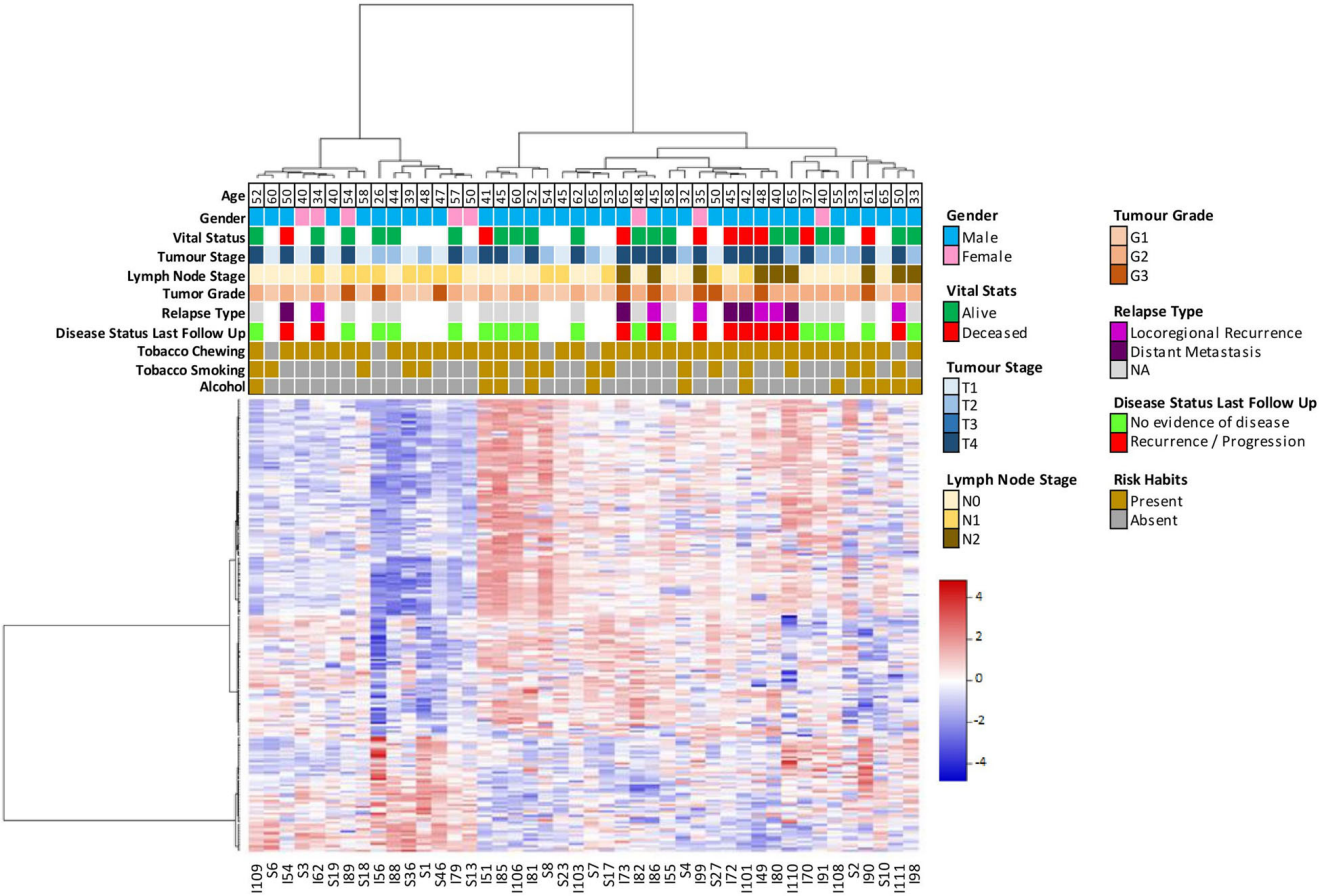
Integrated unsupervised hierarchical clustering and heatmap using Δ*β* values of differentially methylated genes in promoter region from OSCC-GB patients depicts two major clusters of patients with distinct phenotypic features. (white box in the top panel indicates unavailability of the respective clinical information)

Unsupervised hierarchical cluster analysis by Ward’s method using the Δ*β* values of 209 significantly differentially methylated genes identified two major clusters of patients (Fig. 2), comprising 15 and 29 patients, respectively. We note that the proportion of patients (55%) in the larger cluster who belongs to higher stages (T3 and T4) is greater compared to patients in the smaller cluster (33%). Further, about a third of the patients in the larger cluster presented with lymph node metastasis (N2); none of the patients in the smaller cluster presented with metastasis (Fig. 2).

### Drug-induced reversal of epigenetic dysregulation

Of the 209 genes that we identified to be epigenetically dysregulated, the expression levels of 148 genes (Additional file 2: Table S2) are altered by known drugs in the direction opposite to that of epigenetic alteration; the total number of such expression-altering drugs is ~ 350. Of all significantly epigenetically downregulated (upregulated) genes in OSCC-GB, known drugs could upregulate (downregulate) 105 (43) genes.

### Enriched pathways in OSCC-GB patients

**Fig. 3:**
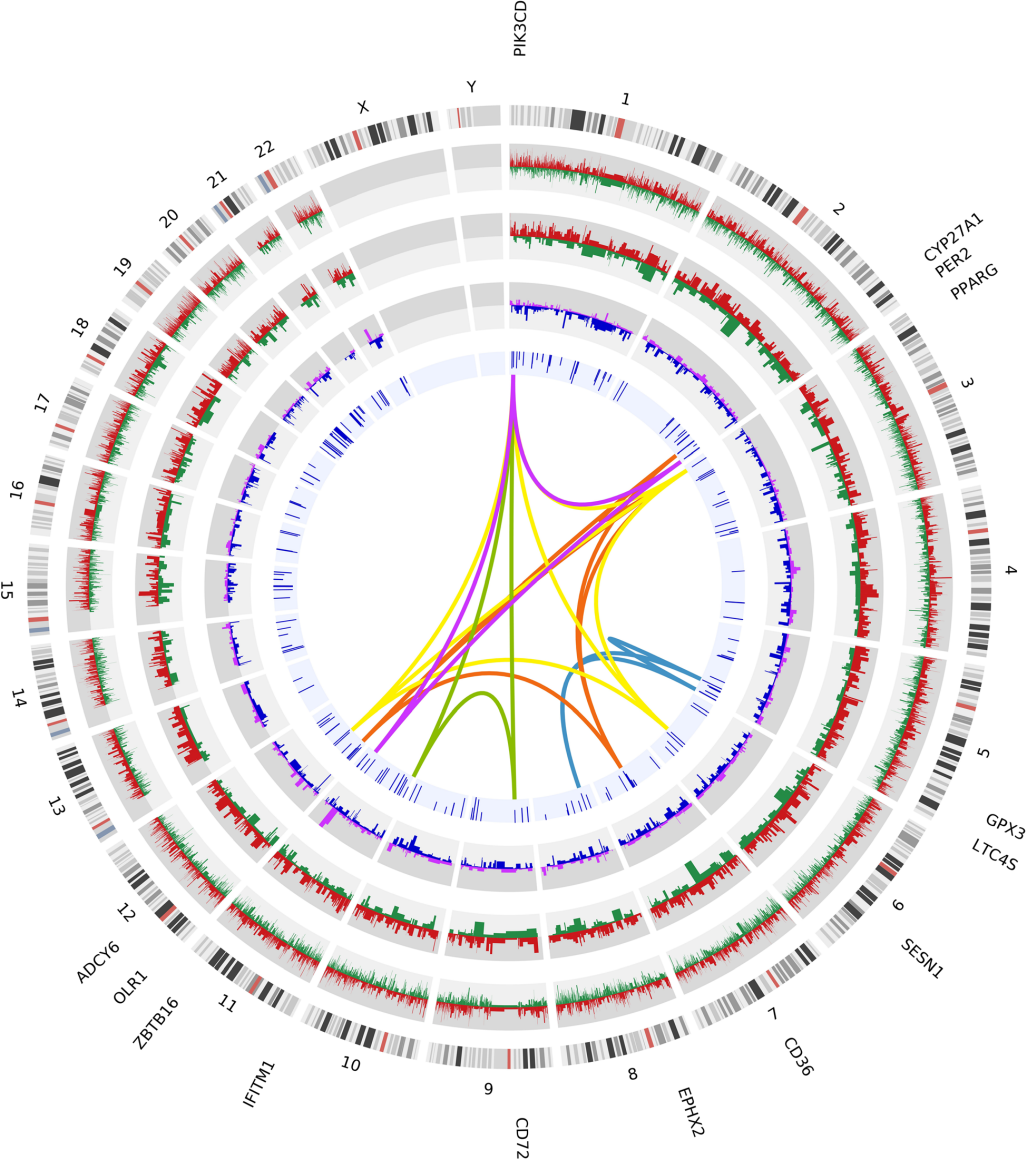
An integrative circos plot of epigenomic and transcriptomic alterations in OSCC-GB. The outermost track displays the human genome (hg19) ideogram by chromosome number. The second track depicts frequency distribution of epigenome-wide significantly differentially methylated CpG sites. The third track provides the distribution of coding genes, with differential methylation in their promoter regions. Hypermethylation and hypomethylation are represented by red and green colors, respectively. The fourth track presents transcriptomic profiles in autosomes—upregulated and downregulated genes are shown in purple and blue, respectively. The inner most track represents the 209 genes that showed significant inverse correlation between promoter methylation and expression. Of these 209, 36 (17.2%) genes are on chromosome 19. Heights of blue bars are proportional to the correlation coefficients. The color-coded links represent genes from the significantly dysregulated pathways (*n* = 5). Gene names from the pathways are shown outside the ideogram. (Cyan, arachidonic acid metabolism; Yellow, longevity regulating pathway; Orange, PPAR signaling pathway; Green, B cell receptor signaling pathway; Purple, acute myeloid leukemia)

Pathway enrichment analyses using 209 significantly dysregulated genes associated with epigenetic modifications in their promoter regions identified significant (corrected *p* < 0.05) enrichment of five KEGG pathways (Table 2 and Fig. 3) and twenty-two GO terms (Additional file 3: Table S3). Significantly enriched KEGG pathways included PPAR signaling (earlier found with significant gene expression alterations in OSCC-GB [15] and OSCC-tongue [25]), arachidonic acid metabolism (earlier implicated in OSCC using transcriptomic data [26] and in OSCC-GB on post-treatment disease-free survival length using somatic mutation data [27]), and B cell receptor signaling pathway (commonly implicated in chronic lymphocytic leukemia [28], but also in OSCC [29]). The enriched GO terms included negative regulation of G0 to G1 transition, process related to apoptosis such as regulation of leukocyte apoptotic process or negative regulation of dendritic cell apoptotic process, chemokine-mediated signaling pathway, and many other immune-related processes, including natural killer cell-mediated cytotoxicity, type I interferon signaling pathway. (Additional file 3: Table S3). The negative regulation of the cell cycle during G0 and G1 phases was shown in a previous study on OSCC [30]. The chemokine-mediated signaling pathway was earlier found altered in lung cancer [31].

**Table 2:** Significantly enriched pathways in OSCC-GB patients based on 209 genes significantly differentially methylated in their promoter regions and related information

KEGG pathway	No. genes	% Associated genes	Corrected *p* value	Names of associated genes
PPAR signaling pathway	4	5.41	0.036	*CD36*, *CYP27A1*, *OLR1*, *PPARG*
Arachidonic acid metabolism	3	4.76	0.046	*EPHX2*, *GPX3*, *LTC4S*
Acute myeloid leukemia	3	4.55	0.038	*PER2*, *PIK3CD*, *ZBTB16*
Longevity regulating pathway	4	4.49	0.034	*ADCY6*, *PIK3CD*, *PPARG*, *SESN1*
B cell receptor signaling pathway	3	4.23	0.037	*CD72*, *IFITM1*, *PIK3CD*

### Comparison with TCGA data

The genome-wide DNA methylation profile observed in the OSCC-GB patients was validated using publicly available TCGA HNSCC data. In TCGA, all sub sites of HNSCC were included. There is considerable heterogeneity in regional presentation within the oral cavity [16]. We have, therefore, considered data pertaining to the 31 OSCC-GB patients in the TCGA dataset (raw .IDAT files from the GDC Legacy Archive). In TCGA, OSCC-GB data were generated using the HM-450 K array while samples of our validation cohort patients were assayed using the HM-EPIC array platform. We, therefore, considered only those DMPs (CpG mapped to the promoter region of genes) that were common (80.4% of all promoter DMPs) to both arrays. Spearman’s correlation between *β* values of the 3741 common promoter DMPs of TCGA (*n* = 31) and our data (*n* = 44) of OSCC-GB patients was very high [*ρ* = 0.8574; *p* < 2.2 × 10^−16^] (Additional file 4: Figure S1). Patterns of clustering of patients based on *β* values of the common promoter DMPs were, therefore, largely similar (Additional file 4: Figure S2), thereby providing greater confidence to the inferences drawn from our data.

## Discussion

Gingivo-buccal oral squamous cell carcinoma, an anatomical and clinical subtype of head and neck squamous cell carcinoma (HNSCC) is the leading cancer among men in India. Epigenomic reprogramming, more specifically epigenomic gene repression, is a significant hallmark of oral cancer. Epigenome-wide DNA methylation profiling of 87 OSCC-GB patients (43 patients used as a discovery cohort and the remaining 44 as the validation cohort), using DNA methylation microarrays, identified 20023 significantly differentially methylated tumor-specific DMPs that mapped to 4861 protein-coding genes. Of these genes, 1606 were hyper- and 712 hypo-methylated in the promoter regions. Gene expression was significantly negatively correlated with DNA methylation in the promoter regions of 209 genes, of which 156 (~ 75%) were found epigenetically downregulated.

The ten genes with that showed high significant negative correlation between promoter methylation level and gene expression included mostly (7 of 10) zinc finger protein genes (Z*NF132*, *ZNF626*, *ZSCAN18*, *ZNF844*, *ZNF829*, *ZNF880*, and *ZNF229*) located on chromosome 19 (19q13; *ZNF626* on 19p12)*.* These genes encode C2H2-type of zinc finger proteins. Except ZNF880, all six zinc finger proteins are Krüppel-type family of transcription factors. A cluster of Krüppel-type zinc finger protein genes (including *ZNF132* and *ZSCAN18*, earlier known as *ZNF447*) on chromosome 19q13 was earlier found significantly epigenetically downregulated in oropharyngeal squamous cell carcinoma [32]. The remaining three genes are *SH2D2A*, *PHYHD1*, and *IGF2BP2*, of which *IGF2BP2* is noteworthy. *IGF2BP2* encodes an insulin-like growth factor-2 mRNA-binding protein and belongs to a conserved family of RNA-binding, oncofetal proteins. This protein modulates cell polarization, adhesion, and migration in tumor-derived cells and is strongly associated with cancer metastasis and the expression of oncogenic factors that include KRAS, MYC, and MDR1 [33]. Upregulation of *IGF2BP2* is associated with HNSCC [34]. *ZNF829* hypermethylation is associated with colorectal cancer [35]. Overall, methylation of transcription factor and RNA binding genes and consequent alteration of expression seem important correlates of OSCC-GB.

Other significantly epigenetically downregulated genes are *OSR1*, *SELENBP1*, *TGFBR3*, and *ZBTB16* [36–39]. Among significantly downregulated genes with promoter hypermethylation, *CDON*, *ID4*, and *CPEB1* were associated with neuroblastoma, breast cancers, and hepatocellular carcinoma, respectively [40–42]. Upregulation with significant promoter hypomethylation of *CD274*, earlier known as *PD-L1*, was observed to be associated with OSCC-GB. Immunoexpression of the protein encoded by *PD-L1* was found significantly increased in OSCC patients compared with normal or oral leukoplakia (oral pre-cancer) [43]. Upregulation of another immune checkpoint gene *CD80*, observed in oral cancer [18], was found to be driven by significant promoter hypomethylation in OSCC-GB patients. *CTLA4* that encodes an immune checkpoint receptor, was found to be transcribed in OSCC-GB tumour tissue by five- to six-fold (adjusted *p* < 0.05) higher than normal tissue (six-fold higher in discovery cohort; five-fold higher in validation cohort). However, this upregulation could not be ascribed to promoter hypomethylation of *CTLA4* in our patients, contrary to the finding of a previous study on OSCC [18]. Our observation of significant epigenetic upregulation of *TRPM2* and *LCK* is consistent with earlier reports on oral cancer and oral lichen planus (oral pre-cancer) [44, 45]. We have also observed epigenetic upregulations of *SULF1* and *SEMA3C* earlier found upregulated in gastric cancer [46] and breast cancer [47], respectively. However, our finding of upregulation of *SEMA3C* is not consistent with an earlier report on oral cancer [47]. Our observations that *DNMT3B*, a gene responsible for de novo methylation process, is significantly upregulated with promoter hypomethylation and *TET1*, involved in DNA methylation process and gene activation, and is downregulated with promoter hypermethylation are worthy of further consideration.

We have also replicated some earlier observations on oral cancer and oral pre-cancer and in other cancers. These include epigenetic downregulation of *GAS7*, *OSR1*, *SELENBP1*, *TGFBR3*, and *ZBTB16*, earlier found associated with OSCC/HNSCC; epigenetic upregulation of *TRPM2* and *LCK* (a proto-oncogene), earlier observed overexpressed in patients with OSCC or oral lichen planus (oral pre-cancer); altered expression of epigenetically downregulated (C*DON*, *ID4*, *ZSCAN18*, *CPEB1*, and *NUPR1*) or epigenetically upregulated (S*ULF1* and *SEMA3C)* genes earlier associated with several cancer types.

An important result of our study is that the immune checkpoint gene *CD274* (earlier known as *PD-L1*) is significantly upregulated due to significant promoter hypomethylation. The protein encoded by *PD-L1*, a ligand of PD-1 receptor expressed on the surface of activated T cells, is expressed on the surface of antigen-presenting cells (APCs) which mostly include dendritic cells or macrophages. The induction of PD-L1 expression on the surface of tumor cells helps them evade immune response inhibition of the cytotoxic T cells and hence preventing apoptosis by the immunosuppressive properties of PD-L1/PD-1 interactions in the tumor microenvironment [48]. Immunotherapy against immune checkpoints PD-L1 has shown promising results in treating metastatic HNSCC patients [49]. Therefore, our finding prompts consideration of PD-1/PD-L1 immune checkpoint as a potential target for cancer therapy in OSCC-GB patients. The programmed death ligand 2 or PD-L2 (now known as PDCD1LG2) along with PD-L1 interacts with PD-1 to inhibit T cell activation and to downregulate the expression of anti-apoptotic molecules and the production of pro-inflammatory cytokines [48]. We have observed significant upregulation of *PDCD1LG2* in tumor samples. However, differential expression of this gene is driven by mechanisms other than differential methylation in OSCC-GB patients. In a recent study, it was shown that cis-PD-L1/CD80 interactions can disrupt PD-L1/PD-1 binding [50]. This study [50] also observed that the binding was not affected by the simultaneous overexpression of CD80 and CD86. Similar to the previous study on OSCC patients [18], we have observed significant upregulation of *CD80*—driven by promoter hypomethylation—and *CD86* in OSCC-GB patients. In addition, we have identified significant upregulation of *CTLA4*, and no significant alteration of expression or promoter DNA methylation of *CD28* (which promotes the T cell activation). Therefore, in OSCC-GB patients, ligand-receptor interactions, such as, PD-L1/PD-1 or PD-L2/PD-1 and CTLA4/CD80 or CTLA4/CD86 may have crucial role in suppressing the effector T cell responses and hence immune evasion. These findings provide cues to further investigation that may be indicative of deployment of immune therapy in OSCC-GB.

We have noted upregulation of *DNMT3B* in OSCC-GB tumor samples driven by promoter hypomethylation. The product of *DNMT3B* acts as de novo DNA methyltransferase by transferring a methyl group from S-adenosylmethionine to the C-5 position of cytosine to form 5-methylcytosine (5mC), in the absence of a template [51]. Overexpression of *DNMT3B* often correlates with the epigenetic inactivation of tumor suppressor genes leading to tumor formation [52], including oral tumor [53]. On the contrary, the protein encoded by *TET1* acts as demethylase which plays a key role in DNA demethylation by catalyzing the conversion of 5mC into 5-hydroxymethylcytosine (5hmC). It also mediates subsequent conversion of 5hmC into 5-formylcytosine (5fC) and 5-carboxylcytosine (5caC), which are further converted to unmethylated cytosine through the base excision repair pathway [54]. Loss of *TET1* expression through promoter CpG methylation frequently occurs in tumor cells, which results in tumor pathogenesis via inactivation of tumor suppressor genes [55]. Epigenetic downregulation of *TET1*, observed in OSCC-GB patients, was earlier found associated with head and neck cancer [56]. Therefore, our finding that epigenetic modifications upregulate *DNMT3B* and downregulate *TET1*, that likely plays an important role in OSCC-GB tumorigenesis, is novel.

We have identified that expression levels of about 71% (148 of 209) of the genes that are epigenetically dysregulated in OSCC-GB could be reversed by known drugs (Additional file 2: Table S2). The genes whose expression levels were altered by drugs included *CD274*, *CD80*, *DNMT3B*, *TET1*, *PPARG*, and *PIK3CD.* The drugs that induced reversal of expressions of these genes include dasatinib, calcitriol, tamoxifen, and aspirin. It is interesting that OSCC-GB caused by epigenetic alterations in important cancer genes is potentially actionable by known drugs.

Pathway analysis based on 209 significantly differentially genes identified PPAR signalling, arachidonic acid metabolism, and B cell receptor signaling pathways. The peroxisome proliferator-activated family of receptors (PPARs) are ligand-activated transcription factors. Epigenetic downregulation of *PPARG* (from the PPAR signaling pathway) and its ligands have earlier been shown to play a role in tumorigenesis. We have observed that *PPARG* is dysregulated in OSCC-GB patients and earlier also reported as dysregulated in HNSCC patients [57–59]. As a chemo-preventive agent, PPARG ligands act as targets for several types of cancer [57]. Synthetic PPARG ligands have been shown to decrease incidence of carcinogen-induced tongue tumors in a dose-dependent manner [59]. DNMT inhibitors (DNMTIs) are a promising class of anti-cancer therapies that renew transcription of a previously silenced gene [60]. As a matter of fact, DNMTIs are being used to reverse epigenetic downregulation of PPARG, instead of PPARG ligands that show cytotoxicity. We have earlier shown [28] that survival benefits accrue to OSCC-GB patients who carry mutations in genes of the arachidonic acid metabolism (AAM) pathway. Our present finding of downregulation of oxidative stress-related genes *EPHX2* and *GPX3*, belonging to the AAM pathway, in OSCC-GB patients, is therefore notable; similar findings were also reported in cancer of the oral cavity [61]. Some genes that we found to be epigenetically upregulated in OSCC-GB patients, such as *PIK3CD* and *IFITM1*, were earlier reported to be upregulated in thyroid cancer [62] and HNSCC [63]. IFITM1 plays an important role in invasion at early stages of HNSCC [63].

In OSCC-GB patients, we have observed a strong positive correlation of methylation levels of promoter DMPs between our study and the TCGA HNSCC study. Clusters of OSCC-GB patients of this study and patients included in TCGA, based on promoter DMPs that were common between the two studies, were similar. This is indicative of the robustness of the inferences of this study.

## Conclusions

In sum, we have identified epigenetic alterations of key genes that regulate the feedback process of DNA methylation for the maintenance of normal cell division. Epigenetic alteration of transcription factor genes and RNA-binding genes and consequently expression dysregulation were strongly associated with OSCC-GB. Epigenetic activation of immunosuppressive PD-L1/PD-1 and CD80/CTLA4 interactions, which leads to inhibition of the cytotoxic T cells-mediated apoptotic process, was observed in gingivo-buccal oral cancer patients. Signaling (PPAR and B cell receptor) and the arachidonic acid metabolism pathways were found enriched in OSCC-GB. Our results provide novel therapeutic targets, including immunotherapy, for treatment of gingivo-buccal oral squamous cell carcinoma.

## Supplementary information

**Additional file 1: Table S1**. Relevant details of 209 genes with significant negative correlation between promoter methylation and gene expression.

**Additional file 2: Table S2**. Information related to alteration of expression of 209 epigenetically dysregulated genes by one or more known drugs.

**Additional file 3: Table S3.** Significantly enriched GO terms in OSCC-GB patients based on 209 genes significantly differentially methylated in their promoter regions and related information.

**Additional file 4: Figure S1**. Scatter diagram showing the relationship of average β values of the DMPs found in the present study with those in the TCGA study, irrespective of whether these probes were also significantly differentially methylated in the TCGA study. Each point on the scatter diagram indicates for a DMP of the present study the average β value over the 44 OSCC-GB patients included in the validation subset and, for the TCGA study, averaged over the 31 OSCC-GB patients. **Figure S2**. Integrated unsupervised hierarchical clustering and heatmap using quantitative methylation level of 3741 DMPs mapped to gene promoter from (A) 44 OSCC-GB patients included in this study and (B) 31 OSCC-GB patients included in TCGA study. Corresponding clusters obtained from two sets of patients showed similar methylation pattern and phenotypic features. [White box in top panels of (A) and (B) indicates unavailability of the respective clinical information].

## Data Availability

Raw IDAT files of 174 samples generated using Illumina Infinium methylation array were deposited under EGAS00001003896 EGA study ID and aligned bam files for transcriptome data of 144 samples were deposited under EGAS00001003893 EGA study ID. Biospecimens may be shared on request, if available. The requester has to obtain prior approval from the Government of India to obtain biospecimens.

## Abbreviations

5caC: 5-Carboxylcytosine
5fC: 5-Formylcytosine
5hmC: 5-Hydroxymethylcytosine
5mC: 5-Methylcytosine
APC: Antigen-presenting cell
cDNA: Complementary DNA
CGI: CpG island
DMP: Differentially methylated probe
DMR: Differentially methylated region
DNMTI: DNMT inhibitor
FPKM: Fragments per kilobase of transcript per million mapped reads
GO: Gene ontology
HNSCC: Head and neck squamous cell carcinoma
KEGG: Kyoto Encyclopedia of Genes and Genomes
MAF: Minor allele frequency
OSCC: Oral squamous cell carcinoma
OSCC-GB: Gingivo-buccal oral squamous cell carcinoma
PPAR: Peroxisome proliferator-activated receptor
RIN: RNA Integrity Number
rRNA: Ribosomal RNA
SCC: Squamous cell carcinomas
SWAN: Subset quantile within array normalization
TCGA: The Cancer Genome Atlas
TSS: Transcription start site
