# Supplementary material for: Epigenomic dysregulation-mediated alterations of key biological pathways and tumor immune evasion are hallmarks of gingivo-buccal oral cancer
Source: Clin Epigenetics. 2019 Dec 3;11:178. doi: 10.1186/s13148-019-0782-2 (PMC6889354; doi:10.1186/s13148-019-0782-2)
Supplement: Supplementary file 1 — Additional file 1: Table S1. Relevant details of 209 genes with significant negative correlation between promoter methylation and gene expression. [file 13148_2019_782_MOESM1_ESM.docx]

**Supplementary Table S1: Relevant details of 209 genes with significant negative correlation between promoter methylation and gene expression**

| **Gene** | **Average Δβ** | **Average log2(fold-change)** | **Spearman’s ρ** | **BH adjusted**  **p-value** |
| --- | --- | --- | --- | --- |
| *ZNF132* | 0.319 | -1.732 | -0.871 | <1x10-14 |
| *ZNF626* | 0.241 | -2.078 | -0.863 | <1x10-14 |
| *ZSCAN18* | 0.244 | -2.333 | -0.860 | <1x10-14 |
| *ZNF844* | 0.288 | -2.215 | -0.857 | <1x10-14 |
| *SH2D2A* | -0.283 | 2.467 | -0.857 | <1x10-14 |
| *PHYHD1* | 0.307 | -2.944 | -0.852 | <1x10-14 |
| *IGF2BP2* | -0.258 | 2.570 | -0.851 | <1x10-14 |
| *ZNF829* | 0.280 | -1.323 | -0.841 | <1x10-14 |
| *ZNF880* | 0.300 | -2.085 | -0.839 | <1x10-14 |
| *ZNF229* | 0.266 | -2.409 | -0.836 | <1x10-14 |
| *ZFP28* | 0.320 | -1.944 | -0.835 | <1x10-14 |
| *NDRG2* | 0.266 | -2.476 | -0.835 | <1x10-14 |
| *BST2* | -0.267 | 2.590 | -0.824 | <1x10-14 |
| *RRM2* | -0.244 | 1.738 | -0.820 | <1x10-14 |
| *TENC1* | 0.276 | -1.715 | -0.820 | <1x10-14 |
| *GPX3* | 0.289 | -2.466 | -0.818 | <1x10-14 |
| *ZNF582* | 0.303 | -1.539 | -0.817 | <1x10-14 |
| *DSE* | -0.237 | 1.203 | -0.815 | <1x10-14 |
| *NYNRIN* | 0.263 | -1.485 | -0.814 | <1x10-14 |
| *CLDN11* | 0.245 | -2.504 | -0.812 | <1x10-14 |
| *TNS1* | 0.270 | -1.990 | -0.808 | <1x10-14 |
| *ZNF790* | 0.260 | -1.459 | -0.806 | <1x10-14 |
| *GSN* | 0.243 | -1.372 | -0.805 | <1x10-14 |
| *ZNF135* | 0.313 | -1.909 | -0.803 | <1x10-14 |
| *IL21R* | -0.248 | 2.060 | -0.803 | <1x10-14 |
| *SVIP* | 0.286 | -2.088 | -0.803 | <1x10-14 |
| *OSR1* | 0.225 | -3.545 | -0.800 | <1x10-14 |
| *ZNF471* | 0.324 | -2.556 | -0.798 | <1x10-14 |
| *COX7A1* | 0.246 | -3.460 | -0.798 | <1x10-14 |
| *CCRL2* | -0.223 | 1.054 | -0.798 | <1x10-14 |
| *ZNF568* | 0.367 | -1.845 | -0.798 | <1x10-14 |
| *ZNF570* | 0.268 | -1.410 | -0.798 | <1x10-14 |
| *NMI* | -0.328 | 1.483 | -0.797 | <1x10-14 |
| *SMIM3* | -0.246 | 1.456 | -0.793 | <1x10-14 |
| *ZNF677* | 0.250 | -2.008 | -0.792 | <1x10-14 |
| *ZNF420* | 0.285 | -1.666 | -0.788 | <1x10-14 |
| *CHADL* | 0.227 | -2.191 | -0.785 | <1x10-14 |
| *ZNF667* | 0.228 | -2.044 | -0.784 | <1x10-14 |
| *IL34* | 0.226 | -2.445 | -0.782 | <1x10-14 |
| *GPR68* | -0.204 | 1.236 | -0.781 | <1x10-14 |
| *EPHX1* | 0.290 | -1.236 | -0.779 | <1x10-14 |
| *ZNF737* | 0.269 | -1.349 | -0.779 | <1x10-14 |
| *NFIX* | 0.316 | -2.103 | -0.779 | <1x10-14 |
| *KLHL23* | 0.233 | -1.124 | -0.777 | <1x10-14 |
| *ITIH5* | 0.276 | -1.553 | -0.775 | <1x10-14 |
| *ZNF569* | 0.277 | -1.367 | -0.772 | <1x10-14 |
| *ZNF528* | 0.300 | -1.637 | -0.771 | <1x10-14 |
| *ADCY6* | 0.311 | -1.392 | -0.771 | <1x10-14 |
| *SORBS2* | 0.261 | -2.197 | -0.765 | <1x10-14 |
| *FAM63A* | 0.326 | -1.190 | -0.764 | <1x10-14 |
| *CYP2R1* | 0.283 | -1.171 | -0.764 | <1x10-14 |
| *PAMR1* | 0.215 | -1.482 | -0.763 | <1x10-14 |
| *HKR1* | 0.242 | -1.122 | -0.761 | <1x10-14 |
| *PHC1* | 0.269 | -1.276 | -0.759 | <1x10-14 |
| *SEMA3C* | -0.210 | 1.387 | -0.758 | <1x10-14 |
| *NR3C2* | 0.227 | -3.339 | -0.756 | <1x10-14 |
| *PALM* | 0.220 | -2.152 | -0.755 | <1x10-14 |
| *PYGO1* | 0.261 | -1.900 | -0.754 | <1x10-14 |
| *CGNL1* | 0.271 | -3.349 | -0.753 | <1x10-14 |
| *DLGAP4* | -0.264 | 1.114 | -0.751 | <1x10-14 |
| *ZNF415* | 0.338 | -2.146 | -0.751 | <1x10-14 |
| *ZNF354C* | 0.223 | -1.531 | -0.751 | <1x10-14 |
| *MYEF2* | 0.274 | -1.629 | -0.750 | <1x10-14 |
| *MLPH* | 0.285 | -4.518 | -0.750 | <1x10-14 |
| *LTC4S* | 0.293 | -1.235 | -0.749 | <1x10-14 |
| *ZSCAN16* | 0.218 | -1.119 | -0.747 | <1x10-14 |
| *PCYT1A* | -0.229 | 1.033 | -0.747 | <1x10-14 |
| *SESN1* | 0.356 | -1.231 | -0.740 | <1x10-14 |
| *LAMA3* | -0.289 | 2.834 | -0.737 | <1x10-14 |
| *SELENBP1* | 0.302 | -3.381 | -0.733 | <1x10-14 |
| *GFRA1* | 0.294 | -3.021 | -0.731 | <1x10-14 |
| *MKI67* | -0.232 | 1.740 | -0.730 | <1x10-14 |
| *ID4* | 0.223 | -2.231 | -0.730 | <1x10-14 |
| *MX2* | -0.259 | 1.874 | -0.729 | <1x10-14 |
| *CHPT1* | 0.322 | -2.435 | -0.728 | <1x10-14 |
| *DAGLA* | 0.320 | -1.721 | -0.726 | <1x10-14 |
| *TGFBR3* | 0.333 | -2.495 | -0.726 | <1x10-14 |
| *ZNF491* | 0.239 | -1.887 | -0.725 | <1x10-14 |
| *OAS2* | -0.225 | 2.353 | -0.723 | <1x10-14 |
| *FBXL8* | 0.202 | -1.061 | -0.722 | <1x10-14 |
| *ACSS3* | 0.260 | -2.777 | -0.721 | <1x10-14 |
| *AMPD3* | -0.231 | 1.124 | -0.717 | <1x10-14 |
| *LRFN4* | -0.255 | 1.646 | -0.717 | <1x10-14 |
| *NRIP2* | 0.236 | -1.423 | -0.712 | 1.20x10-14 |
| *PGAP3* | 0.278 | -1.114 | -0.710 | <1x10-14 |
| *NTRK3* | 0.319 | -3.097 | -0.709 | <1x10-14 |
| *PDK4* | 0.222 | -3.124 | -0.708 | <1x10-14 |
| *GZMB* | -0.248 | 2.387 | -0.706 | <1x10-14 |
| *DNMT3B* | -0.220 | 2.473 | -0.705 | 2.69x10-14 |
| *ZNF345* | 0.284 | -1.345 | -0.705 | <1x10-14 |
| *MMP13* | -0.223 | 9.157 | -0.702 | <1x10-14 |
| *ZBTB47* | 0.249 | -1.478 | -0.701 | <1x10-14 |
| *NCAM2* | 0.235 | -1.590 | -0.701 | <1x10-14 |
| *EPHX2* | 0.236 | -1.823 | -0.701 | <1x10-14 |
| *SSBP2* | 0.342 | -1.261 | -0.699 | <1x10-14 |
| *CAB39L* | 0.300 | -2.395 | -0.699 | <1x10-14 |
| *TRPM2* | -0.235 | 2.058 | -0.697 | <1x10-14 |
| *PPARG* | 0.263 | -2.459 | -0.696 | <1x10-14 |
| *GPR153* | -0.241 | 1.814 | -0.694 | <1x10-14 |
| *CDON* | 0.260 | -1.792 | -0.693 | <1x10-14 |
| *C3orf18* | 0.250 | -2.163 | -0.692 | <1x10-14 |
| *SERTAD4* | 0.291 | -1.330 | -0.689 | <1x10-14 |
| *CD80* | -0.253 | 2.929 | -0.687 | <1x10-14 |
| *MFAP2* | -0.220 | 3.020 | -0.687 | <1x10-14 |
| *DOPEY2* | 0.331 | -1.139 | -0.686 | <1x10-14 |
| *SDPR* | 0.236 | -1.945 | -0.675 | <1x10-14 |
| *MAP1LC3A* | 0.216 | -1.809 | -0.675 | <1x10-14 |
| *ZNF583* | 0.349 | -1.235 | -0.672 | <1x10-14 |
| *APBA2* | -0.248 | 2.443 | -0.671 | <1x10-14 |
| *OSBPL1A* | 0.313 | -1.053 | -0.671 | 1.31x10-12 |
| *MYH14* | 0.297 | -2.574 | -0.670 | <1x10-14 |
| *ALS2CR11* | 0.284 | -1.491 | -0.668 | <1x10-14 |
| *PITX1* | 0.233 | -1.382 | -0.668 | <1x10-14 |
| *EYA2* | 0.214 | -2.186 | -0.667 | <1x10-14 |
| *NUPR1* | 0.248 | -1.215 | -0.667 | <1x10-14 |
| *THBS4* | 0.379 | -1.490 | -0.665 | <1x10-14 |
| *PODN* | 0.286 | -2.329 | -0.665 | <1x10-14 |
| *SYNGR1* | 0.259 | -2.320 | -0.665 | <1x10-14 |
| *CPAMD8* | 0.233 | -3.285 | -0.664 | <1x10-14 |
| *PIK3CD* | -0.261 | 1.272 | -0.660 | <1x10-14 |
| *CKMT2* | 0.205 | -4.227 | -0.659 | <1x10-14 |
| *LILRB4* | -0.231 | 1.644 | -0.657 | <1x10-14 |
| *ZNF853* | 0.271 | -2.060 | -0.654 | <1x10-14 |
| *MAGI2* | 0.294 | -1.843 | -0.653 | <1x10-14 |
| *CD274* | -0.241 | 2.056 | -0.649 | <1x10-14 |
| *PLEKHG6* | 0.223 | -2.390 | -0.648 | <1x10-14 |
| *SIGLEC10* | -0.236 | 1.421 | -0.647 | <1x10-14 |
| *AOX1* | 0.215 | -2.724 | -0.647 | <1x10-14 |
| *AKAP7* | 0.241 | -1.277 | -0.645 | <1x10-14 |
| *GAS7* | 0.210 | -1.439 | -0.642 | <1x10-14 |
| *ADAM33* | 0.236 | -2.073 | -0.638 | <1x10-14 |
| *L3MBTL4* | 0.259 | -2.066 | -0.636 | <1x10-14 |
| *HAVCR2* | -0.280 | 1.514 | -0.630 | <1x10-14 |
| *ZBTB16* | 0.243 | -4.036 | -0.630 | 7.49x10-11 |
| *ADAMTSL1* | 0.260 | -1.129 | -0.624 | <1x10-14 |
| *ALG1L* | -0.316 | 1.986 | -0.622 | <1x10-14 |
| *CCR7* | -0.224 | 1.463 | -0.622 | <1x10-14 |
| *EFHA2* | 0.215 | -2.101 | -0.621 | <1x10-14 |
| *RANBP17* | 0.256 | -2.461 | -0.615 | <1x10-14 |
| *OLR1* | -0.227 | 2.398 | -0.610 | <1x10-14 |
| *PER2* | 0.281 | -1.123 | -0.610 | <1x10-14 |
| *LCK* | -0.277 | 1.175 | -0.604 | 1.54x10-10 |
| *FAM180A* | 0.285 | -1.451 | -0.603 | 2.63x10-10 |
| *ZC2HC1C* | 0.248 | -1.316 | -0.601 | 5.62x10-10 |
| *P2RY6* | -0.213 | 1.816 | -0.600 | 6.30x10-10 |
| *TCP11L2* | 0.241 | -1.399 | -0.597 | 1.23x10-9 |
| *SSPN* | 0.236 | -1.671 | -0.596 | 1.40x10-9 |
| *ANO5* | 0.260 | -3.724 | -0.592 | 2.38x10-9 |
| *SOX17* | 0.279 | -1.341 | -0.592 | 2.46x10-9 |
| *ZBED3* | 0.211 | -1.535 | -0.591 | 2.55x10-9 |
| *CD34* | 0.226 | -1.192 | -0.587 | 2.58x10-9 |
| *SVIL* | 0.238 | -1.663 | -0.586 | 4.41x10-9 |
| *FAM47E* | 0.271 | -2.382 | -0.579 | 0.8x10-9 |
| *CD72* | -0.273 | 1.645 | -0.575 | 1.16x10-8 |
| *PLAGL1* | 0.201 | -2.005 | -0.570 | 1.69x10-8 |
| *AIM2* | -0.361 | 1.802 | -0.569 | 1.76x10-8 |
| *EBF1* | 0.276 | -1.143 | -0.567 | 2.12x10-8 |
| *WNK4* | 0.302 | -3.841 | -0.559 | 3.53x10-8 |
| *IFITM1* | -0.202 | 1.361 | -0.556 | 4.31x10-8 |
| *AK8* | 0.272 | -1.285 | -0.556 | 4.31x10-8 |
| *PREX2* | 0.245 | -1.233 | -0.554 | 4.93x10-8 |
| *PI3* | -0.217 | 1.124 | -0.554 | 5.15x10-8 |
| *GLIS1* | -0.225 | 1.744 | -0.538 | 1.37x10-7 |
| *RWDD3* | 0.267 | -1.030 | -0.537 | 1.49x10-7 |
| *NOSTRIN* | 0.201 | -2.279 | -0.534 | 1.82x10-7 |
| *ADAM32* | 0.247 | -1.554 | -0.530 | 2.29x10-7 |
| *RAI14* | -0.259 | 1.267 | -0.528 | 2.61x10-7 |
| *AKAP6* | 0.222 | -1.340 | -0.527 | 2.71x10-7 |
| *KIF5C* | 0.281 | -1.331 | -0.517 | 4.91x10-7 |
| *CPEB1* | 0.201 | -2.541 | -0.512 | 6.0x10-8 |
| *LILRB1* | -0.233 | 1.775 | -0.511 | 6.84x10-7 |
| *C20orf197* | -0.234 | 2.621 | -0.508 | 8.13x10-7 |
| *SLAIN1* | 0.233 | -1.735 | -0.499 | 1.33x10-6 |
| *ABCA4* | -0.274 | 2.810 | -0.496 | 1.55x10-6 |
| *FAM171B* | 0.202 | -1.357 | -0.492 | 1.93x10-6 |
| *C14orf39* | 0.352 | -2.282 | -0.489 | 2.21x10-6 |
| *THSD4* | 0.304 | -1.010 | -0.485 | 2.77x10-6 |
| *PMEPA1* | -0.249 | 1.535 | -0.483 | 3.04x10-6 |
| *TET1* | 0.227 | -1.948 | -0.477 | 4.16x10-6 |
| *FGF12* | 0.240 | -1.773 | -0.473 | 5.10x10-6 |
| *CYP27A1* | 0.244 | -2.186 | -0.473 | 5.21x10-6 |
| *SVEP1* | 0.269 | -1.182 | -0.469 | 6.12x10-6 |
| *ST6GALNAC3* | 0.250 | -1.155 | -0.462 | 0.8x10-7 |
| *TMEM178B* | 0.269 | -2.735 | -0.460 | 9.91x10-6 |
| *RNF180* | 0.213 | -2.240 | -0.457 | 1.12x10-5 |
| *TBX15* | 0.326 | -2.905 | -0.456 | 1.15x10-5 |
| *SLC14A1* | 0.263 | -2.228 | -0.453 | 1.33x10-5 |
| *C1QB* | -0.233 | 1.195 | -0.453 | 1.37x10-5 |
| *RYR2* | 0.302 | -2.764 | -0.450 | 1.56x10-5 |
| *CCL24* | -0.211 | 2.003 | -0.443 | 2.18x10-5 |
| *FAM19A5* | 0.237 | -1.037 | -0.440 | 2.43x10-5 |
| *COL8A1* | -0.211 | 2.141 | -0.438 | 2.64x10-5 |
| *CXCL12* | 0.298 | -1.543 | -0.428 | 4.16x10-5 |
| *SUSD4* | 0.300 | -1.921 | -0.426 | 4.52x10-5 |
| *PGM1* | 0.337 | -1.558 | -0.418 | 6.62x10-5 |
| *SEMA6C* | 0.227 | -3.088 | -0.417 | 6.85x10-5 |
| *WBSCR17* | 0.249 | -1.266 | -0.411 | 8.86x10-5 |
| *LAIR1* | -0.235 | 1.221 | -0.389 | 2x10-4 |
| *SNTB1* | 0.246 | -1.171 | -0.378 | 3x10-4 |
| *SULF1* | -0.230 | 2.379 | -0.376 | 4x10-4 |
| *CD36* | 0.262 | -1.382 | -0.371 | 4x10-4 |
| *ROBO3* | 0.226 | -1.019 | -0.362 | 6x10-4 |
| *SLC25A25* | 0.261 | -1.178 | -0.358 | 7x10-4 |
| *SLC19A3* | 0.255 | -1.427 | -0.343 | 0.001 |
| *ZBP1* | -0.234 | 1.903 | -0.343 | 0.001 |
| *DYRK1B* | 0.288 | -2.188 | -0.296 | 0.005 |
| *SMIM5* | 0.208 | -1.636 | -0.295 | 0.006 |
| *DOK5* | 0.312 | -1.737 | -0.258 | 0.016 |
| *TMEM63A* | 0.298 | -1.416 | -0.243 | 0.023 |
