# Supplementary material for: Epigenomic dysregulation-mediated alterations of key biological pathways and tumor immune evasion are hallmarks of gingivo-buccal oral cancer
Source: Clin Epigenetics. 2019 Dec 3;11:178. doi: 10.1186/s13148-019-0782-2 (PMC6889354; doi:10.1186/s13148-019-0782-2)
Supplement: Supplementary file 2 — Additional file 2: Table S2. Information related to alteration of expression of 209 epigenetically dysregulated genes by one or more known drugs. [file 13148_2019_782_MOESM2_ESM.docx]

| **Supplementary Table S2. Information related to alteration of expression of 209 epigenetically dysregulated genes by one or more known drugs** | | | | | |
| --- | --- | --- | --- | --- | --- |
| **(A) Drugs that Increase Expression** | | | | | |
| **DSigDB database** | | | | **Present Study** | |
| **Drug** | **Gene** | **Type** | **Source** | **Mean_delta_beta** | **Mean_log2(Fold Change)** |
| docetaxel | ABCA4 | increases expression | Comparative Toxicogenomics Database | NA | NA |
| vincristine | ABCA4 | increases expression | Comparative Toxicogenomics Database | NA | NA |
| calcitriol | ACSS3 | increases expression | Comparative Toxicogenomics Database | 0.259781859 | -2.77663414 |
| testosterone | ACSS3 | increases expression | Comparative Toxicogenomics Database | 0.259781859 | -2.77663414 |
| SELENIUM | ADCY6 | increases expression | Comparative Toxicogenomics Database | 0.311331346 | -1.391846896 |
| Melitten | AIM2 | increases expression | Comparative Toxicogenomics Database | NA | NA |
| Bortezomib | AIM2 | increases expression | Comparative Toxicogenomics Database | NA | NA |
| formaldehyde | AIM2 | increases expression | Comparative Toxicogenomics Database | NA | NA |
| Demecolcine | AIM2 | increases expression | Comparative Toxicogenomics Database | NA | NA |
| vincristine | AIM2 | increases expression | Comparative Toxicogenomics Database | NA | NA |
| curcumin | AKAP6 | increases expression | Comparative Toxicogenomics Database | 0.222037246 | -1.339893639 |
| acetaminophen | AKAP7 | increases expression | Comparative Toxicogenomics Database | 0.240960699 | -1.2772238 |
| dexamethasone | AKAP7 | increases expression | Comparative Toxicogenomics Database | 0.240960699 | -1.2772238 |
| IBMX | AKAP7 | increases expression | Comparative Toxicogenomics Database | 0.240960699 | -1.2772238 |
| quercetin | AMPD3 | increases expression | Comparative Toxicogenomics Database | NA | NA |
| thiram | AMPD3 | increases expression | Comparative Toxicogenomics Database | NA | NA |
| acetaminophen | AMPD3 | increases expression | Comparative Toxicogenomics Database | NA | NA |
| atrazine | AMPD3 | increases expression | Comparative Toxicogenomics Database | NA | NA |
| ACROLEIN | AMPD3 | increases expression | Comparative Toxicogenomics Database | NA | NA |
| EPICHLOROHYDRIN | AMPD3 | increases expression | Comparative Toxicogenomics Database | NA | NA |
| Andriol | AMPD3 | increases expression | Comparative Toxicogenomics Database | NA | NA |
| 1,6-Diisocyanatohexane | AMPD3 | increases expression | Comparative Toxicogenomics Database | NA | NA |
| benzo[a]pyrene | AMPD3 | increases expression | Comparative Toxicogenomics Database | NA | NA |
| cinnamaldehyde | AMPD3 | increases expression | Comparative Toxicogenomics Database | NA | NA |
| CHLOROPICRIN | AMPD3 | increases expression | Comparative Toxicogenomics Database | NA | NA |
| 2-Mercaptobenzothiazole | AMPD3 | increases expression | Comparative Toxicogenomics Database | NA | NA |
| Tetradioxin | AMPD3 | increases expression | Comparative Toxicogenomics Database | NA | NA |
| 7-ACA | AMPD3 | increases expression | Comparative Toxicogenomics Database | NA | NA |
| Silica | ANO5 | increases expression | Comparative Toxicogenomics Database | 0.260390061 | -3.724404434 |
| Mehp | AOX1 | increases expression | Comparative Toxicogenomics Database | 0.215335057 | -2.723687747 |
| progesterone | AOX1 | increases expression | Comparative Toxicogenomics Database | 0.215335057 | -2.723687747 |
| estradiol | AOX1 | increases expression | Comparative Toxicogenomics Database | 0.215335057 | -2.723687747 |
| COPPER | AOX1 | increases expression | Comparative Toxicogenomics Database | 0.215335057 | -2.723687747 |
| benzo[a]pyrene | AOX1 | increases expression | Comparative Toxicogenomics Database | 0.215335057 | -2.723687747 |
| Tetradioxin | AOX1 | increases expression | Comparative Toxicogenomics Database | 0.215335057 | -2.723687747 |
| CADMIUM | BST2 | increases expression | Comparative Toxicogenomics Database | NA | NA |
| tamoxifen | BST2 | increases expression | Comparative Toxicogenomics Database | NA | NA |
| SELENIUM | BST2 | increases expression | Comparative Toxicogenomics Database | NA | NA |
| estradiol | BST2 | increases expression | Comparative Toxicogenomics Database | NA | NA |
| 3'-Azido-3'-deoxythymidine | BST2 | increases expression | Comparative Toxicogenomics Database | NA | NA |
| Tetradioxin | BST2 | increases expression | Comparative Toxicogenomics Database | NA | NA |
| ZINC | C1QB | increases expression | Comparative Toxicogenomics Database | NA | NA |
| nimesulide | C1QB | increases expression | Comparative Toxicogenomics Database | NA | NA |
| SELENIUM | C1QB | increases expression | Comparative Toxicogenomics Database | NA | NA |
| Ephedrone | C1QB | increases expression | Comparative Toxicogenomics Database | NA | NA |
| testosterone | CAB39L | increases expression | Comparative Toxicogenomics Database | 0.299863806 | -2.395059274 |
| PHENCYCLIDINE | CCL24 | increases expression | Comparative Toxicogenomics Database | NA | NA |
| ZINC | CCL24 | increases expression | Comparative Toxicogenomics Database | NA | NA |
| quercetin | CCR7 | increases expression | Comparative Toxicogenomics Database | NA | NA |
| CADMIUM | CCR7 | increases expression | Comparative Toxicogenomics Database | NA | NA |
| Oxazolone | CCR7 | increases expression | Comparative Toxicogenomics Database | NA | NA |
| Tributyltin | CCR7 | increases expression | Comparative Toxicogenomics Database | NA | NA |
| formaldehyde | CCR7 | increases expression | Comparative Toxicogenomics Database | NA | NA |
| eugenol | CCR7 | increases expression | Comparative Toxicogenomics Database | NA | NA |
| Hydroxycitronellal | CCR7 | increases expression | Comparative Toxicogenomics Database | NA | NA |
| Alitretinoin | CCR7 | increases expression | Comparative Toxicogenomics Database | NA | NA |
| 15-Acetyldeoxynivalenol | CCR7 | increases expression | Comparative Toxicogenomics Database | NA | NA |
| benzo[a]pyrene | CCR7 | increases expression | Comparative Toxicogenomics Database | NA | NA |
| Rifampicin | CCR7 | increases expression | Comparative Toxicogenomics Database | NA | NA |
| Dinoprostone | CCR7 | increases expression | Comparative Toxicogenomics Database | NA | NA |
| Tetradioxin | CCR7 | increases expression | Comparative Toxicogenomics Database | NA | NA |
| 1-chloro-2,4-dinitrobenzene | CCR7 | increases expression | Comparative Toxicogenomics Database | NA | NA |
| Vitinoin | CD34 | increases expression | Comparative Toxicogenomics Database | 0.225531203 | -1.192041899 |
| arsenite | CD34 | increases expression | Comparative Toxicogenomics Database | 0.225531203 | -1.192041899 |
| Synercid | CD34 | increases expression | Comparative Toxicogenomics Database | 0.225531203 | -1.192041899 |
| erythromycin | CD34 | increases expression | Comparative Toxicogenomics Database | 0.225531203 | -1.192041899 |
| bezafibrate | CD36 | increases expression | Comparative Toxicogenomics Database | 0.261681464 | -1.381548096 |
| wortmannin | CD36 | increases expression | Comparative Toxicogenomics Database | 0.261681464 | -1.381548096 |
| losartan | CD36 | increases expression | Comparative Toxicogenomics Database | 0.261681464 | -1.381548096 |
| candesartan | CD36 | increases expression | Comparative Toxicogenomics Database | 0.261681464 | -1.381548096 |
| ibuprofen | CD36 | increases expression | Comparative Toxicogenomics Database | 0.261681464 | -1.381548096 |
| Malondialdehyde | CD36 | increases expression | Comparative Toxicogenomics Database | 0.261681464 | -1.381548096 |
| Anandamide | CD36 | increases expression | Comparative Toxicogenomics Database | 0.261681464 | -1.381548096 |
| ACMC-20mvek | CD36 | increases expression | Comparative Toxicogenomics Database | 0.261681464 | -1.381548096 |
| oxozinc | CD36 | increases expression | Comparative Toxicogenomics Database | 0.261681464 | -1.381548096 |
| Pioglitazone | CD36 | increases expression | Comparative Toxicogenomics Database | 0.261681464 | -1.381548096 |
| GW9662 | CD36 | increases expression | Comparative Toxicogenomics Database | 0.261681464 | -1.381548096 |
| Myxothiazol | CD36 | increases expression | Comparative Toxicogenomics Database | 0.261681464 | -1.381548096 |
| yc-1 | CD36 | increases expression | Comparative Toxicogenomics Database | 0.261681464 | -1.381548096 |
| tamoxifen | CD36 | increases expression | Comparative Toxicogenomics Database | 0.261681464 | -1.381548096 |
| dexamethasone | CD36 | increases expression | Comparative Toxicogenomics Database | 0.261681464 | -1.381548096 |
| estradiol | CD36 | increases expression | Comparative Toxicogenomics Database | 0.261681464 | -1.381548096 |
| oxygen | CD36 | increases expression | Comparative Toxicogenomics Database | 0.261681464 | -1.381548096 |
| Telmisartan | CD36 | increases expression | Comparative Toxicogenomics Database | 0.261681464 | -1.381548096 |
| Netoglitazone | CD36 | increases expression | Comparative Toxicogenomics Database | 0.261681464 | -1.381548096 |
| rapamycin | CD36 | increases expression | Comparative Toxicogenomics Database | 0.261681464 | -1.381548096 |
| Rifaximin | CD36 | increases expression | Comparative Toxicogenomics Database | 0.261681464 | -1.381548096 |
| resveratrol | CD36 | increases expression | Comparative Toxicogenomics Database | 0.261681464 | -1.381548096 |
| Alitretinoin | CD36 | increases expression | Comparative Toxicogenomics Database | 0.261681464 | -1.381548096 |
| Arvanil | CD36 | increases expression | Comparative Toxicogenomics Database | 0.261681464 | -1.381548096 |
| benzo[a]pyrene | CD36 | increases expression | Comparative Toxicogenomics Database | 0.261681464 | -1.381548096 |
| Acetovanillone | CD36 | increases expression | Comparative Toxicogenomics Database | 0.261681464 | -1.381548096 |
| troglitazone | CD36 | increases expression | Comparative Toxicogenomics Database | 0.261681464 | -1.381548096 |
| Tetradioxin | CD36 | increases expression | Comparative Toxicogenomics Database | 0.261681464 | -1.381548096 |
| rosiglitazone | CD36 | increases expression | Comparative Toxicogenomics Database | 0.261681464 | -1.381548096 |
| Electrocorundum | CD36 | increases expression | Comparative Toxicogenomics Database | 0.261681464 | -1.381548096 |
| Tamibarotene | CD36 | increases expression | Comparative Toxicogenomics Database | 0.261681464 | -1.381548096 |
| atrazine | CD72 | increases expression | Comparative Toxicogenomics Database | NA | NA |
| HELENALIN | CD80 | increases expression | Comparative Toxicogenomics Database | NA | NA |
| PEPCID | CD80 | increases expression | Comparative Toxicogenomics Database | NA | NA |
| histamine | CD80 | increases expression | Comparative Toxicogenomics Database | NA | NA |
| carmustine | CD80 | increases expression | Comparative Toxicogenomics Database | NA | NA |
| formaldehyde | CD80 | increases expression | Comparative Toxicogenomics Database | NA | NA |
| Demecolcine | CD80 | increases expression | Comparative Toxicogenomics Database | NA | NA |
| 4-Methylhistamine | CD80 | increases expression | Comparative Toxicogenomics Database | NA | NA |
| vincristine | CD80 | increases expression | Comparative Toxicogenomics Database | NA | NA |
| Decitabine | CD80 | increases expression | Comparative Toxicogenomics Database | NA | NA |
| Lupane | CD80 | increases expression | Comparative Toxicogenomics Database | NA | NA |
| Chromium(VI) | CD80 | increases expression | Comparative Toxicogenomics Database | NA | NA |
| Dimaprit | CD80 | increases expression | Comparative Toxicogenomics Database | NA | NA |
| Hydroxycitronellal | CD80 | increases expression | Comparative Toxicogenomics Database | NA | NA |
| FK-565 | CD80 | increases expression | Comparative Toxicogenomics Database | NA | NA |
| ranitidine | CD80 | increases expression | Comparative Toxicogenomics Database | NA | NA |
| Tetradioxin | CD80 | increases expression | Comparative Toxicogenomics Database | NA | NA |
| etoposide | CD274 | increases expression | Comparative Toxicogenomics Database | NA | NA |
| p-Phenylenediamine | CD274 | increases expression | Comparative Toxicogenomics Database | NA | NA |
| CHEBI:18224 | CD274 | increases expression | Comparative Toxicogenomics Database | NA | NA |
| paclitaxel | CD274 | increases expression | Comparative Toxicogenomics Database | NA | NA |
| 5-Fluorouracil | CD274 | increases expression | Comparative Toxicogenomics Database | NA | NA |
| calcitriol | CD274 | increases expression | Comparative Toxicogenomics Database | NA | NA |
| formaldehyde | CD274 | increases expression | Comparative Toxicogenomics Database | NA | NA |
| Demecolcine | CD274 | increases expression | Comparative Toxicogenomics Database | NA | NA |
| aspirin | CD274 | increases expression | Comparative Toxicogenomics Database | NA | NA |
| vincristine | CD274 | increases expression | Comparative Toxicogenomics Database | NA | NA |
| oxygen | CD274 | increases expression | Comparative Toxicogenomics Database | NA | NA |
| UNII-9XX54M675G | CD274 | increases expression | Comparative Toxicogenomics Database | NA | NA |
| cinnamaldehyde | CD274 | increases expression | Comparative Toxicogenomics Database | NA | NA |
| 1-chloro-2,4-dinitrobenzene | CD274 | increases expression | Comparative Toxicogenomics Database | NA | NA |
| testosterone | CD274 | increases expression | Comparative Toxicogenomics Database | NA | NA |
| atrazine | CGNL1 | increases expression | Comparative Toxicogenomics Database | 0.271051143 | -3.348826746 |
| AFLATOXIN_B1 | CHADL | increases expression | Comparative Toxicogenomics Database | 0.227070649 | -2.191154363 |
| progesterone | CHPT1 | increases expression | Comparative Toxicogenomics Database | 0.322213369 | -2.435181016 |
| Enterolactone | CHPT1 | increases expression | Comparative Toxicogenomics Database | 0.322213369 | -2.435181016 |
| atrazine | CHPT1 | increases expression | Comparative Toxicogenomics Database | 0.322213369 | -2.435181016 |
| 67526-95-8 | CHPT1 | increases expression | Comparative Toxicogenomics Database | 0.322213369 | -2.435181016 |
| estradiol | CHPT1 | increases expression | Comparative Toxicogenomics Database | 0.322213369 | -2.435181016 |
| COUMESTROL | CHPT1 | increases expression | Comparative Toxicogenomics Database | 0.322213369 | -2.435181016 |
| benzo[a]pyrene | CKMT2 | increases expression | Comparative Toxicogenomics Database | 0.205272086 | -4.227423583 |
| Tetradioxin | CKMT2 | increases expression | Comparative Toxicogenomics Database | 0.205272086 | -4.227423583 |
| Tetradioxin | CLDN11 | increases expression | Comparative Toxicogenomics Database | 0.245240041 | -2.50389644 |
| atrazine | CPEB1 | increases expression | Comparative Toxicogenomics Database | 0.200711917 | -2.541135447 |
| CADMIUM | CXCL12 | increases expression | Comparative Toxicogenomics Database | 0.297955213 | -1.542652747 |
| Hydroxychlor | CXCL12 | increases expression | Comparative Toxicogenomics Database | 0.297955213 | -1.542652747 |
| 1-BROMOPROPANE | CXCL12 | increases expression | Comparative Toxicogenomics Database | 0.297955213 | -1.542652747 |
| daidzein | CXCL12 | increases expression | Comparative Toxicogenomics Database | 0.297955213 | -1.542652747 |
| apigenin | CXCL12 | increases expression | Comparative Toxicogenomics Database | 0.297955213 | -1.542652747 |
| p-benzoquinone | CXCL12 | increases expression | Comparative Toxicogenomics Database | 0.297955213 | -1.542652747 |
| Vitinoin | CXCL12 | increases expression | Comparative Toxicogenomics Database | 0.297955213 | -1.542652747 |
| curcumin | CXCL12 | increases expression | Comparative Toxicogenomics Database | 0.297955213 | -1.542652747 |
| Apocarotenal | CXCL12 | increases expression | Comparative Toxicogenomics Database | 0.297955213 | -1.542652747 |
| Decitabine | CXCL12 | increases expression | Comparative Toxicogenomics Database | 0.297955213 | -1.542652747 |
| 27-hydroxycholesterol | CXCL12 | increases expression | Comparative Toxicogenomics Database | 0.297955213 | -1.542652747 |
| 5-azacytidine | CXCL12 | increases expression | Comparative Toxicogenomics Database | 0.297955213 | -1.542652747 |
| tamoxifen | CXCL12 | increases expression | Comparative Toxicogenomics Database | 0.297955213 | -1.542652747 |
| Premarin | CXCL12 | increases expression | Comparative Toxicogenomics Database | 0.297955213 | -1.542652747 |
| 4-Hydroxytamoxifen | CXCL12 | increases expression | Comparative Toxicogenomics Database | 0.297955213 | -1.542652747 |
| genistein | CXCL12 | increases expression | Comparative Toxicogenomics Database | 0.297955213 | -1.542652747 |
| estradiol | CXCL12 | increases expression | Comparative Toxicogenomics Database | 0.297955213 | -1.542652747 |
| mifepristone | CXCL12 | increases expression | Comparative Toxicogenomics Database | 0.297955213 | -1.542652747 |
| Fulvestrant | CXCL12 | increases expression | Comparative Toxicogenomics Database | 0.297955213 | -1.542652747 |
| resveratrol | CXCL12 | increases expression | Comparative Toxicogenomics Database | 0.297955213 | -1.542652747 |
| raloxifene | CXCL12 | increases expression | Comparative Toxicogenomics Database | 0.297955213 | -1.542652747 |
| methotrexate | CXCL12 | increases expression | Comparative Toxicogenomics Database | 0.297955213 | -1.542652747 |
| COUMESTROL | CXCL12 | increases expression | Comparative Toxicogenomics Database | 0.297955213 | -1.542652747 |
| Glyceollin | CXCL12 | increases expression | Comparative Toxicogenomics Database | 0.297955213 | -1.542652747 |
| beta-carotene | CXCL12 | increases expression | Comparative Toxicogenomics Database | 0.297955213 | -1.542652747 |
| Benz[a]anthracene | CXCL12 | increases expression | Comparative Toxicogenomics Database | 0.297955213 | -1.542652747 |
| Tetradioxin | CXCL12 | increases expression | Comparative Toxicogenomics Database | 0.297955213 | -1.542652747 |
| Enterolactone | CYP2R1 | increases expression | Comparative Toxicogenomics Database | 0.283118343 | -1.17055342 |
| amiodarone | CYP2R1 | increases expression | Comparative Toxicogenomics Database | 0.283118343 | -1.17055342 |
| COUMESTROL | CYP2R1 | increases expression | Comparative Toxicogenomics Database | 0.283118343 | -1.17055342 |
| atrazine | CYP27A1 | increases expression | Comparative Toxicogenomics Database | 0.243604261 | -2.185802793 |
| 2-arachidonoylglycerol | CYP27A1 | increases expression | Comparative Toxicogenomics Database | 0.243604261 | -2.185802793 |
| GW9662 | CYP27A1 | increases expression | Comparative Toxicogenomics Database | 0.243604261 | -2.185802793 |
| 27-hydroxycholesterol | CYP27A1 | increases expression | Comparative Toxicogenomics Database | 0.243604261 | -2.185802793 |
| dexamethasone | CYP27A1 | increases expression | Comparative Toxicogenomics Database | 0.243604261 | -2.185802793 |
| 1,2,4-BENZENETRIOL | CYP27A1 | increases expression | Comparative Toxicogenomics Database | 0.243604261 | -2.185802793 |
| resveratrol | CYP27A1 | increases expression | Comparative Toxicogenomics Database | 0.243604261 | -2.185802793 |
| Alitretinoin | CYP27A1 | increases expression | Comparative Toxicogenomics Database | 0.243604261 | -2.185802793 |
| Rifampicin | CYP27A1 | increases expression | Comparative Toxicogenomics Database | 0.243604261 | -2.185802793 |
| Tetradioxin | CYP27A1 | increases expression | Comparative Toxicogenomics Database | 0.243604261 | -2.185802793 |
| acetaminophen | DLGAP4 | increases expression | Comparative Toxicogenomics Database | NA | NA |
| atrazine | DLGAP4 | increases expression | Comparative Toxicogenomics Database | NA | NA |
| formaldehyde | DLGAP4 | increases expression | Comparative Toxicogenomics Database | NA | NA |
| LEAD | DLGAP4 | increases expression | Comparative Toxicogenomics Database | NA | NA |
| arbutin | DLGAP4 | increases expression | Comparative Toxicogenomics Database | NA | NA |
| 7646-79-9 | DLGAP4 | increases expression | Comparative Toxicogenomics Database | NA | NA |
| deferoxamine | DNMT3B | increases expression | Comparative Toxicogenomics Database | NA | NA |
| acetaminophen | DNMT3B | increases expression | Comparative Toxicogenomics Database | NA | NA |
| Cianidanol | DNMT3B | increases expression | Comparative Toxicogenomics Database | NA | NA |
| Decitabine | DNMT3B | increases expression | Comparative Toxicogenomics Database | NA | NA |
| tamoxifen | DNMT3B | increases expression | Comparative Toxicogenomics Database | NA | NA |
| Silica | DOK5 | increases expression | Comparative Toxicogenomics Database | 0.311606778 | -1.737018877 |
| Andriol | DOK5 | increases expression | Comparative Toxicogenomics Database | 0.311606778 | -1.737018877 |
| norgestrel | DOK5 | increases expression | Comparative Toxicogenomics Database | 0.311606778 | -1.737018877 |
| acetaminophen | DOPEY2 | increases expression | Comparative Toxicogenomics Database | 0.331108652 | -1.138603699 |
| quercetin | DSE | increases expression | Comparative Toxicogenomics Database | NA | NA |
| acetaminophen | DSE | increases expression | Comparative Toxicogenomics Database | NA | NA |
| formaldehyde | DSE | increases expression | Comparative Toxicogenomics Database | NA | NA |
| 8-HYDROXYQUINOLINE | DSE | increases expression | Comparative Toxicogenomics Database | NA | NA |
| Caspan | EBF1 | increases expression | Comparative Toxicogenomics Database | 0.27614783 | -1.142523321 |
| EXEMESTANE | EBF1 | increases expression | Comparative Toxicogenomics Database | 0.27614783 | -1.142523321 |
| vincristine | EBF1 | increases expression | Comparative Toxicogenomics Database | 0.27614783 | -1.142523321 |
| 5,6-BENZOFLAVONE | EPHX1 | increases expression | Comparative Toxicogenomics Database | 0.289636703 | -1.236087583 |
| Dasatinib | EPHX1 | increases expression | Comparative Toxicogenomics Database | 0.289636703 | -1.236087583 |
| phenobarbital | EPHX1 | increases expression | Comparative Toxicogenomics Database | 0.289636703 | -1.236087583 |
| Glycidamide | EPHX1 | increases expression | Comparative Toxicogenomics Database | 0.289636703 | -1.236087583 |
| acetaminophen | EPHX1 | increases expression | Comparative Toxicogenomics Database | 0.289636703 | -1.236087583 |
| 5-Fluorouracil | EPHX1 | increases expression | Comparative Toxicogenomics Database | 0.289636703 | -1.236087583 |
| carbamazepine | EPHX1 | increases expression | Comparative Toxicogenomics Database | 0.289636703 | -1.236087583 |
| atrazine | EPHX1 | increases expression | Comparative Toxicogenomics Database | 0.289636703 | -1.236087583 |
| Decitabine | EPHX1 | increases expression | Comparative Toxicogenomics Database | 0.289636703 | -1.236087583 |
| genistein | EPHX1 | increases expression | Comparative Toxicogenomics Database | 0.289636703 | -1.236087583 |
| SILVER | EPHX1 | increases expression | Comparative Toxicogenomics Database | 0.289636703 | -1.236087583 |
| resveratrol | EPHX1 | increases expression | Comparative Toxicogenomics Database | 0.289636703 | -1.236087583 |
| benzo[a]pyrene | EPHX1 | increases expression | Comparative Toxicogenomics Database | 0.289636703 | -1.236087583 |
| Rifampicin | EPHX1 | increases expression | Comparative Toxicogenomics Database | 0.289636703 | -1.236087583 |
| doxorubicin | EPHX1 | increases expression | Comparative Toxicogenomics Database | 0.289636703 | -1.236087583 |
| 7646-79-9 | EPHX1 | increases expression | Comparative Toxicogenomics Database | 0.289636703 | -1.236087583 |
| naphthalene | EPHX1 | increases expression | Comparative Toxicogenomics Database | 0.289636703 | -1.236087583 |
| (17S)-17-hydroxy-13,17-dimethyl-1,2,6,7,8,14,15,16-octahydrocyclopenta[a]phenanthren-3-one | EPHX2 | increases expression | Comparative Toxicogenomics Database | 0.235803509 | -1.82307751 |
| SELENIUM | EPHX2 | increases expression | Comparative Toxicogenomics Database | 0.235803509 | -1.82307751 |
| genistein | EPHX2 | increases expression | Comparative Toxicogenomics Database | 0.235803509 | -1.82307751 |
| troglitazone | EPHX2 | increases expression | Comparative Toxicogenomics Database | 0.235803509 | -1.82307751 |
| naphthalene | EPHX2 | increases expression | Comparative Toxicogenomics Database | 0.235803509 | -1.82307751 |
| 2,6-DICHLOROINDOPHENOL | EPHX2 | increases expression | Comparative Toxicogenomics Database | 0.235803509 | -1.82307751 |
| SELENIUM | EYA2 | increases expression | Comparative Toxicogenomics Database | 0.213923327 | -2.185606174 |
| Silica | FAM19A5 | increases expression | Comparative Toxicogenomics Database | 0.23734584 | -1.037250656 |
| formaldehyde | FAM63A | increases expression | Comparative Toxicogenomics Database | 0.325519333 | -1.19048283 |
| methotrexate | FAM63A | increases expression | Comparative Toxicogenomics Database | 0.325519333 | -1.19048283 |
| progesterone | FAM171B | increases expression | Comparative Toxicogenomics Database | 0.201717098 | -1.356632417 |
| estradiol | FAM171B | increases expression | Comparative Toxicogenomics Database | 0.201717098 | -1.356632417 |
| raloxifene | FAM171B | increases expression | Comparative Toxicogenomics Database | 0.201717098 | -1.356632417 |
| theophylline | FAM171B | increases expression | Comparative Toxicogenomics Database | 0.201717098 | -1.356632417 |
| atrazine | FAM180A | increases expression | Comparative Toxicogenomics Database | 0.284839063 | -1.451361929 |
| SELENIUM | FBXL8 | increases expression | Comparative Toxicogenomics Database | 0.202297876 | -1.061375701 |
| benzo[a]pyrene | FBXL8 | increases expression | Comparative Toxicogenomics Database | 0.202297876 | -1.061375701 |
| IRON | GAS7 | increases expression | Comparative Toxicogenomics Database | 0.210489649 | -1.438986603 |
| MERCURY | GAS7 | increases expression | Comparative Toxicogenomics Database | 0.210489649 | -1.438986603 |
| Tetradioxin | GAS7 | increases expression | Comparative Toxicogenomics Database | 0.210489649 | -1.438986603 |
| Enterolactone | GFRA1 | increases expression | Comparative Toxicogenomics Database | 0.294041254 | -3.020982203 |
| genistein | GFRA1 | increases expression | Comparative Toxicogenomics Database | 0.294041254 | -3.020982203 |
| estradiol | GFRA1 | increases expression | Comparative Toxicogenomics Database | 0.294041254 | -3.020982203 |
| resveratrol | GFRA1 | increases expression | Comparative Toxicogenomics Database | 0.294041254 | -3.020982203 |
| COUMESTROL | GFRA1 | increases expression | Comparative Toxicogenomics Database | 0.294041254 | -3.020982203 |
| Tetradioxin | GFRA1 | increases expression | Comparative Toxicogenomics Database | 0.294041254 | -3.020982203 |
| atrazine | GLIS1 | increases expression | Comparative Toxicogenomics Database | NA | NA |
| 3,3',4,4',5-Pentachlorobiphenyl | GPR68 | increases expression | Comparative Toxicogenomics Database | NA | NA |
| benzo[a]pyrene | GPR68 | increases expression | Comparative Toxicogenomics Database | NA | NA |
| Tetradioxin | GPR68 | increases expression | Comparative Toxicogenomics Database | NA | NA |
| calcitriol | GPR153 | increases expression | Comparative Toxicogenomics Database | NA | NA |
| testosterone | GPR153 | increases expression | Comparative Toxicogenomics Database | NA | NA |
| ethanol | GPX3 | increases expression | Comparative Toxicogenomics Database | 0.289344246 | -2.465959798 |
| TBTO | GPX3 | increases expression | Comparative Toxicogenomics Database | 0.289344246 | -2.465959798 |
| progesterone | GPX3 | increases expression | Comparative Toxicogenomics Database | 0.289344246 | -2.465959798 |
| CHEBI:18224 | GPX3 | increases expression | Comparative Toxicogenomics Database | 0.289344246 | -2.465959798 |
| alpha-Hexylcinnamaldehyde | GPX3 | increases expression | Comparative Toxicogenomics Database | 0.289344246 | -2.465959798 |
| azathioprine | GPX3 | increases expression | Comparative Toxicogenomics Database | 0.289344246 | -2.465959798 |
| Glycidamide | GPX3 | increases expression | Comparative Toxicogenomics Database | 0.289344246 | -2.465959798 |
| 6-Mercaptopurine | GPX3 | increases expression | Comparative Toxicogenomics Database | 0.289344246 | -2.465959798 |
| acetaminophen | GPX3 | increases expression | Comparative Toxicogenomics Database | 0.289344246 | -2.465959798 |
| CHLOROBENZENE | GPX3 | increases expression | Comparative Toxicogenomics Database | 0.289344246 | -2.465959798 |
| ciglitazone | GPX3 | increases expression | Comparative Toxicogenomics Database | 0.289344246 | -2.465959798 |
| disulfiram | GPX3 | increases expression | Comparative Toxicogenomics Database | 0.289344246 | -2.465959798 |
| eugenol | GPX3 | increases expression | Comparative Toxicogenomics Database | 0.289344246 | -2.465959798 |
| Decitabine | GPX3 | increases expression | Comparative Toxicogenomics Database | 0.289344246 | -2.465959798 |
| dexamethasone | GPX3 | increases expression | Comparative Toxicogenomics Database | 0.289344246 | -2.465959798 |
| SELENIUM | GPX3 | increases expression | Comparative Toxicogenomics Database | 0.289344246 | -2.465959798 |
| 3,3',4,4',5-Pentachlorobiphenyl | GPX3 | increases expression | Comparative Toxicogenomics Database | 0.289344246 | -2.465959798 |
| estradiol | GPX3 | increases expression | Comparative Toxicogenomics Database | 0.289344246 | -2.465959798 |
| 7646-79-9 | GPX3 | increases expression | Comparative Toxicogenomics Database | 0.289344246 | -2.465959798 |
| Tetradioxin | GPX3 | increases expression | Comparative Toxicogenomics Database | 0.289344246 | -2.465959798 |
| 1-chloro-2,4-dinitrobenzene | GPX3 | increases expression | Comparative Toxicogenomics Database | 0.289344246 | -2.465959798 |
| quercetin | GSN | increases expression | Comparative Toxicogenomics Database | 0.243451973 | -1.371737208 |
| metformin | GSN | increases expression | Comparative Toxicogenomics Database | 0.243451973 | -1.371737208 |
| atrazine | GSN | increases expression | Comparative Toxicogenomics Database | 0.243451973 | -1.371737208 |
| calcitriol | GSN | increases expression | Comparative Toxicogenomics Database | 0.243451973 | -1.371737208 |
| SELENIUM | GSN | increases expression | Comparative Toxicogenomics Database | 0.243451973 | -1.371737208 |
| benzo[a]pyrene | GSN | increases expression | Comparative Toxicogenomics Database | 0.243451973 | -1.371737208 |
| troglitazone | GSN | increases expression | Comparative Toxicogenomics Database | 0.243451973 | -1.371737208 |
| hydralazine | GSN | increases expression | Comparative Toxicogenomics Database | 0.243451973 | -1.371737208 |
| Tetradioxin | GSN | increases expression | Comparative Toxicogenomics Database | 0.243451973 | -1.371737208 |
| rosiglitazone | GSN | increases expression | Comparative Toxicogenomics Database | 0.243451973 | -1.371737208 |
| Tamibarotene | GSN | increases expression | Comparative Toxicogenomics Database | 0.243451973 | -1.371737208 |
| testosterone | GSN | increases expression | Comparative Toxicogenomics Database | 0.243451973 | -1.371737208 |
| Bortezomib | GZMB | increases expression | Comparative Toxicogenomics Database | NA | NA |
| tamoxifen | GZMB | increases expression | Comparative Toxicogenomics Database | NA | NA |
| Fulvestrant | GZMB | increases expression | Comparative Toxicogenomics Database | NA | NA |
| 67526-95-8 | HAVCR2 | increases expression | Comparative Toxicogenomics Database | NA | NA |
| quercetin | HKR1 | increases expression | Comparative Toxicogenomics Database | 0.241722639 | -1.122074863 |
| Bortezomib | HKR1 | increases expression | Comparative Toxicogenomics Database | 0.241722639 | -1.122074863 |
| GDC-0941 | HKR1 | increases expression | Comparative Toxicogenomics Database | 0.241722639 | -1.122074863 |
| progesterone | ID4 | increases expression | Comparative Toxicogenomics Database | 0.223027193 | -2.231245277 |
| ZINC | ID4 | increases expression | Comparative Toxicogenomics Database | 0.223027193 | -2.231245277 |
| 17-Hydroxyandrostan-3-one | ID4 | increases expression | Comparative Toxicogenomics Database | 0.223027193 | -2.231245277 |
| formaldehyde | ID4 | increases expression | Comparative Toxicogenomics Database | 0.223027193 | -2.231245277 |
| Decitabine | ID4 | increases expression | Comparative Toxicogenomics Database | 0.223027193 | -2.231245277 |
| tamoxifen | ID4 | increases expression | Comparative Toxicogenomics Database | 0.223027193 | -2.231245277 |
| genistein | ID4 | increases expression | Comparative Toxicogenomics Database | 0.223027193 | -2.231245277 |
| raloxifene | ID4 | increases expression | Comparative Toxicogenomics Database | 0.223027193 | -2.231245277 |
| Torcetrapib | ID4 | increases expression | Comparative Toxicogenomics Database | 0.223027193 | -2.231245277 |
| Caspan | IFITM1 | increases expression | Comparative Toxicogenomics Database | NA | NA |
| 5-Fluorouracil | IFITM1 | increases expression | Comparative Toxicogenomics Database | NA | NA |
| atrazine | IFITM1 | increases expression | Comparative Toxicogenomics Database | NA | NA |
| Vitinoin | IFITM1 | increases expression | Comparative Toxicogenomics Database | NA | NA |
| tamoxifen | IFITM1 | increases expression | Comparative Toxicogenomics Database | NA | NA |
| SELENIUM | IFITM1 | increases expression | Comparative Toxicogenomics Database | NA | NA |
| estradiol | IFITM1 | increases expression | Comparative Toxicogenomics Database | NA | NA |
| chlorophyllin | IFITM1 | increases expression | Comparative Toxicogenomics Database | NA | NA |
| benzene | IFITM1 | increases expression | Comparative Toxicogenomics Database | NA | NA |
| benzo[a]pyrene | IFITM1 | increases expression | Comparative Toxicogenomics Database | NA | NA |
| 3'-Azido-3'-deoxythymidine | IFITM1 | increases expression | Comparative Toxicogenomics Database | NA | NA |
| 1-Methyl-3-nitro-1-nitrosoguanidine | IFITM1 | increases expression | Comparative Toxicogenomics Database | NA | NA |
| Tetradioxin | IFITM1 | increases expression | Comparative Toxicogenomics Database | NA | NA |
| estradiol | IGF2BP2 | increases expression | Comparative Toxicogenomics Database | NA | NA |
| hydralazine | IGF2BP2 | increases expression | Comparative Toxicogenomics Database | NA | NA |
| formaldehyde | IL21R | increases expression | Comparative Toxicogenomics Database | NA | NA |
| benzene | IL21R | increases expression | Comparative Toxicogenomics Database | NA | NA |
| benzo[a]pyrene | ITIH5 | increases expression | Comparative Toxicogenomics Database | 0.276199363 | -1.552912539 |
| 7646-79-9 | ITIH5 | increases expression | Comparative Toxicogenomics Database | 0.276199363 | -1.552912539 |
| progesterone | KLHL23 | increases expression | Comparative Toxicogenomics Database | 0.233203929 | -1.124453399 |
| hydralazine | KLHL23 | increases expression | Comparative Toxicogenomics Database | 0.233203929 | -1.124453399 |
| atrazine | LAIR1 | increases expression | Comparative Toxicogenomics Database | NA | NA |
| carmustine | LAIR1 | increases expression | Comparative Toxicogenomics Database | NA | NA |
| AM580 | LAIR1 | increases expression | Comparative Toxicogenomics Database | NA | NA |
| rosiglitazone | LAIR1 | increases expression | Comparative Toxicogenomics Database | NA | NA |
| N-NITROSODIETHYLAMINE | LAMA3 | increases expression | Comparative Toxicogenomics Database | NA | NA |
| dexamethasone | LAMA3 | increases expression | Comparative Toxicogenomics Database | NA | NA |
| estradiol | LAMA3 | increases expression | Comparative Toxicogenomics Database | NA | NA |
| IBMX | LAMA3 | increases expression | Comparative Toxicogenomics Database | NA | NA |
| benzo[a]pyrene | LAMA3 | increases expression | Comparative Toxicogenomics Database | NA | NA |
| Tetradioxin | LAMA3 | increases expression | Comparative Toxicogenomics Database | NA | NA |
| Vitinoin | LCK | increases expression | Comparative Toxicogenomics Database | NA | NA |
| cycloheximide | LCK | increases expression | Comparative Toxicogenomics Database | NA | NA |
| Chromium(VI) | LCK | increases expression | Comparative Toxicogenomics Database | NA | NA |
| Tetradioxin | LCK | increases expression | Comparative Toxicogenomics Database | NA | NA |
| Tamibarotene | LCK | increases expression | Comparative Toxicogenomics Database | NA | NA |
| ZINC | LILRB1 | increases expression | Comparative Toxicogenomics Database | NA | NA |
| Andriol | LILRB1 | increases expression | Comparative Toxicogenomics Database | NA | NA |
| norgestrel | LILRB1 | increases expression | Comparative Toxicogenomics Database | NA | NA |
| 7646-79-9 | LILRB1 | increases expression | Comparative Toxicogenomics Database | NA | NA |
| atrazine | LILRB4 | increases expression | Comparative Toxicogenomics Database | NA | NA |
| 3'-Azido-3'-deoxythymidine | LILRB4 | increases expression | Comparative Toxicogenomics Database | NA | NA |
| dexamethasone | LTC4S | increases expression | Comparative Toxicogenomics Database | 0.293224488 | -1.234759152 |
| 7646-79-9 | MAGI2 | increases expression | Comparative Toxicogenomics Database | 0.293822229 | -1.843294093 |
| 3-amino-1,2,4-triazole | MAP1LC3A | increases expression | Comparative Toxicogenomics Database | 0.216028252 | -1.809105434 |
| rapamycin | MAP1LC3A | increases expression | Comparative Toxicogenomics Database | 0.216028252 | -1.809105434 |
| Dihydrocapsaicin | MAP1LC3A | increases expression | Comparative Toxicogenomics Database | 0.216028252 | -1.809105434 |
| VANADIUM | MFAP2 | increases expression | Comparative Toxicogenomics Database | NA | NA |
| acetaminophen | MFAP2 | increases expression | Comparative Toxicogenomics Database | NA | NA |
| Vitinoin | MFAP2 | increases expression | Comparative Toxicogenomics Database | NA | NA |
| SELENIUM | MFAP2 | increases expression | Comparative Toxicogenomics Database | NA | NA |
| COPPER | MFAP2 | increases expression | Comparative Toxicogenomics Database | NA | NA |
| quercetin | MKI67 | increases expression | Comparative Toxicogenomics Database | NA | NA |
| progesterone | MKI67 | increases expression | Comparative Toxicogenomics Database | NA | NA |
| Caspan | MKI67 | increases expression | Comparative Toxicogenomics Database | NA | NA |
| Enterolactone | MKI67 | increases expression | Comparative Toxicogenomics Database | NA | NA |
| DIMEFOX | MKI67 | increases expression | Comparative Toxicogenomics Database | NA | NA |
| acetaminophen | MKI67 | increases expression | Comparative Toxicogenomics Database | NA | NA |
| MeIQx | MKI67 | increases expression | Comparative Toxicogenomics Database | NA | NA |
| atrazine | MKI67 | increases expression | Comparative Toxicogenomics Database | NA | NA |
| Benzo[b]fluoranthene | MKI67 | increases expression | Comparative Toxicogenomics Database | NA | NA |
| ketamine | MKI67 | increases expression | Comparative Toxicogenomics Database | NA | NA |
| ciglitazone | MKI67 | increases expression | Comparative Toxicogenomics Database | NA | NA |
| N-NITROSODIETHYLAMINE | MKI67 | increases expression | Comparative Toxicogenomics Database | NA | NA |
| tamoxifen | MKI67 | increases expression | Comparative Toxicogenomics Database | NA | NA |
| estradiol | MKI67 | increases expression | Comparative Toxicogenomics Database | NA | NA |
| Phytoestrogens | MKI67 | increases expression | Comparative Toxicogenomics Database | NA | NA |
| resveratrol | MKI67 | increases expression | Comparative Toxicogenomics Database | NA | NA |
| arbutin | MKI67 | increases expression | Comparative Toxicogenomics Database | NA | NA |
| troglitazone | MKI67 | increases expression | Comparative Toxicogenomics Database | NA | NA |
| COUMESTROL | MKI67 | increases expression | Comparative Toxicogenomics Database | NA | NA |
| quercetin | MLPH | increases expression | Comparative Toxicogenomics Database | 0.284758042 | -4.518229538 |
| progesterone | MLPH | increases expression | Comparative Toxicogenomics Database | 0.284758042 | -4.518229538 |
| genistein | MLPH | increases expression | Comparative Toxicogenomics Database | 0.284758042 | -4.518229538 |
| estradiol | MLPH | increases expression | Comparative Toxicogenomics Database | 0.284758042 | -4.518229538 |
| 1-NITROPYRENE | MLPH | increases expression | Comparative Toxicogenomics Database | 0.284758042 | -4.518229538 |
| benzo[a]pyrene | MLPH | increases expression | Comparative Toxicogenomics Database | 0.284758042 | -4.518229538 |
| O,P'-DDT | MLPH | increases expression | Comparative Toxicogenomics Database | 0.284758042 | -4.518229538 |
| Tetradioxin | MLPH | increases expression | Comparative Toxicogenomics Database | 0.284758042 | -4.518229538 |
| ZEARALENONE | MLPH | increases expression | Comparative Toxicogenomics Database | 0.284758042 | -4.518229538 |
| 3-(1-methylpyrrolidin-2-yl)pyridine | MLPH | increases expression | Comparative Toxicogenomics Database | 0.284758042 | -4.518229538 |
| diethylstilbestrol | MLPH | increases expression | Comparative Toxicogenomics Database | 0.284758042 | -4.518229538 |
| quercetin | MMP13 | increases expression | Comparative Toxicogenomics Database | NA | NA |
| chitosamine | MMP13 | increases expression | Comparative Toxicogenomics Database | NA | NA |
| simvastatin | MMP13 | increases expression | Comparative Toxicogenomics Database | NA | NA |
| CADMIUM | MMP13 | increases expression | Comparative Toxicogenomics Database | NA | NA |
| bicalutamide | MMP13 | increases expression | Comparative Toxicogenomics Database | NA | NA |
| doxycycline | MMP13 | increases expression | Comparative Toxicogenomics Database | NA | NA |
| trolox | MMP13 | increases expression | Comparative Toxicogenomics Database | NA | NA |
| norfloxacin | MMP13 | increases expression | Comparative Toxicogenomics Database | NA | NA |
| Vitinoin | MMP13 | increases expression | Comparative Toxicogenomics Database | NA | NA |
| curcumin | MMP13 | increases expression | Comparative Toxicogenomics Database | NA | NA |
| cycloheximide | MMP13 | increases expression | Comparative Toxicogenomics Database | NA | NA |
| tetracycline | MMP13 | increases expression | Comparative Toxicogenomics Database | NA | NA |
| Decitabine | MMP13 | increases expression | Comparative Toxicogenomics Database | NA | NA |
| 1,9-Pyrazoloanthrone | MMP13 | increases expression | Comparative Toxicogenomics Database | NA | NA |
| SELENIUM | MMP13 | increases expression | Comparative Toxicogenomics Database | NA | NA |
| estradiol | MMP13 | increases expression | Comparative Toxicogenomics Database | NA | NA |
| Dibenziodolium | MMP13 | increases expression | Comparative Toxicogenomics Database | NA | NA |
| ciprofloxacin | MMP13 | increases expression | Comparative Toxicogenomics Database | NA | NA |
| resveratrol | MMP13 | increases expression | Comparative Toxicogenomics Database | NA | NA |
| chloramphenicol | MMP13 | increases expression | Comparative Toxicogenomics Database | NA | NA |
| benzene | MMP13 | increases expression | Comparative Toxicogenomics Database | NA | NA |
| Acetovanillone | MMP13 | increases expression | Comparative Toxicogenomics Database | NA | NA |
| clindamycin | MMP13 | increases expression | Comparative Toxicogenomics Database | NA | NA |
| Masoprocol | MMP13 | increases expression | Comparative Toxicogenomics Database | NA | NA |
| 635-65-4 | MMP13 | increases expression | Comparative Toxicogenomics Database | NA | NA |
| minocycline | MMP13 | increases expression | Comparative Toxicogenomics Database | NA | NA |
| Tetradioxin | MMP13 | increases expression | Comparative Toxicogenomics Database | NA | NA |
| 3-(1-methylpyrrolidin-2-yl)pyridine | MMP13 | increases expression | Comparative Toxicogenomics Database | NA | NA |
| ofloxacin | MMP13 | increases expression | Comparative Toxicogenomics Database | NA | NA |
| TPEN | MX2 | increases expression | Comparative Toxicogenomics Database | NA | NA |
| estradiol | MX2 | increases expression | Comparative Toxicogenomics Database | NA | NA |
| benzo[a]pyrene | MX2 | increases expression | Comparative Toxicogenomics Database | NA | NA |
| 3'-Azido-3'-deoxythymidine | MX2 | increases expression | Comparative Toxicogenomics Database | NA | NA |
| atrazine | MYEF2 | increases expression | Comparative Toxicogenomics Database | 0.274428469 | -1.628751292 |
| amiodarone | MYEF2 | increases expression | Comparative Toxicogenomics Database | 0.274428469 | -1.628751292 |
| formaldehyde | MYEF2 | increases expression | Comparative Toxicogenomics Database | 0.274428469 | -1.628751292 |
| CADMIUM | MYH14 | increases expression | Comparative Toxicogenomics Database | 0.296898242 | -2.574099263 |
| genistein | MYH14 | increases expression | Comparative Toxicogenomics Database | 0.296898242 | -2.574099263 |
| ciglitazone | NDRG2 | increases expression | Comparative Toxicogenomics Database | 0.265615307 | -2.47552837 |
| oxygen | NDRG2 | increases expression | Comparative Toxicogenomics Database | 0.265615307 | -2.47552837 |
| Silica | NFIX | increases expression | Comparative Toxicogenomics Database | 0.315751632 | -2.103223374 |
| atrazine | NFIX | increases expression | Comparative Toxicogenomics Database | 0.315751632 | -2.103223374 |
| resveratrol | NFIX | increases expression | Comparative Toxicogenomics Database | 0.315751632 | -2.103223374 |
| ionomycin | NMI | increases expression | Comparative Toxicogenomics Database | NA | NA |
| 5-azacytidine | NMI | increases expression | Comparative Toxicogenomics Database | NA | NA |
| estradiol | NMI | increases expression | Comparative Toxicogenomics Database | NA | NA |
| arbutin | NMI | increases expression | Comparative Toxicogenomics Database | NA | NA |
| benzo[a]pyrene | NMI | increases expression | Comparative Toxicogenomics Database | NA | NA |
| Tetradioxin | NMI | increases expression | Comparative Toxicogenomics Database | NA | NA |
| Cylindrospermopsin | NOSTRIN | increases expression | Comparative Toxicogenomics Database | 0.201190527 | -2.279426195 |
| progesterone | NR3C2 | increases expression | Comparative Toxicogenomics Database | 0.226967362 | -3.338840461 |
| carbamazepine | NR3C2 | increases expression | Comparative Toxicogenomics Database | 0.226967362 | -3.338840461 |
| ALDOSTERONE | NR3C2 | increases expression | Comparative Toxicogenomics Database | 0.226967362 | -3.338840461 |
| dexamethasone | NR3C2 | increases expression | Comparative Toxicogenomics Database | 0.226967362 | -3.338840461 |
| estradiol | NR3C2 | increases expression | Comparative Toxicogenomics Database | 0.226967362 | -3.338840461 |
| progesterone | NTRK3 | increases expression | Comparative Toxicogenomics Database | 0.31859149 | -3.0973793 |
| atrazine | NTRK3 | increases expression | Comparative Toxicogenomics Database | 0.31859149 | -3.0973793 |
| amiodarone | NTRK3 | increases expression | Comparative Toxicogenomics Database | 0.31859149 | -3.0973793 |
| SARIN | NTRK3 | increases expression | Comparative Toxicogenomics Database | 0.31859149 | -3.0973793 |
| estradiol | NTRK3 | increases expression | Comparative Toxicogenomics Database | 0.31859149 | -3.0973793 |
| MERCURY | NTRK3 | increases expression | Comparative Toxicogenomics Database | 0.31859149 | -3.0973793 |
| quercetin | NUPR1 | increases expression | Comparative Toxicogenomics Database | 0.248176617 | -1.215063319 |
| 5,6-BENZOFLAVONE | NUPR1 | increases expression | Comparative Toxicogenomics Database | 0.248176617 | -1.215063319 |
| chlorcyclizine | NUPR1 | increases expression | Comparative Toxicogenomics Database | 0.248176617 | -1.215063319 |
| clomipramine | NUPR1 | increases expression | Comparative Toxicogenomics Database | 0.248176617 | -1.215063319 |
| sertraline | NUPR1 | increases expression | Comparative Toxicogenomics Database | 0.248176617 | -1.215063319 |
| 14-Deoxy-11,12-didehydroandrographolide | NUPR1 | increases expression | Comparative Toxicogenomics Database | 0.248176617 | -1.215063319 |
| Bortezomib | NUPR1 | increases expression | Comparative Toxicogenomics Database | 0.248176617 | -1.215063319 |
| phenobarbital | NUPR1 | increases expression | Comparative Toxicogenomics Database | 0.248176617 | -1.215063319 |
| PERHEXILINE | NUPR1 | increases expression | Comparative Toxicogenomics Database | 0.248176617 | -1.215063319 |
| amitriptyline | NUPR1 | increases expression | Comparative Toxicogenomics Database | 0.248176617 | -1.215063319 |
| thermozymocidin | NUPR1 | increases expression | Comparative Toxicogenomics Database | 0.248176617 | -1.215063319 |
| citalopram | NUPR1 | increases expression | Comparative Toxicogenomics Database | 0.248176617 | -1.215063319 |
| doxepin | NUPR1 | increases expression | Comparative Toxicogenomics Database | 0.248176617 | -1.215063319 |
| imipramine | NUPR1 | increases expression | Comparative Toxicogenomics Database | 0.248176617 | -1.215063319 |
| Dexanabinol | NUPR1 | increases expression | Comparative Toxicogenomics Database | 0.248176617 | -1.215063319 |
| Quinine | NUPR1 | increases expression | Comparative Toxicogenomics Database | 0.248176617 | -1.215063319 |
| Zimelidine | NUPR1 | increases expression | Comparative Toxicogenomics Database | 0.248176617 | -1.215063319 |
| amiodarone | NUPR1 | increases expression | Comparative Toxicogenomics Database | 0.248176617 | -1.215063319 |
| Dronabinol | NUPR1 | increases expression | Comparative Toxicogenomics Database | 0.248176617 | -1.215063319 |
| fluoxetine | NUPR1 | increases expression | Comparative Toxicogenomics Database | 0.248176617 | -1.215063319 |
| formaldehyde | NUPR1 | increases expression | Comparative Toxicogenomics Database | 0.248176617 | -1.215063319 |
| COBALT | NUPR1 | increases expression | Comparative Toxicogenomics Database | 0.248176617 | -1.215063319 |
| tetracycline | NUPR1 | increases expression | Comparative Toxicogenomics Database | 0.248176617 | -1.215063319 |
| ketoconazole | NUPR1 | increases expression | Comparative Toxicogenomics Database | 0.248176617 | -1.215063319 |
| 67526-95-8 | NUPR1 | increases expression | Comparative Toxicogenomics Database | 0.248176617 | -1.215063319 |
| tamoxifen | NUPR1 | increases expression | Comparative Toxicogenomics Database | 0.248176617 | -1.215063319 |
| haloperidol | NUPR1 | increases expression | Comparative Toxicogenomics Database | 0.248176617 | -1.215063319 |
| pentamidine | NUPR1 | increases expression | Comparative Toxicogenomics Database | 0.248176617 | -1.215063319 |
| flecainide | NUPR1 | increases expression | Comparative Toxicogenomics Database | 0.248176617 | -1.215063319 |
| COPPER | NUPR1 | increases expression | Comparative Toxicogenomics Database | 0.248176617 | -1.215063319 |
| sotalol | NUPR1 | increases expression | Comparative Toxicogenomics Database | 0.248176617 | -1.215063319 |
| loratadine | NUPR1 | increases expression | Comparative Toxicogenomics Database | 0.248176617 | -1.215063319 |
| isoniazid | NUPR1 | increases expression | Comparative Toxicogenomics Database | 0.248176617 | -1.215063319 |
| benzo[a]pyrene | NUPR1 | increases expression | Comparative Toxicogenomics Database | 0.248176617 | -1.215063319 |
| thioridazine | NUPR1 | increases expression | Comparative Toxicogenomics Database | 0.248176617 | -1.215063319 |
| clozapine | NUPR1 | increases expression | Comparative Toxicogenomics Database | 0.248176617 | -1.215063319 |
| erythromycin | NUPR1 | increases expression | Comparative Toxicogenomics Database | 0.248176617 | -1.215063319 |
| hydralazine | NUPR1 | increases expression | Comparative Toxicogenomics Database | 0.248176617 | -1.215063319 |
| Tetradioxin | NUPR1 | increases expression | Comparative Toxicogenomics Database | 0.248176617 | -1.215063319 |
| 8-HYDROXYQUINOLINE | NUPR1 | increases expression | Comparative Toxicogenomics Database | 0.248176617 | -1.215063319 |
| chlorpromazine | NUPR1 | increases expression | Comparative Toxicogenomics Database | 0.248176617 | -1.215063319 |
| ofloxacin | NUPR1 | increases expression | Comparative Toxicogenomics Database | 0.248176617 | -1.215063319 |
| amantadine | OAS2 | increases expression | Comparative Toxicogenomics Database | NA | NA |
| 5-Fluorouracil | OAS2 | increases expression | Comparative Toxicogenomics Database | NA | NA |
| Bifendate | OAS2 | increases expression | Comparative Toxicogenomics Database | NA | NA |
| Decitabine | OAS2 | increases expression | Comparative Toxicogenomics Database | NA | NA |
| estradiol | OAS2 | increases expression | Comparative Toxicogenomics Database | NA | NA |
| chlorophyllin | OAS2 | increases expression | Comparative Toxicogenomics Database | NA | NA |
| benzo[a]pyrene | OAS2 | increases expression | Comparative Toxicogenomics Database | NA | NA |
| AM580 | OAS2 | increases expression | Comparative Toxicogenomics Database | NA | NA |
| 3'-Azido-3'-deoxythymidine | OAS2 | increases expression | Comparative Toxicogenomics Database | NA | NA |
| Tetradioxin | OAS2 | increases expression | Comparative Toxicogenomics Database | NA | NA |
| Tamibarotene | OAS2 | increases expression | Comparative Toxicogenomics Database | NA | NA |
| 4-Oxoretinol | OLR1 | increases expression | Comparative Toxicogenomics Database | NA | NA |
| quercetin | OLR1 | increases expression | Comparative Toxicogenomics Database | NA | NA |
| POVPC | OLR1 | increases expression | Comparative Toxicogenomics Database | NA | NA |
| Isorhamnetin | OLR1 | increases expression | Comparative Toxicogenomics Database | NA | NA |
| ALDOSTERONE | OLR1 | increases expression | Comparative Toxicogenomics Database | NA | NA |
| Apocarotenal | OLR1 | increases expression | Comparative Toxicogenomics Database | NA | NA |
| retinol | OLR1 | increases expression | Comparative Toxicogenomics Database | NA | NA |
| CHROMIUM | OLR1 | increases expression | Comparative Toxicogenomics Database | NA | NA |
| SARIN | OLR1 | increases expression | Comparative Toxicogenomics Database | NA | NA |
| spironolactone | OLR1 | increases expression | Comparative Toxicogenomics Database | NA | NA |
| Telmisartan | OLR1 | increases expression | Comparative Toxicogenomics Database | NA | NA |
| Alitretinoin | OLR1 | increases expression | Comparative Toxicogenomics Database | NA | NA |
| Acetovanillone | OLR1 | increases expression | Comparative Toxicogenomics Database | NA | NA |
| AM580 | OLR1 | increases expression | Comparative Toxicogenomics Database | NA | NA |
| beta-carotene | OLR1 | increases expression | Comparative Toxicogenomics Database | NA | NA |
| amlodipine | OLR1 | increases expression | Comparative Toxicogenomics Database | NA | NA |
| rosiglitazone | OLR1 | increases expression | Comparative Toxicogenomics Database | NA | NA |
| ZINC | OSBPL1A | increases expression | Comparative Toxicogenomics Database | 0.312906707 | -1.052775815 |
| calcitriol | OSBPL1A | increases expression | Comparative Toxicogenomics Database | 0.312906707 | -1.052775815 |
| testosterone | OSBPL1A | increases expression | Comparative Toxicogenomics Database | 0.312906707 | -1.052775815 |
| aspirin | OSR1 | increases expression | Comparative Toxicogenomics Database | 0.225490876 | -3.545097818 |
| COPPER | P2RY6 | increases expression | Comparative Toxicogenomics Database | NA | NA |
| benzo[a]pyrene | P2RY6 | increases expression | Comparative Toxicogenomics Database | NA | NA |
| acetaminophen | PALM | increases expression | Comparative Toxicogenomics Database | 0.220100397 | -2.151563088 |
| genistein | PALM | increases expression | Comparative Toxicogenomics Database | 0.220100397 | -2.151563088 |
| estradiol | PALM | increases expression | Comparative Toxicogenomics Database | 0.220100397 | -2.151563088 |
| Bortezomib | PCYT1A | increases expression | Comparative Toxicogenomics Database | NA | NA |
| benzo[a]pyrene | PCYT1A | increases expression | Comparative Toxicogenomics Database | NA | NA |
| Torcetrapib | PCYT1A | increases expression | Comparative Toxicogenomics Database | NA | NA |
| 7646-79-9 | PCYT1A | increases expression | Comparative Toxicogenomics Database | NA | NA |
| Tetradioxin | PCYT1A | increases expression | Comparative Toxicogenomics Database | NA | NA |
| quercetin | PDK4 | increases expression | Comparative Toxicogenomics Database | 0.221555122 | -3.123631641 |
| bezafibrate | PDK4 | increases expression | Comparative Toxicogenomics Database | 0.221555122 | -3.123631641 |
| estradiol | PDK4 | increases expression | Comparative Toxicogenomics Database | 0.221555122 | -3.123631641 |
| 3,4-DICHLOROANILINE | PDK4 | increases expression | Comparative Toxicogenomics Database | 0.221555122 | -3.123631641 |
| troglitazone | PDK4 | increases expression | Comparative Toxicogenomics Database | 0.221555122 | -3.123631641 |
| Torcetrapib | PDK4 | increases expression | Comparative Toxicogenomics Database | 0.221555122 | -3.123631641 |
| GDC-0941 | PDK4 | increases expression | Comparative Toxicogenomics Database | 0.221555122 | -3.123631641 |
| Tetradioxin | PDK4 | increases expression | Comparative Toxicogenomics Database | 0.221555122 | -3.123631641 |
| rosiglitazone | PDK4 | increases expression | Comparative Toxicogenomics Database | 0.221555122 | -3.123631641 |
| phenol | PER2 | increases expression | Comparative Toxicogenomics Database | 0.281371364 | -1.123017003 |
| progesterone | PER2 | increases expression | Comparative Toxicogenomics Database | 0.281371364 | -1.123017003 |
| phenobarbital | PER2 | increases expression | Comparative Toxicogenomics Database | 0.281371364 | -1.123017003 |
| curcumin | PER2 | increases expression | Comparative Toxicogenomics Database | 0.281371364 | -1.123017003 |
| Torcetrapib | PER2 | increases expression | Comparative Toxicogenomics Database | 0.281371364 | -1.123017003 |
| 7,8-Benzoflavone | PGM1 | increases expression | Comparative Toxicogenomics Database | 0.336771861 | -1.558423882 |
| oxygen | PGM1 | increases expression | Comparative Toxicogenomics Database | 0.336771861 | -1.558423882 |
| Capsaicin | PGM1 | increases expression | Comparative Toxicogenomics Database | 0.336771861 | -1.558423882 |
| 7646-79-9 | PGM1 | increases expression | Comparative Toxicogenomics Database | 0.336771861 | -1.558423882 |
| hydralazine | PGM1 | increases expression | Comparative Toxicogenomics Database | 0.336771861 | -1.558423882 |
| acetaminophen | PHC1 | increases expression | Comparative Toxicogenomics Database | 0.268768799 | -1.275993191 |
| atrazine | PHC1 | increases expression | Comparative Toxicogenomics Database | 0.268768799 | -1.275993191 |
| N-NITROSODIETHYLAMINE | PI3 | increases expression | Comparative Toxicogenomics Database | NA | NA |
| COPPER | PI3 | increases expression | Comparative Toxicogenomics Database | NA | NA |
| 3,4-DICHLOROANILINE | PI3 | increases expression | Comparative Toxicogenomics Database | NA | NA |
| troglitazone | PI3 | increases expression | Comparative Toxicogenomics Database | NA | NA |
| diuron | PI3 | increases expression | Comparative Toxicogenomics Database | NA | NA |
| phenol | PIK3CD | increases expression | Comparative Toxicogenomics Database | NA | NA |
| Tylophorine | PIK3CD | increases expression | Comparative Toxicogenomics Database | NA | NA |
| ARSENIC | PIK3CD | increases expression | Comparative Toxicogenomics Database | NA | NA |
| atrazine | PIK3CD | increases expression | Comparative Toxicogenomics Database | NA | NA |
| Decitabine | PIK3CD | increases expression | Comparative Toxicogenomics Database | NA | NA |
| N-NITROSODIMETHYLAMINE | PIK3CD | increases expression | Comparative Toxicogenomics Database | NA | NA |
| SELENIUM | PIK3CD | increases expression | Comparative Toxicogenomics Database | NA | NA |
| Tamibarotene | PIK3CD | increases expression | Comparative Toxicogenomics Database | NA | NA |
| aspirin | PITX1 | increases expression | Comparative Toxicogenomics Database | 0.233066872 | -1.381720039 |
| SELENIUM | PITX1 | increases expression | Comparative Toxicogenomics Database | 0.233066872 | -1.381720039 |
| estradiol | PITX1 | increases expression | Comparative Toxicogenomics Database | 0.233066872 | -1.381720039 |
| doxorubicin | PITX1 | increases expression | Comparative Toxicogenomics Database | 0.233066872 | -1.381720039 |
| quercetin | PLAGL1 | increases expression | Comparative Toxicogenomics Database | 0.200861299 | -2.004985762 |
| Caspan | PLAGL1 | increases expression | Comparative Toxicogenomics Database | 0.200861299 | -2.004985762 |
| Decitabine | PLAGL1 | increases expression | Comparative Toxicogenomics Database | 0.200861299 | -2.004985762 |
| 7646-79-9 | PLAGL1 | increases expression | Comparative Toxicogenomics Database | 0.200861299 | -2.004985762 |
| calcitriol | PLEKHG6 | increases expression | Comparative Toxicogenomics Database | 0.222825086 | -2.390192764 |
| testosterone | PLEKHG6 | increases expression | Comparative Toxicogenomics Database | 0.222825086 | -2.390192764 |
| ZINC | PMEPA1 | increases expression | Comparative Toxicogenomics Database | NA | NA |
| (17S)-17-hydroxy-13,17-dimethyl-1,2,6,7,8,14,15,16-octahydrocyclopenta[a]phenanthren-3-one | PMEPA1 | increases expression | Comparative Toxicogenomics Database | NA | NA |
| 17-Hydroxyandrostan-3-one | PMEPA1 | increases expression | Comparative Toxicogenomics Database | NA | NA |
| Decitabine | PMEPA1 | increases expression | Comparative Toxicogenomics Database | NA | NA |
| indomethacin | PMEPA1 | increases expression | Comparative Toxicogenomics Database | NA | NA |
| benzene | PMEPA1 | increases expression | Comparative Toxicogenomics Database | NA | NA |
| Allococaine | PMEPA1 | increases expression | Comparative Toxicogenomics Database | NA | NA |
| Dasatinib | PODN | increases expression | Comparative Toxicogenomics Database | 0.285607666 | -2.328564919 |
| amiodarone | PODN | increases expression | Comparative Toxicogenomics Database | 0.285607666 | -2.328564919 |
| bezafibrate | PPARG | increases expression | Comparative Toxicogenomics Database | 0.262871983 | -2.458926112 |
| ethanol | PPARG | increases expression | Comparative Toxicogenomics Database | 0.262871983 | -2.458926112 |
| N-Acetyl-L-cysteine | PPARG | increases expression | Comparative Toxicogenomics Database | 0.262871983 | -2.458926112 |
| phenol | PPARG | increases expression | Comparative Toxicogenomics Database | 0.262871983 | -2.458926112 |
| p-Phenylenediamine | PPARG | increases expression | Comparative Toxicogenomics Database | 0.262871983 | -2.458926112 |
| PHENCYCLIDINE | PPARG | increases expression | Comparative Toxicogenomics Database | 0.262871983 | -2.458926112 |
| acetaldehyde | PPARG | increases expression | Comparative Toxicogenomics Database | 0.262871983 | -2.458926112 |
| chitosamine | PPARG | increases expression | Comparative Toxicogenomics Database | 0.262871983 | -2.458926112 |
| Monascin | PPARG | increases expression | Comparative Toxicogenomics Database | 0.262871983 | -2.458926112 |
| ZINC | PPARG | increases expression | Comparative Toxicogenomics Database | 0.262871983 | -2.458926112 |
| DEOXYNIVALENOL | PPARG | increases expression | Comparative Toxicogenomics Database | 0.262871983 | -2.458926112 |
| FLAVANONE | PPARG | increases expression | Comparative Toxicogenomics Database | 0.262871983 | -2.458926112 |
| IRON | PPARG | increases expression | Comparative Toxicogenomics Database | 0.262871983 | -2.458926112 |
| atrazine | PPARG | increases expression | Comparative Toxicogenomics Database | 0.262871983 | -2.458926112 |
| 25-Hydroxycholesterol | PPARG | increases expression | Comparative Toxicogenomics Database | 0.262871983 | -2.458926112 |
| ns-398 | PPARG | increases expression | Comparative Toxicogenomics Database | 0.262871983 | -2.458926112 |
| amiodarone | PPARG | increases expression | Comparative Toxicogenomics Database | 0.262871983 | -2.458926112 |
| Dronabinol | PPARG | increases expression | Comparative Toxicogenomics Database | 0.262871983 | -2.458926112 |
| nimesulide | PPARG | increases expression | Comparative Toxicogenomics Database | 0.262871983 | -2.458926112 |
| 2-arachidonoylglycerol | PPARG | increases expression | Comparative Toxicogenomics Database | 0.262871983 | -2.458926112 |
| ciglitazone | PPARG | increases expression | Comparative Toxicogenomics Database | 0.262871983 | -2.458926112 |
| lovastatin | PPARG | increases expression | Comparative Toxicogenomics Database | 0.262871983 | -2.458926112 |
| Pioglitazone | PPARG | increases expression | Comparative Toxicogenomics Database | 0.262871983 | -2.458926112 |
| aspirin | PPARG | increases expression | Comparative Toxicogenomics Database | 0.262871983 | -2.458926112 |
| GW9662 | PPARG | increases expression | Comparative Toxicogenomics Database | 0.262871983 | -2.458926112 |
| 105156-22-7 | PPARG | increases expression | Comparative Toxicogenomics Database | 0.262871983 | -2.458926112 |
| Decitabine | PPARG | increases expression | Comparative Toxicogenomics Database | 0.262871983 | -2.458926112 |
| indomethacin | PPARG | increases expression | Comparative Toxicogenomics Database | 0.262871983 | -2.458926112 |
| dexamethasone | PPARG | increases expression | Comparative Toxicogenomics Database | 0.262871983 | -2.458926112 |
| 13296-76-9 | PPARG | increases expression | Comparative Toxicogenomics Database | 0.262871983 | -2.458926112 |
| celecoxib | PPARG | increases expression | Comparative Toxicogenomics Database | 0.262871983 | -2.458926112 |
| 124020-07-1 | PPARG | increases expression | Comparative Toxicogenomics Database | 0.262871983 | -2.458926112 |
| 1143-70-0 | PPARG | increases expression | Comparative Toxicogenomics Database | 0.262871983 | -2.458926112 |
| estradiol | PPARG | increases expression | Comparative Toxicogenomics Database | 0.262871983 | -2.458926112 |
| IBMX | PPARG | increases expression | Comparative Toxicogenomics Database | 0.262871983 | -2.458926112 |
| 109281-79-0 | PPARG | increases expression | Comparative Toxicogenomics Database | 0.262871983 | -2.458926112 |
| Telmisartan | PPARG | increases expression | Comparative Toxicogenomics Database | 0.262871983 | -2.458926112 |
| Netoglitazone | PPARG | increases expression | Comparative Toxicogenomics Database | 0.262871983 | -2.458926112 |
| gamma-Tocopherol | PPARG | increases expression | Comparative Toxicogenomics Database | 0.262871983 | -2.458926112 |
| rapamycin | PPARG | increases expression | Comparative Toxicogenomics Database | 0.262871983 | -2.458926112 |
| Rifaximin | PPARG | increases expression | Comparative Toxicogenomics Database | 0.262871983 | -2.458926112 |
| resveratrol | PPARG | increases expression | Comparative Toxicogenomics Database | 0.262871983 | -2.458926112 |
| ritonavir | PPARG | increases expression | Comparative Toxicogenomics Database | 0.262871983 | -2.458926112 |
| benzene | PPARG | increases expression | Comparative Toxicogenomics Database | 0.262871983 | -2.458926112 |
| cannabidiol | PPARG | increases expression | Comparative Toxicogenomics Database | 0.262871983 | -2.458926112 |
| benzo[a]pyrene | PPARG | increases expression | Comparative Toxicogenomics Database | 0.262871983 | -2.458926112 |
| gemfibrozil | PPARG | increases expression | Comparative Toxicogenomics Database | 0.262871983 | -2.458926112 |
| troglitazone | PPARG | increases expression | Comparative Toxicogenomics Database | 0.262871983 | -2.458926112 |
| rottlerin | PPARG | increases expression | Comparative Toxicogenomics Database | 0.262871983 | -2.458926112 |
| doxorubicin | PPARG | increases expression | Comparative Toxicogenomics Database | 0.262871983 | -2.458926112 |
| fenofibrate | PPARG | increases expression | Comparative Toxicogenomics Database | 0.262871983 | -2.458926112 |
| nevirapine | PPARG | increases expression | Comparative Toxicogenomics Database | 0.262871983 | -2.458926112 |
| theophylline | PPARG | increases expression | Comparative Toxicogenomics Database | 0.262871983 | -2.458926112 |
| Tetradioxin | PPARG | increases expression | Comparative Toxicogenomics Database | 0.262871983 | -2.458926112 |
| alpha-Tocopherol | PPARG | increases expression | Comparative Toxicogenomics Database | 0.262871983 | -2.458926112 |
| rosiglitazone | PPARG | increases expression | Comparative Toxicogenomics Database | 0.262871983 | -2.458926112 |
| Clorgiline | PREX2 | increases expression | Comparative Toxicogenomics Database | 0.245346045 | -1.232921625 |
| amiodarone | PYGO1 | increases expression | Comparative Toxicogenomics Database | 0.261093348 | -1.899834609 |
| Torcetrapib | PYGO1 | increases expression | Comparative Toxicogenomics Database | 0.261093348 | -1.899834609 |
| quercetin | RAI14 | increases expression | Comparative Toxicogenomics Database | NA | NA |
| acetaminophen | RAI14 | increases expression | Comparative Toxicogenomics Database | NA | NA |
| estradiol | RAI14 | increases expression | Comparative Toxicogenomics Database | NA | NA |
| Tetradioxin | RAI14 | increases expression | Comparative Toxicogenomics Database | NA | NA |
| atrazine | RNF180 | increases expression | Comparative Toxicogenomics Database | 0.213396587 | -2.240294299 |
| etoposide | ROBO3 | increases expression | Comparative Toxicogenomics Database | 0.226316683 | -1.019255702 |
| cytarabine | ROBO3 | increases expression | Comparative Toxicogenomics Database | 0.226316683 | -1.019255702 |
| progesterone | RRM2 | increases expression | Comparative Toxicogenomics Database | NA | NA |
| Enterolactone | RRM2 | increases expression | Comparative Toxicogenomics Database | NA | NA |
| MeIQx | RRM2 | increases expression | Comparative Toxicogenomics Database | NA | NA |
| 5-Fluorouracil | RRM2 | increases expression | Comparative Toxicogenomics Database | NA | NA |
| COBALT | RRM2 | increases expression | Comparative Toxicogenomics Database | NA | NA |
| genistein | RRM2 | increases expression | Comparative Toxicogenomics Database | NA | NA |
| estradiol | RRM2 | increases expression | Comparative Toxicogenomics Database | NA | NA |
| Spectrum_001666 | RRM2 | increases expression | Comparative Toxicogenomics Database | NA | NA |
| Phytoestrogens | RRM2 | increases expression | Comparative Toxicogenomics Database | NA | NA |
| resveratrol | RRM2 | increases expression | Comparative Toxicogenomics Database | NA | NA |
| cytarabine | RRM2 | increases expression | Comparative Toxicogenomics Database | NA | NA |
| COUMESTROL | RRM2 | increases expression | Comparative Toxicogenomics Database | NA | NA |
| Tetradioxin | RRM2 | increases expression | Comparative Toxicogenomics Database | NA | NA |
| N-NITROSODIETHYLAMINE | SDPR | increases expression | Comparative Toxicogenomics Database | 0.23644362 | -1.944536608 |
| benzo[a]pyrene | SDPR | increases expression | Comparative Toxicogenomics Database | 0.23644362 | -1.944536608 |
| Tetradioxin | SDPR | increases expression | Comparative Toxicogenomics Database | 0.23644362 | -1.944536608 |
| 17-Hydroxyandrostan-3-one | SELENBP1 | increases expression | Comparative Toxicogenomics Database | 0.301535021 | -3.38055923 |
| SELENIUM | SELENBP1 | increases expression | Comparative Toxicogenomics Database | 0.301535021 | -3.38055923 |
| FENRETINIDE | SEMA3C | increases expression | Comparative Toxicogenomics Database | NA | NA |
| progesterone | SEMA3C | increases expression | Comparative Toxicogenomics Database | NA | NA |
| 4-Hydroxytamoxifen | SEMA3C | increases expression | Comparative Toxicogenomics Database | NA | NA |
| estradiol | SEMA3C | increases expression | Comparative Toxicogenomics Database | NA | NA |
| Clorgiline | SEMA3C | increases expression | Comparative Toxicogenomics Database | NA | NA |
| rapamycin | SEMA3C | increases expression | Comparative Toxicogenomics Database | NA | NA |
| troglitazone | SEMA3C | increases expression | Comparative Toxicogenomics Database | NA | NA |
| COUMESTROL | SEMA3C | increases expression | Comparative Toxicogenomics Database | NA | NA |
| 5-azacytidine | SEMA6C | increases expression | Comparative Toxicogenomics Database | 0.227478772 | -3.088108768 |
| quercetin | SESN1 | increases expression | Comparative Toxicogenomics Database | 0.35580502 | -1.23118942 |
| etoposide | SESN1 | increases expression | Comparative Toxicogenomics Database | 0.35580502 | -1.23118942 |
| acetaminophen | SESN1 | increases expression | Comparative Toxicogenomics Database | 0.35580502 | -1.23118942 |
| hydroquinone | SESN1 | increases expression | Comparative Toxicogenomics Database | 0.35580502 | -1.23118942 |
| 5-Fluorouracil | SESN1 | increases expression | Comparative Toxicogenomics Database | 0.35580502 | -1.23118942 |
| formaldehyde | SESN1 | increases expression | Comparative Toxicogenomics Database | 0.35580502 | -1.23118942 |
| Demecolcine | SESN1 | increases expression | Comparative Toxicogenomics Database | 0.35580502 | -1.23118942 |
| genistein | SESN1 | increases expression | Comparative Toxicogenomics Database | 0.35580502 | -1.23118942 |
| resveratrol | SESN1 | increases expression | Comparative Toxicogenomics Database | 0.35580502 | -1.23118942 |
| PTAQUILOSIDE | SESN1 | increases expression | Comparative Toxicogenomics Database | 0.35580502 | -1.23118942 |
| benzo[a]pyrene | SESN1 | increases expression | Comparative Toxicogenomics Database | 0.35580502 | -1.23118942 |
| hydroxyurea | SESN1 | increases expression | Comparative Toxicogenomics Database | 0.35580502 | -1.23118942 |
| 7646-79-9 | SESN1 | increases expression | Comparative Toxicogenomics Database | 0.35580502 | -1.23118942 |
| N-NITROSODIETHYLAMINE | SH2D2A | increases expression | Comparative Toxicogenomics Database | NA | NA |
| benzene | SH2D2A | increases expression | Comparative Toxicogenomics Database | NA | NA |
| 3-(1-methylpyrrolidin-2-yl)pyridine | SH2D2A | increases expression | Comparative Toxicogenomics Database | NA | NA |
| benzo[a]pyrene | SLC14A1 | increases expression | Comparative Toxicogenomics Database | 0.26295752 | -2.22839913 |
| cinnamaldehyde | SLC14A1 | increases expression | Comparative Toxicogenomics Database | 0.26295752 | -2.22839913 |
| Tetradioxin | SLC14A1 | increases expression | Comparative Toxicogenomics Database | 0.26295752 | -2.22839913 |
| quercetin | SLC19A3 | increases expression | Comparative Toxicogenomics Database | 0.255208522 | -1.427400802 |
| Cylindrospermopsin | SLC25A25 | increases expression | Comparative Toxicogenomics Database | 0.261270525 | -1.17806749 |
| ZINC | SLC25A25 | increases expression | Comparative Toxicogenomics Database | 0.261270525 | -1.17806749 |
| acetaminophen | SLC25A25 | increases expression | Comparative Toxicogenomics Database | 0.261270525 | -1.17806749 |
| amiodarone | SLC25A25 | increases expression | Comparative Toxicogenomics Database | 0.261270525 | -1.17806749 |
| formaldehyde | SLC25A25 | increases expression | Comparative Toxicogenomics Database | 0.261270525 | -1.17806749 |
| progesterone | SMIM3 | increases expression | Comparative Toxicogenomics Database | NA | NA |
| atrazine | SMIM3 | increases expression | Comparative Toxicogenomics Database | NA | NA |
| calcitriol | SMIM3 | increases expression | Comparative Toxicogenomics Database | NA | NA |
| 5-Fluorouracil | SNTB1 | increases expression | Comparative Toxicogenomics Database | 0.245875939 | -1.170953753 |
| progesterone | SORBS2 | increases expression | Comparative Toxicogenomics Database | 0.260517847 | -2.196978097 |
| Caspan | SORBS2 | increases expression | Comparative Toxicogenomics Database | 0.260517847 | -2.196978097 |
| estradiol | SORBS2 | increases expression | Comparative Toxicogenomics Database | 0.260517847 | -2.196978097 |
| cytarabine | SORBS2 | increases expression | Comparative Toxicogenomics Database | 0.260517847 | -2.196978097 |
| GDC-0941 | SORBS2 | increases expression | Comparative Toxicogenomics Database | 0.260517847 | -2.196978097 |
| Decitabine | SOX17 | increases expression | Comparative Toxicogenomics Database | 0.279422937 | -1.341073912 |
| diethylstilbestrol | SOX17 | increases expression | Comparative Toxicogenomics Database | 0.279422937 | -1.341073912 |
| atrazine | SSBP2 | increases expression | Comparative Toxicogenomics Database | 0.342380863 | -1.26080214 |
| Decitabine | SSBP2 | increases expression | Comparative Toxicogenomics Database | 0.342380863 | -1.26080214 |
| Tetradioxin | SSBP2 | increases expression | Comparative Toxicogenomics Database | 0.342380863 | -1.26080214 |
| LEAD | SSPN | increases expression | Comparative Toxicogenomics Database | 0.236282564 | -1.670515205 |
| hydralazine | SSPN | increases expression | Comparative Toxicogenomics Database | 0.236282564 | -1.670515205 |
| Silica | SULF1 | increases expression | Comparative Toxicogenomics Database | NA | NA |
| Dasatinib | SULF1 | increases expression | Comparative Toxicogenomics Database | NA | NA |
| curcumin | SULF1 | increases expression | Comparative Toxicogenomics Database | NA | NA |
| estradiol | SULF1 | increases expression | Comparative Toxicogenomics Database | NA | NA |
| resveratrol | SULF1 | increases expression | Comparative Toxicogenomics Database | NA | NA |
| GDC-0941 | SULF1 | increases expression | Comparative Toxicogenomics Database | NA | NA |
| COUMESTROL | SULF1 | increases expression | Comparative Toxicogenomics Database | NA | NA |
| Tetradioxin | SULF1 | increases expression | Comparative Toxicogenomics Database | NA | NA |
| estradiol | SUSD4 | increases expression | Comparative Toxicogenomics Database | 0.29995855 | -1.921004984 |
| Tetradioxin | SUSD4 | increases expression | Comparative Toxicogenomics Database | 0.29995855 | -1.921004984 |
| Dasatinib | SVEP1 | increases expression | Comparative Toxicogenomics Database | 0.269380373 | -1.182157726 |
| calcitriol | SVEP1 | increases expression | Comparative Toxicogenomics Database | 0.269380373 | -1.182157726 |
| arsenite | SVEP1 | increases expression | Comparative Toxicogenomics Database | 0.269380373 | -1.182157726 |
| acetaminophen | SVIL | increases expression | Comparative Toxicogenomics Database | 0.237850573 | -1.662557568 |
| amiodarone | SVIL | increases expression | Comparative Toxicogenomics Database | 0.237850573 | -1.662557568 |
| formaldehyde | SVIL | increases expression | Comparative Toxicogenomics Database | 0.237850573 | -1.662557568 |
| mifepristone | SVIL | increases expression | Comparative Toxicogenomics Database | 0.237850573 | -1.662557568 |
| troglitazone | SVIL | increases expression | Comparative Toxicogenomics Database | 0.237850573 | -1.662557568 |
| Torcetrapib | SVIP | increases expression | Comparative Toxicogenomics Database | 0.286370873 | -2.0881152 |
| atrazine | SYNGR1 | increases expression | Comparative Toxicogenomics Database | 0.259284069 | -2.319752277 |
| calcitriol | TBX15 | increases expression | Comparative Toxicogenomics Database | 0.325727623 | -2.905013534 |
| testosterone | TBX15 | increases expression | Comparative Toxicogenomics Database | 0.325727623 | -2.905013534 |
| Dasatinib | TCP11L2 | increases expression | Comparative Toxicogenomics Database | 0.240665678 | -1.398673836 |
| acetaminophen | TCP11L2 | increases expression | Comparative Toxicogenomics Database | 0.240665678 | -1.398673836 |
| COPPER | TCP11L2 | increases expression | Comparative Toxicogenomics Database | 0.240665678 | -1.398673836 |
| benzo[a]pyrene | TCP11L2 | increases expression | Comparative Toxicogenomics Database | 0.240665678 | -1.398673836 |
| LUCANTHONE | TCP11L2 | increases expression | Comparative Toxicogenomics Database | 0.240665678 | -1.398673836 |
| atrazine | TET1 | increases expression | Comparative Toxicogenomics Database | 0.226626917 | -1.948073884 |
| 5-Fluorouracil | TGFBR3 | increases expression | Comparative Toxicogenomics Database | 0.333419504 | -2.494664728 |
| genistein | TGFBR3 | increases expression | Comparative Toxicogenomics Database | 0.333419504 | -2.494664728 |
| estradiol | TGFBR3 | increases expression | Comparative Toxicogenomics Database | 0.333419504 | -2.494664728 |
| benzene | TGFBR3 | increases expression | Comparative Toxicogenomics Database | 0.333419504 | -2.494664728 |
| benzo[a]pyrene | TGFBR3 | increases expression | Comparative Toxicogenomics Database | 0.333419504 | -2.494664728 |
| Vitinoin | THBS4 | increases expression | Comparative Toxicogenomics Database | 0.378819091 | -1.49041776 |
| progesterone | THSD4 | increases expression | Comparative Toxicogenomics Database | 0.303881149 | -1.009731104 |
| 14-Deoxy-11,12-didehydroandrographolide | THSD4 | increases expression | Comparative Toxicogenomics Database | 0.303881149 | -1.009731104 |
| Enterolactone | THSD4 | increases expression | Comparative Toxicogenomics Database | 0.303881149 | -1.009731104 |
| resveratrol | THSD4 | increases expression | Comparative Toxicogenomics Database | 0.303881149 | -1.009731104 |
| COUMESTROL | THSD4 | increases expression | Comparative Toxicogenomics Database | 0.303881149 | -1.009731104 |
| SELENIUM | TMEM63A | increases expression | Comparative Toxicogenomics Database | 0.297579359 | -1.415798984 |
| atrazine | TNS1 | increases expression | Comparative Toxicogenomics Database | 0.269643309 | -1.990325164 |
| ciglitazone | TNS1 | increases expression | Comparative Toxicogenomics Database | 0.269643309 | -1.990325164 |
| resveratrol | TNS1 | increases expression | Comparative Toxicogenomics Database | 0.269643309 | -1.990325164 |
| benzo[a]pyrene | TNS1 | increases expression | Comparative Toxicogenomics Database | 0.269643309 | -1.990325164 |
| monensin | TRPM2 | increases expression | Comparative Toxicogenomics Database | NA | NA |
| LITHIUM | TRPM2 | increases expression | Comparative Toxicogenomics Database | NA | NA |
| amiodarone | WBSCR17 | increases expression | Comparative Toxicogenomics Database | 0.249351227 | -1.265713744 |
| amiodarone | ZBED3 | increases expression | Comparative Toxicogenomics Database | 0.210616383 | -1.534914742 |
| ZINC | ZBP1 | increases expression | Comparative Toxicogenomics Database | NA | NA |
| progesterone | ZBTB16 | increases expression | Comparative Toxicogenomics Database | 0.242756406 | -4.036129768 |
| estradiol | ZBTB16 | increases expression | Comparative Toxicogenomics Database | 0.242756406 | -4.036129768 |
| MERCURY | ZBTB16 | increases expression | Comparative Toxicogenomics Database | 0.242756406 | -4.036129768 |
| benzo[a]pyrene | ZNF132 | increases expression | Comparative Toxicogenomics Database | 0.319150123 | -1.732467417 |
| estradiol | ZNF135 | increases expression | Comparative Toxicogenomics Database | 0.312719395 | -1.908696321 |
| resveratrol | ZNF135 | increases expression | Comparative Toxicogenomics Database | 0.312719395 | -1.908696321 |
| Decitabine | ZNF229 | increases expression | Comparative Toxicogenomics Database | 0.265966925 | -2.408557838 |
| benzo[a]pyrene | ZNF354C | increases expression | Comparative Toxicogenomics Database | 0.222676233 | -1.530591383 |
| hydralazine | ZNF354C | increases expression | Comparative Toxicogenomics Database | 0.222676233 | -1.530591383 |
| acetaminophen | ZNF415 | increases expression | Comparative Toxicogenomics Database | 0.337746547 | -2.145742127 |
| amiodarone | ZNF420 | increases expression | Comparative Toxicogenomics Database | 0.284649336 | -1.66647776 |
| atrazine | ZNF583 | increases expression | Comparative Toxicogenomics Database | 0.348788076 | -1.235413382 |
| Copper_sulfate | ZNF626 | increases expression | Comparative Toxicogenomics Database | 0.241021692 | -2.078230094 |
| CUPRIC_OXIDE | ZNF626 | increases expression | Comparative Toxicogenomics Database | 0.241021692 | -2.078230094 |
| 3-(1-methylpyrrolidin-2-yl)pyridine | ZNF737 | increases expression | Comparative Toxicogenomics Database | 0.268787024 | -1.348761122 |
| acetaminophen | ZNF844 | increases expression | Comparative Toxicogenomics Database | 0.287597446 | -2.215246221 |
| Retinoic_acid | ZNF853 | increases expression | Comparative Toxicogenomics Database | 0.270879824 | -2.059875238 |
| formaldehyde | ZSCAN16 | increases expression | Comparative Toxicogenomics Database | 0.217880033 | -1.118528589 |
| aspirin | ZSCAN16 | increases expression | Comparative Toxicogenomics Database | 0.217880033 | -1.118528589 |
|  |  |  |  |  |  |
| **(B) Drugs that decrease expression** | | | | | |
| **DSigDB database** | | | | **Present Study** | |
| **Drug** | **Gene** | **Type** | **Source** | **Mean_delta_beta** | **Mean_log2(Fold Change)** |
| quercetin | ACSS3 | decreases expression | Comparative Toxicogenomics Database | NA | NA |
| Caspan | ACSS3 | decreases expression | Comparative Toxicogenomics Database | NA | NA |
| acetaminophen | ACSS3 | decreases expression | Comparative Toxicogenomics Database | NA | NA |
| COUMESTROL | ACSS3 | decreases expression | Comparative Toxicogenomics Database | NA | NA |
| testosterone | ACSS3 | decreases expression | Comparative Toxicogenomics Database | NA | NA |
| acetaminophen | ADAM33 | decreases expression | Comparative Toxicogenomics Database | NA | NA |
| benzo[a]pyrene | ADAMTSL1 | decreases expression | Comparative Toxicogenomics Database | NA | NA |
| 7646-79-9 | ADAMTSL1 | decreases expression | Comparative Toxicogenomics Database | NA | NA |
| paclitaxel | ADCY6 | decreases expression | Comparative Toxicogenomics Database | NA | NA |
| cycloheximide | AKAP6 | decreases expression | Comparative Toxicogenomics Database | NA | NA |
| Tetradioxin | AKAP6 | decreases expression | Comparative Toxicogenomics Database | NA | NA |
| MANGANESE | AKAP7 | decreases expression | Comparative Toxicogenomics Database | NA | NA |
| Pirinixic_acid | ALS2CR11 | decreases expression | Comparative Toxicogenomics Database | NA | NA |
| SELENIUM | AMPD3 | decreases expression | Comparative Toxicogenomics Database | -0.231352636 | 1.124229317 |
| quercetin | AOX1 | decreases expression | Comparative Toxicogenomics Database | NA | NA |
| Silica | AOX1 | decreases expression | Comparative Toxicogenomics Database | NA | NA |
| Dasatinib | AOX1 | decreases expression | Comparative Toxicogenomics Database | NA | NA |
| rofecoxib | AOX1 | decreases expression | Comparative Toxicogenomics Database | NA | NA |
| estradiol | AOX1 | decreases expression | Comparative Toxicogenomics Database | NA | NA |
| 3,4-DICHLOROANILINE | AOX1 | decreases expression | Comparative Toxicogenomics Database | NA | NA |
| diuron | AOX1 | decreases expression | Comparative Toxicogenomics Database | NA | NA |
| Tetradioxin | AOX1 | decreases expression | Comparative Toxicogenomics Database | NA | NA |
| atrazine | APBA2 | decreases expression | Comparative Toxicogenomics Database | -0.247858112 | 2.442766967 |
| COPPER | APBA2 | decreases expression | Comparative Toxicogenomics Database | -0.247858112 | 2.442766967 |
| Caspan | BST2 | decreases expression | Comparative Toxicogenomics Database | -0.266760313 | 2.590163177 |
| resveratrol | BST2 | decreases expression | Comparative Toxicogenomics Database | -0.266760313 | 2.590163177 |
| arbutin | BST2 | decreases expression | Comparative Toxicogenomics Database | -0.266760313 | 2.590163177 |
| cytarabine | BST2 | decreases expression | Comparative Toxicogenomics Database | -0.266760313 | 2.590163177 |
| Tetradioxin | BST2 | decreases expression | Comparative Toxicogenomics Database | -0.266760313 | 2.590163177 |
| 3,3',4,4',5-Pentachlorobiphenyl | C1QB | decreases expression | Comparative Toxicogenomics Database | -0.233452807 | 1.195130219 |
| arbutin | C1QB | decreases expression | Comparative Toxicogenomics Database | -0.233452807 | 1.195130219 |
| methotrexate | C1QB | decreases expression | Comparative Toxicogenomics Database | -0.233452807 | 1.195130219 |
| calcitriol | CAB39L | decreases expression | Comparative Toxicogenomics Database | NA | NA |
| estradiol | CAB39L | decreases expression | Comparative Toxicogenomics Database | NA | NA |
| PhIP | CAB39L | decreases expression | Comparative Toxicogenomics Database | NA | NA |
| cytarabine | CAB39L | decreases expression | Comparative Toxicogenomics Database | NA | NA |
| testosterone | CAB39L | decreases expression | Comparative Toxicogenomics Database | NA | NA |
| Silica | CCL24 | decreases expression | Comparative Toxicogenomics Database | -0.210601465 | 2.003483417 |
| 1-NITROPYRENE | CCL24 | decreases expression | Comparative Toxicogenomics Database | -0.210601465 | 2.003483417 |
| Silica | CCR7 | decreases expression | Comparative Toxicogenomics Database | -0.22361721 | 1.46326291 |
| phenobarbital | CCR7 | decreases expression | Comparative Toxicogenomics Database | -0.22361721 | 1.46326291 |
| albendazole | CCR7 | decreases expression | Comparative Toxicogenomics Database | -0.22361721 | 1.46326291 |
| IVERMECTIN | CCR7 | decreases expression | Comparative Toxicogenomics Database | -0.22361721 | 1.46326291 |
| Alitretinoin | CCR7 | decreases expression | Comparative Toxicogenomics Database | -0.22361721 | 1.46326291 |
| 1-NITROPYRENE | CCR7 | decreases expression | Comparative Toxicogenomics Database | -0.22361721 | 1.46326291 |
| rosiglitazone | CCR7 | decreases expression | Comparative Toxicogenomics Database | -0.22361721 | 1.46326291 |
| 1-chloro-2,4-dinitrobenzene | CCR7 | decreases expression | Comparative Toxicogenomics Database | -0.22361721 | 1.46326291 |
| ARSENIC | CCRL2 | decreases expression | Comparative Toxicogenomics Database | -0.222514428 | 1.054464512 |
| valsartan | CD34 | decreases expression | Comparative Toxicogenomics Database | NA | NA |
| FENRETINIDE | CD34 | decreases expression | Comparative Toxicogenomics Database | NA | NA |
| melphalan | CD34 | decreases expression | Comparative Toxicogenomics Database | NA | NA |
| prednisolone | CD34 | decreases expression | Comparative Toxicogenomics Database | NA | NA |
| Monoisoamyl-2,3-dimercaptosuccinate | CD34 | decreases expression | Comparative Toxicogenomics Database | NA | NA |
| aminoguanidine | CD34 | decreases expression | Comparative Toxicogenomics Database | NA | NA |
| O,P'-DDT | CD34 | decreases expression | Comparative Toxicogenomics Database | NA | NA |
| VANADIUM | CD36 | decreases expression | Comparative Toxicogenomics Database | NA | NA |
| Oxazolone | CD36 | decreases expression | Comparative Toxicogenomics Database | NA | NA |
| aspirin | CD36 | decreases expression | Comparative Toxicogenomics Database | NA | NA |
| curcumin | CD36 | decreases expression | Comparative Toxicogenomics Database | NA | NA |
| GW9662 | CD36 | decreases expression | Comparative Toxicogenomics Database | NA | NA |
| eugenol | CD36 | decreases expression | Comparative Toxicogenomics Database | NA | NA |
| estradiol | CD36 | decreases expression | Comparative Toxicogenomics Database | NA | NA |
| mifepristone | CD36 | decreases expression | Comparative Toxicogenomics Database | NA | NA |
| Alitretinoin | CD36 | decreases expression | Comparative Toxicogenomics Database | NA | NA |
| theophylline | CD36 | decreases expression | Comparative Toxicogenomics Database | NA | NA |
| Tetradioxin | CD36 | decreases expression | Comparative Toxicogenomics Database | NA | NA |
| 1-chloro-2,4-dinitrobenzene | CD36 | decreases expression | Comparative Toxicogenomics Database | NA | NA |
| hydrocortisone | CD80 | decreases expression | Comparative Toxicogenomics Database | -0.25288091 | 2.928608949 |
| Dronabinol | CD80 | decreases expression | Comparative Toxicogenomics Database | -0.25288091 | 2.928608949 |
| calcitriol | CD80 | decreases expression | Comparative Toxicogenomics Database | -0.25288091 | 2.928608949 |
| GW9662 | CD80 | decreases expression | Comparative Toxicogenomics Database | -0.25288091 | 2.928608949 |
| AC1L1KON | CD80 | decreases expression | Comparative Toxicogenomics Database | -0.25288091 | 2.928608949 |
| Alitretinoin | CD80 | decreases expression | Comparative Toxicogenomics Database | -0.25288091 | 2.928608949 |
| rosiglitazone | CD80 | decreases expression | Comparative Toxicogenomics Database | -0.25288091 | 2.928608949 |
| Dasatinib | CD274 | decreases expression | Comparative Toxicogenomics Database | -0.241035319 | 2.055991788 |
| carboplatin | CD274 | decreases expression | Comparative Toxicogenomics Database | -0.241035319 | 2.055991788 |
| testosterone | CD274 | decreases expression | Comparative Toxicogenomics Database | -0.241035319 | 2.055991788 |
| quercetin | CGNL1 | decreases expression | Comparative Toxicogenomics Database | NA | NA |
| Caspan | CGNL1 | decreases expression | Comparative Toxicogenomics Database | NA | NA |
| calcitriol | CGNL1 | decreases expression | Comparative Toxicogenomics Database | NA | NA |
| benzo[a]pyrene | CGNL1 | decreases expression | Comparative Toxicogenomics Database | NA | NA |
| 7646-79-9 | CGNL1 | decreases expression | Comparative Toxicogenomics Database | NA | NA |
| Tetradioxin | CGNL1 | decreases expression | Comparative Toxicogenomics Database | NA | NA |
| NICKEL | CHPT1 | decreases expression | Comparative Toxicogenomics Database | NA | NA |
| dmnq | CHPT1 | decreases expression | Comparative Toxicogenomics Database | NA | NA |
| 3-(1-methylpyrrolidin-2-yl)pyridine | CKMT2 | decreases expression | Comparative Toxicogenomics Database | NA | NA |
| COPPER | CLDN11 | decreases expression | Comparative Toxicogenomics Database | NA | NA |
| 3,4-DICHLOROANILINE | CLDN11 | decreases expression | Comparative Toxicogenomics Database | NA | NA |
| diuron | CLDN11 | decreases expression | Comparative Toxicogenomics Database | NA | NA |
| Allococaine | CLDN11 | decreases expression | Comparative Toxicogenomics Database | NA | NA |
| 7646-79-9 | CLDN11 | decreases expression | Comparative Toxicogenomics Database | NA | NA |
| ZINC | COL8A1 | decreases expression | Comparative Toxicogenomics Database | -0.211213079 | 2.140587653 |
| 3,4-DICHLOROANILINE | COL8A1 | decreases expression | Comparative Toxicogenomics Database | -0.211213079 | 2.140587653 |
| progesterone | COX7A1 | decreases expression | Comparative Toxicogenomics Database | NA | NA |
| SELENIUM | COX7A1 | decreases expression | Comparative Toxicogenomics Database | NA | NA |
| 3'-Azido-3'-deoxythymidine | COX7A1 | decreases expression | Comparative Toxicogenomics Database | NA | NA |
| etoposide | CXCL12 | decreases expression | Comparative Toxicogenomics Database | NA | NA |
| Cetrorelix | CXCL12 | decreases expression | Comparative Toxicogenomics Database | NA | NA |
| Dasatinib | CXCL12 | decreases expression | Comparative Toxicogenomics Database | NA | NA |
| progesterone | CXCL12 | decreases expression | Comparative Toxicogenomics Database | NA | NA |
| 14-Deoxy-11,12-didehydroandrographolide | CXCL12 | decreases expression | Comparative Toxicogenomics Database | NA | NA |
| hydroquinone | CXCL12 | decreases expression | Comparative Toxicogenomics Database | NA | NA |
| 17-Hydroxyandrostan-3-one | CXCL12 | decreases expression | Comparative Toxicogenomics Database | NA | NA |
| Cianidanol | CXCL12 | decreases expression | Comparative Toxicogenomics Database | NA | NA |
| tamoxifen | CXCL12 | decreases expression | Comparative Toxicogenomics Database | NA | NA |
| Bicyclam | CXCL12 | decreases expression | Comparative Toxicogenomics Database | NA | NA |
| 2,2',4,4',5,5'-Hexachlorobiphenyl | CXCL12 | decreases expression | Comparative Toxicogenomics Database | NA | NA |
| estradiol | CXCL12 | decreases expression | Comparative Toxicogenomics Database | NA | NA |
| enoxaparin | CXCL12 | decreases expression | Comparative Toxicogenomics Database | NA | NA |
| melphalan | CXCL12 | decreases expression | Comparative Toxicogenomics Database | NA | NA |
| Glyceollin | CXCL12 | decreases expression | Comparative Toxicogenomics Database | NA | NA |
| Tetradioxin | CXCL12 | decreases expression | Comparative Toxicogenomics Database | NA | NA |
| ZINC | CYP2R1 | decreases expression | Comparative Toxicogenomics Database | NA | NA |
| Cianidanol | CYP2R1 | decreases expression | Comparative Toxicogenomics Database | NA | NA |
| arsenite | CYP2R1 | decreases expression | Comparative Toxicogenomics Database | NA | NA |
| acetaminophen | CYP27A1 | decreases expression | Comparative Toxicogenomics Database | NA | NA |
| ACMC-20mvek | CYP27A1 | decreases expression | Comparative Toxicogenomics Database | NA | NA |
| Vitinoin | CYP27A1 | decreases expression | Comparative Toxicogenomics Database | NA | NA |
| GW9662 | CYP27A1 | decreases expression | Comparative Toxicogenomics Database | NA | NA |
| eugenol | CYP27A1 | decreases expression | Comparative Toxicogenomics Database | NA | NA |
| Alitretinoin | CYP27A1 | decreases expression | Comparative Toxicogenomics Database | NA | NA |
| cytarabine | CYP27A1 | decreases expression | Comparative Toxicogenomics Database | NA | NA |
| atrazine | DAGLA | decreases expression | Comparative Toxicogenomics Database | NA | NA |
| estradiol | DLGAP4 | decreases expression | Comparative Toxicogenomics Database | -0.264170355 | 1.114307562 |
| wortmannin | DNMT3B | decreases expression | Comparative Toxicogenomics Database | -0.219911545 | 2.473254423 |
| deferoxamine | DNMT3B | decreases expression | Comparative Toxicogenomics Database | -0.219911545 | 2.473254423 |
| 4-nonylphenol | DNMT3B | decreases expression | Comparative Toxicogenomics Database | -0.219911545 | 2.473254423 |
| 3,3'-Diindolylmethane | DNMT3B | decreases expression | Comparative Toxicogenomics Database | -0.219911545 | 2.473254423 |
| hydroquinone | DNMT3B | decreases expression | Comparative Toxicogenomics Database | -0.219911545 | 2.473254423 |
| calcitriol | DNMT3B | decreases expression | Comparative Toxicogenomics Database | -0.219911545 | 2.473254423 |
| formaldehyde | DNMT3B | decreases expression | Comparative Toxicogenomics Database | -0.219911545 | 2.473254423 |
| Decitabine | DNMT3B | decreases expression | Comparative Toxicogenomics Database | -0.219911545 | 2.473254423 |
| tamoxifen | DNMT3B | decreases expression | Comparative Toxicogenomics Database | -0.219911545 | 2.473254423 |
| 4-tert-Octylphenol | DNMT3B | decreases expression | Comparative Toxicogenomics Database | -0.219911545 | 2.473254423 |
| genistein | DNMT3B | decreases expression | Comparative Toxicogenomics Database | -0.219911545 | 2.473254423 |
| Monoisoamyl-2,3-dimercaptosuccinate | DNMT3B | decreases expression | Comparative Toxicogenomics Database | -0.219911545 | 2.473254423 |
| vanillin | DNMT3B | decreases expression | Comparative Toxicogenomics Database | -0.219911545 | 2.473254423 |
| cinnamaldehyde | DNMT3B | decreases expression | Comparative Toxicogenomics Database | -0.219911545 | 2.473254423 |
| sulforaphane | DNMT3B | decreases expression | Comparative Toxicogenomics Database | -0.219911545 | 2.473254423 |
| Tetradioxin | DNMT3B | decreases expression | Comparative Toxicogenomics Database | -0.219911545 | 2.473254423 |
| testosterone | DNMT3B | decreases expression | Comparative Toxicogenomics Database | -0.219911545 | 2.473254423 |
| CADMIUM | DOK5 | decreases expression | Comparative Toxicogenomics Database | NA | NA |
| Vitinoin | DOPEY2 | decreases expression | Comparative Toxicogenomics Database | NA | NA |
| estradiol | DOPEY2 | decreases expression | Comparative Toxicogenomics Database | NA | NA |
| benzo[a]pyrene | DOPEY2 | decreases expression | Comparative Toxicogenomics Database | NA | NA |
| COUMESTROL | DOPEY2 | decreases expression | Comparative Toxicogenomics Database | NA | NA |
| Oxazolone | DSE | decreases expression | Comparative Toxicogenomics Database | -0.237419518 | 1.202916557 |
| SELENIUM | DSE | decreases expression | Comparative Toxicogenomics Database | -0.237419518 | 1.202916557 |
| benzo[a]pyrene | DSE | decreases expression | Comparative Toxicogenomics Database | -0.237419518 | 1.202916557 |
| 1-chloro-2,4-dinitrobenzene | DSE | decreases expression | Comparative Toxicogenomics Database | -0.237419518 | 1.202916557 |
| atrazine | EBF1 | decreases expression | Comparative Toxicogenomics Database | NA | NA |
| 67526-95-8 | EBF1 | decreases expression | Comparative Toxicogenomics Database | NA | NA |
| melphalan | EBF1 | decreases expression | Comparative Toxicogenomics Database | NA | NA |
| quercetin | EPHX1 | decreases expression | Comparative Toxicogenomics Database | NA | NA |
| PHENCYCLIDINE | EPHX1 | decreases expression | Comparative Toxicogenomics Database | NA | NA |
| carmustine | EPHX1 | decreases expression | Comparative Toxicogenomics Database | NA | NA |
| COPPER | EPHX1 | decreases expression | Comparative Toxicogenomics Database | NA | NA |
| dmnq | EPHX1 | decreases expression | Comparative Toxicogenomics Database | NA | NA |
| 7646-79-9 | EPHX1 | decreases expression | Comparative Toxicogenomics Database | NA | NA |
| quercetin | EPHX2 | decreases expression | Comparative Toxicogenomics Database | NA | NA |
| CHLOROBENZENE | EPHX2 | decreases expression | Comparative Toxicogenomics Database | NA | NA |
| FCCP | EPHX2 | decreases expression | Comparative Toxicogenomics Database | NA | NA |
| INDIRUBIN-3'-MONOXIME | EPHX2 | decreases expression | Comparative Toxicogenomics Database | NA | NA |
| benzo[a]pyrene | EPHX2 | decreases expression | Comparative Toxicogenomics Database | NA | NA |
| Tetradioxin | EPHX2 | decreases expression | Comparative Toxicogenomics Database | NA | NA |
| quercetin | FAM19A5 | decreases expression | Comparative Toxicogenomics Database | NA | NA |
| benzo[a]pyrene | FAM19A5 | decreases expression | Comparative Toxicogenomics Database | NA | NA |
| estradiol | FAM47E | decreases expression | Comparative Toxicogenomics Database | NA | NA |
| arsenite | FAM171B | decreases expression | Comparative Toxicogenomics Database | NA | NA |
| vincristine | FAM171B | decreases expression | Comparative Toxicogenomics Database | NA | NA |
| liothyronine | FAM171B | decreases expression | Comparative Toxicogenomics Database | NA | NA |
| resveratrol | FAM171B | decreases expression | Comparative Toxicogenomics Database | NA | NA |
| COUMESTROL | FAM171B | decreases expression | Comparative Toxicogenomics Database | NA | NA |
| Dasatinib | FAM180A | decreases expression | Comparative Toxicogenomics Database | NA | NA |
| methotrexate | FGF12 | decreases expression | Comparative Toxicogenomics Database | NA | NA |
| Bortezomib | GAS7 | decreases expression | Comparative Toxicogenomics Database | NA | NA |
| benzo[a]pyrene | GAS7 | decreases expression | Comparative Toxicogenomics Database | NA | NA |
| estradiol | GFRA1 | decreases expression | Comparative Toxicogenomics Database | NA | NA |
| Tetradioxin | GFRA1 | decreases expression | Comparative Toxicogenomics Database | NA | NA |
| Tetradioxin | GPR153 | decreases expression | Comparative Toxicogenomics Database | -0.241427077 | 1.813895203 |
| CADMIUM | GPX3 | decreases expression | Comparative Toxicogenomics Database | NA | NA |
| 1,10-phenanthroline | GPX3 | decreases expression | Comparative Toxicogenomics Database | NA | NA |
| Vitinoin | GPX3 | decreases expression | Comparative Toxicogenomics Database | NA | NA |
| Caspan | GSN | decreases expression | Comparative Toxicogenomics Database | NA | NA |
| Vorinostat | GSN | decreases expression | Comparative Toxicogenomics Database | NA | NA |
| paclitaxel | GSN | decreases expression | Comparative Toxicogenomics Database | NA | NA |
| Chlorophenothane | GSN | decreases expression | Comparative Toxicogenomics Database | NA | NA |
| CADMIUM | GSN | decreases expression | Comparative Toxicogenomics Database | NA | NA |
| tetrahydropalmatine | GSN | decreases expression | Comparative Toxicogenomics Database | NA | NA |
| Decitabine | GSN | decreases expression | Comparative Toxicogenomics Database | NA | NA |
| N-NITROSODIMETHYLAMINE | GSN | decreases expression | Comparative Toxicogenomics Database | NA | NA |
| estradiol | GSN | decreases expression | Comparative Toxicogenomics Database | NA | NA |
| dmnq | GSN | decreases expression | Comparative Toxicogenomics Database | NA | NA |
| MERCURY | GSN | decreases expression | Comparative Toxicogenomics Database | NA | NA |
| troglitazone | GSN | decreases expression | Comparative Toxicogenomics Database | NA | NA |
| 635-65-4 | GSN | decreases expression | Comparative Toxicogenomics Database | NA | NA |
| Allococaine | GSN | decreases expression | Comparative Toxicogenomics Database | NA | NA |
| 7646-79-9 | GSN | decreases expression | Comparative Toxicogenomics Database | NA | NA |
| tamoxifen | GZMB | decreases expression | Comparative Toxicogenomics Database | -0.248153201 | 2.386697046 |
| rapamycin | GZMB | decreases expression | Comparative Toxicogenomics Database | -0.248153201 | 2.386697046 |
| Fulvestrant | GZMB | decreases expression | Comparative Toxicogenomics Database | -0.248153201 | 2.386697046 |
| dichlorvos | GZMB | decreases expression | Comparative Toxicogenomics Database | -0.248153201 | 2.386697046 |
| ZIRAM | GZMB | decreases expression | Comparative Toxicogenomics Database | -0.248153201 | 2.386697046 |
| Silica | HAVCR2 | decreases expression | Comparative Toxicogenomics Database | -0.279637155 | 1.513793044 |
| benzo[a]pyrene | HAVCR2 | decreases expression | Comparative Toxicogenomics Database | -0.279637155 | 1.513793044 |
| progesterone | ID4 | decreases expression | Comparative Toxicogenomics Database | NA | NA |
| estradiol | ID4 | decreases expression | Comparative Toxicogenomics Database | NA | NA |
| 7646-79-9 | ID4 | decreases expression | Comparative Toxicogenomics Database | NA | NA |
| Tetradioxin | ID4 | decreases expression | Comparative Toxicogenomics Database | NA | NA |
| Bortezomib | IFITM1 | decreases expression | Comparative Toxicogenomics Database | -0.201877333 | 1.360989111 |
| Glycidamide | IFITM1 | decreases expression | Comparative Toxicogenomics Database | -0.201877333 | 1.360989111 |
| acetaminophen | IFITM1 | decreases expression | Comparative Toxicogenomics Database | -0.201877333 | 1.360989111 |
| estradiol | IFITM1 | decreases expression | Comparative Toxicogenomics Database | -0.201877333 | 1.360989111 |
| 1-NITROPYRENE | IFITM1 | decreases expression | Comparative Toxicogenomics Database | -0.201877333 | 1.360989111 |
| troglitazone | IFITM1 | decreases expression | Comparative Toxicogenomics Database | -0.201877333 | 1.360989111 |
| Tetradioxin | IFITM1 | decreases expression | Comparative Toxicogenomics Database | -0.201877333 | 1.360989111 |
| 7646-79-9 | IGF2BP2 | decreases expression | Comparative Toxicogenomics Database | -0.2575955 | 2.570319525 |
| 67526-95-8 | IL21R | decreases expression | Comparative Toxicogenomics Database | -0.247728976 | 2.059847413 |
| estradiol | ITIH5 | decreases expression | Comparative Toxicogenomics Database | NA | NA |
| Enterolactone | KIF5C | decreases expression | Comparative Toxicogenomics Database | NA | NA |
| SELENIUM | KIF5C | decreases expression | Comparative Toxicogenomics Database | NA | NA |
| COPPER | KIF5C | decreases expression | Comparative Toxicogenomics Database | NA | NA |
| COUMESTROL | KIF5C | decreases expression | Comparative Toxicogenomics Database | NA | NA |
| progesterone | KLHL23 | decreases expression | Comparative Toxicogenomics Database | NA | NA |
| ZINC | KLHL23 | decreases expression | Comparative Toxicogenomics Database | NA | NA |
| formaldehyde | KLHL23 | decreases expression | Comparative Toxicogenomics Database | NA | NA |
| Demecolcine | KLHL23 | decreases expression | Comparative Toxicogenomics Database | NA | NA |
| vincristine | KLHL23 | decreases expression | Comparative Toxicogenomics Database | NA | NA |
| estradiol | KLHL23 | decreases expression | Comparative Toxicogenomics Database | NA | NA |
| oxygen | KLHL23 | decreases expression | Comparative Toxicogenomics Database | NA | NA |
| Tetradioxin | KLHL23 | decreases expression | Comparative Toxicogenomics Database | NA | NA |
| benzo[a]pyrene | L3MBTL4 | decreases expression | Comparative Toxicogenomics Database | NA | NA |
| LEAD | LAIR1 | decreases expression | Comparative Toxicogenomics Database | -0.235320914 | 1.221136315 |
| benzo[a]pyrene | LAIR1 | decreases expression | Comparative Toxicogenomics Database | -0.235320914 | 1.221136315 |
| progesterone | LAMA3 | decreases expression | Comparative Toxicogenomics Database | -0.288786974 | 2.834475229 |
| formaldehyde | LAMA3 | decreases expression | Comparative Toxicogenomics Database | -0.288786974 | 2.834475229 |
| Vitinoin | LAMA3 | decreases expression | Comparative Toxicogenomics Database | -0.288786974 | 2.834475229 |
| estradiol | LAMA3 | decreases expression | Comparative Toxicogenomics Database | -0.288786974 | 2.834475229 |
| mifepristone | LAMA3 | decreases expression | Comparative Toxicogenomics Database | -0.288786974 | 2.834475229 |
| Alitretinoin | LAMA3 | decreases expression | Comparative Toxicogenomics Database | -0.288786974 | 2.834475229 |
| Tetradioxin | LAMA3 | decreases expression | Comparative Toxicogenomics Database | -0.288786974 | 2.834475229 |
| progesterone | LCK | decreases expression | Comparative Toxicogenomics Database | -0.277134562 | 1.174867571 |
| aspirin | LCK | decreases expression | Comparative Toxicogenomics Database | -0.277134562 | 1.174867571 |
| 4-Hydroxytamoxifen | LCK | decreases expression | Comparative Toxicogenomics Database | -0.277134562 | 1.174867571 |
| estradiol | LCK | decreases expression | Comparative Toxicogenomics Database | -0.277134562 | 1.174867571 |
| 1,1-Bis(4-hydroxyphenyl)-2-phenylbut-1-ene | LCK | decreases expression | Comparative Toxicogenomics Database | -0.277134562 | 1.174867571 |
| Cianidanol | LILRB1 | decreases expression | Comparative Toxicogenomics Database | -0.233285331 | 1.775144986 |
| vincristine | LILRB1 | decreases expression | Comparative Toxicogenomics Database | -0.233285331 | 1.775144986 |
| benzo[a]pyrene | LILRB1 | decreases expression | Comparative Toxicogenomics Database | -0.233285331 | 1.775144986 |
| methotrexate | LILRB1 | decreases expression | Comparative Toxicogenomics Database | -0.233285331 | 1.775144986 |
| Tetradioxin | LILRB1 | decreases expression | Comparative Toxicogenomics Database | -0.233285331 | 1.775144986 |
| Bortezomib | LRFN4 | decreases expression | Comparative Toxicogenomics Database | -0.254994197 | 1.645994892 |
| parthenolide | LTC4S | decreases expression | Comparative Toxicogenomics Database | NA | NA |
| quercetin | MAGI2 | decreases expression | Comparative Toxicogenomics Database | NA | NA |
| acetaminophen | MAGI2 | decreases expression | Comparative Toxicogenomics Database | NA | NA |
| COBALT | MAGI2 | decreases expression | Comparative Toxicogenomics Database | NA | NA |
| SELENIUM | MAGI2 | decreases expression | Comparative Toxicogenomics Database | NA | NA |
| benzo[a]pyrene | MAGI2 | decreases expression | Comparative Toxicogenomics Database | NA | NA |
| progesterone | MFAP2 | decreases expression | Comparative Toxicogenomics Database | -0.219811925 | 3.0201541 |
| Vitinoin | MFAP2 | decreases expression | Comparative Toxicogenomics Database | -0.219811925 | 3.0201541 |
| arsenite | MFAP2 | decreases expression | Comparative Toxicogenomics Database | -0.219811925 | 3.0201541 |
| 5-azacytidine | MFAP2 | decreases expression | Comparative Toxicogenomics Database | -0.219811925 | 3.0201541 |
| estradiol | MFAP2 | decreases expression | Comparative Toxicogenomics Database | -0.219811925 | 3.0201541 |
| Alitretinoin | MFAP2 | decreases expression | Comparative Toxicogenomics Database | -0.219811925 | 3.0201541 |
| EMBELIN | MKI67 | decreases expression | Comparative Toxicogenomics Database | -0.232401591 | 1.740293425 |
| 3-Butylidenephthalide | MKI67 | decreases expression | Comparative Toxicogenomics Database | -0.232401591 | 1.740293425 |
| Dasatinib | MKI67 | decreases expression | Comparative Toxicogenomics Database | -0.232401591 | 1.740293425 |
| ABT-737 | MKI67 | decreases expression | Comparative Toxicogenomics Database | -0.232401591 | 1.740293425 |
| Dibenzo[def,p]chrysene | MKI67 | decreases expression | Comparative Toxicogenomics Database | -0.232401591 | 1.740293425 |
| 7-Hydroxystaurosporine | MKI67 | decreases expression | Comparative Toxicogenomics Database | -0.232401591 | 1.740293425 |
| Bortezomib | MKI67 | decreases expression | Comparative Toxicogenomics Database | -0.232401591 | 1.740293425 |
| Paraoxon-methyl | MKI67 | decreases expression | Comparative Toxicogenomics Database | -0.232401591 | 1.740293425 |
| CI-1040 | MKI67 | decreases expression | Comparative Toxicogenomics Database | -0.232401591 | 1.740293425 |
| 5-Fluorouracil | MKI67 | decreases expression | Comparative Toxicogenomics Database | -0.232401591 | 1.740293425 |
| bicalutamide | MKI67 | decreases expression | Comparative Toxicogenomics Database | -0.232401591 | 1.740293425 |
| Deguelin | MKI67 | decreases expression | Comparative Toxicogenomics Database | -0.232401591 | 1.740293425 |
| calcitriol | MKI67 | decreases expression | Comparative Toxicogenomics Database | -0.232401591 | 1.740293425 |
| EXEMESTANE | MKI67 | decreases expression | Comparative Toxicogenomics Database | -0.232401591 | 1.740293425 |
| curcumin | MKI67 | decreases expression | Comparative Toxicogenomics Database | -0.232401591 | 1.740293425 |
| 67526-95-8 | MKI67 | decreases expression | Comparative Toxicogenomics Database | -0.232401591 | 1.740293425 |
| Melatonin | MKI67 | decreases expression | Comparative Toxicogenomics Database | -0.232401591 | 1.740293425 |
| tamoxifen | MKI67 | decreases expression | Comparative Toxicogenomics Database | -0.232401591 | 1.740293425 |
| dexamethasone | MKI67 | decreases expression | Comparative Toxicogenomics Database | -0.232401591 | 1.740293425 |
| celecoxib | MKI67 | decreases expression | Comparative Toxicogenomics Database | -0.232401591 | 1.740293425 |
| piroxicam | MKI67 | decreases expression | Comparative Toxicogenomics Database | -0.232401591 | 1.740293425 |
| COPPER | MKI67 | decreases expression | Comparative Toxicogenomics Database | -0.232401591 | 1.740293425 |
| mifepristone | MKI67 | decreases expression | Comparative Toxicogenomics Database | -0.232401591 | 1.740293425 |
| rapamycin | MKI67 | decreases expression | Comparative Toxicogenomics Database | -0.232401591 | 1.740293425 |
| norgestrel | MKI67 | decreases expression | Comparative Toxicogenomics Database | -0.232401591 | 1.740293425 |
| resveratrol | MKI67 | decreases expression | Comparative Toxicogenomics Database | -0.232401591 | 1.740293425 |
| ACRYLAMIDE | MKI67 | decreases expression | Comparative Toxicogenomics Database | -0.232401591 | 1.740293425 |
| benzene | MKI67 | decreases expression | Comparative Toxicogenomics Database | -0.232401591 | 1.740293425 |
| raloxifene | MKI67 | decreases expression | Comparative Toxicogenomics Database | -0.232401591 | 1.740293425 |
| troglitazone | MKI67 | decreases expression | Comparative Toxicogenomics Database | -0.232401591 | 1.740293425 |
| aminoguanidine | MKI67 | decreases expression | Comparative Toxicogenomics Database | -0.232401591 | 1.740293425 |
| LUCANTHONE | MKI67 | decreases expression | Comparative Toxicogenomics Database | -0.232401591 | 1.740293425 |
| doxorubicin | MKI67 | decreases expression | Comparative Toxicogenomics Database | -0.232401591 | 1.740293425 |
| 7646-79-9 | MKI67 | decreases expression | Comparative Toxicogenomics Database | -0.232401591 | 1.740293425 |
| letrozole | MKI67 | decreases expression | Comparative Toxicogenomics Database | -0.232401591 | 1.740293425 |
| anastrozole | MKI67 | decreases expression | Comparative Toxicogenomics Database | -0.232401591 | 1.740293425 |
| rosiglitazone | MKI67 | decreases expression | Comparative Toxicogenomics Database | -0.232401591 | 1.740293425 |
| chlorpromazine | MKI67 | decreases expression | Comparative Toxicogenomics Database | -0.232401591 | 1.740293425 |
| testosterone | MKI67 | decreases expression | Comparative Toxicogenomics Database | -0.232401591 | 1.740293425 |
| N-NITROSODIETHYLAMINE | MLPH | decreases expression | Comparative Toxicogenomics Database | NA | NA |
| CADMIUM | MMP13 | decreases expression | Comparative Toxicogenomics Database | -0.223340619 | 9.157248179 |
| norfloxacin | MMP13 | decreases expression | Comparative Toxicogenomics Database | -0.223340619 | 9.157248179 |
| Dieckol | MMP13 | decreases expression | Comparative Toxicogenomics Database | -0.223340619 | 9.157248179 |
| Vitinoin | MMP13 | decreases expression | Comparative Toxicogenomics Database | -0.223340619 | 9.157248179 |
| oxygen | MMP13 | decreases expression | Comparative Toxicogenomics Database | -0.223340619 | 9.157248179 |
| COPPER | MMP13 | decreases expression | Comparative Toxicogenomics Database | -0.223340619 | 9.157248179 |
| ciprofloxacin | MMP13 | decreases expression | Comparative Toxicogenomics Database | -0.223340619 | 9.157248179 |
| benzo[a]pyrene | MMP13 | decreases expression | Comparative Toxicogenomics Database | -0.223340619 | 9.157248179 |
| ofloxacin | MMP13 | decreases expression | Comparative Toxicogenomics Database | -0.223340619 | 9.157248179 |
| cyclophosphamide | MX2 | decreases expression | Comparative Toxicogenomics Database | -0.258926759 | 1.8743868 |
| formaldehyde | MYEF2 | decreases expression | Comparative Toxicogenomics Database | NA | NA |
| Decitabine | MYEF2 | decreases expression | Comparative Toxicogenomics Database | NA | NA |
| 7646-79-9 | MYEF2 | decreases expression | Comparative Toxicogenomics Database | NA | NA |
| ZINC | MYH14 | decreases expression | Comparative Toxicogenomics Database | NA | NA |
| ARSENIC | MYH14 | decreases expression | Comparative Toxicogenomics Database | NA | NA |
| resveratrol | MYH14 | decreases expression | Comparative Toxicogenomics Database | NA | NA |
| COUMESTROL | MYH14 | decreases expression | Comparative Toxicogenomics Database | NA | NA |
| atrazine | NCAM2 | decreases expression | Comparative Toxicogenomics Database | NA | NA |
| estradiol | NCAM2 | decreases expression | Comparative Toxicogenomics Database | NA | NA |
| quercetin | NDRG2 | decreases expression | Comparative Toxicogenomics Database | NA | NA |
| progesterone | NDRG2 | decreases expression | Comparative Toxicogenomics Database | NA | NA |
| N-NITROSODIMETHYLAMINE | NDRG2 | decreases expression | Comparative Toxicogenomics Database | NA | NA |
| benzo[a]pyrene | NDRG2 | decreases expression | Comparative Toxicogenomics Database | NA | NA |
| AM580 | NDRG2 | decreases expression | Comparative Toxicogenomics Database | NA | NA |
| ANTIMONY | NDRG2 | decreases expression | Comparative Toxicogenomics Database | NA | NA |
| cytarabine | NDRG2 | decreases expression | Comparative Toxicogenomics Database | NA | NA |
| Allococaine | NDRG2 | decreases expression | Comparative Toxicogenomics Database | NA | NA |
| rosiglitazone | NDRG2 | decreases expression | Comparative Toxicogenomics Database | NA | NA |
| MANGANESE | NFIX | decreases expression | Comparative Toxicogenomics Database | NA | NA |
| Decitabine | NFIX | decreases expression | Comparative Toxicogenomics Database | NA | NA |
| tamoxifen | NFIX | decreases expression | Comparative Toxicogenomics Database | NA | NA |
| quercetin | NMI | decreases expression | Comparative Toxicogenomics Database | -0.328397181 | 1.483184849 |
| acetaminophen | NMI | decreases expression | Comparative Toxicogenomics Database | -0.328397181 | 1.483184849 |
| progesterone | NR3C2 | decreases expression | Comparative Toxicogenomics Database | NA | NA |
| fludrocortisone | NR3C2 | decreases expression | Comparative Toxicogenomics Database | NA | NA |
| ALDOSTERONE | NR3C2 | decreases expression | Comparative Toxicogenomics Database | NA | NA |
| estradiol | NR3C2 | decreases expression | Comparative Toxicogenomics Database | NA | NA |
| benzo[a]pyrene | NR3C2 | decreases expression | Comparative Toxicogenomics Database | NA | NA |
| 7646-79-9 | NR3C2 | decreases expression | Comparative Toxicogenomics Database | NA | NA |
| Silica | NRIP2 | decreases expression | Comparative Toxicogenomics Database | NA | NA |
| Silica | NTRK3 | decreases expression | Comparative Toxicogenomics Database | NA | NA |
| PHENCYCLIDINE | NTRK3 | decreases expression | Comparative Toxicogenomics Database | NA | NA |
| calcitriol | NTRK3 | decreases expression | Comparative Toxicogenomics Database | NA | NA |
| benzo[a]pyrene | NTRK3 | decreases expression | Comparative Toxicogenomics Database | NA | NA |
| CADMIUM | NUPR1 | decreases expression | Comparative Toxicogenomics Database | NA | NA |
| Dronabinol | NUPR1 | decreases expression | Comparative Toxicogenomics Database | NA | NA |
| N-NITROSODIETHYLAMINE | NUPR1 | decreases expression | Comparative Toxicogenomics Database | NA | NA |
| estradiol | NUPR1 | decreases expression | Comparative Toxicogenomics Database | NA | NA |
| erythromycin | NUPR1 | decreases expression | Comparative Toxicogenomics Database | NA | NA |
| gemcitabine | NUPR1 | decreases expression | Comparative Toxicogenomics Database | NA | NA |
| estradiol | OAS2 | decreases expression | Comparative Toxicogenomics Database | -0.225451319 | 2.352991565 |
| Tetradioxin | OAS2 | decreases expression | Comparative Toxicogenomics Database | -0.225451319 | 2.352991565 |
| calcitriol | OLR1 | decreases expression | Comparative Toxicogenomics Database | -0.226535507 | 2.398094348 |
| Decitabine | OLR1 | decreases expression | Comparative Toxicogenomics Database | -0.226535507 | 2.398094348 |
| SARIN | OLR1 | decreases expression | Comparative Toxicogenomics Database | -0.226535507 | 2.398094348 |
| nifedipine | OLR1 | decreases expression | Comparative Toxicogenomics Database | -0.226535507 | 2.398094348 |
| cytarabine | OLR1 | decreases expression | Comparative Toxicogenomics Database | -0.226535507 | 2.398094348 |
| 7646-79-9 | OLR1 | decreases expression | Comparative Toxicogenomics Database | -0.226535507 | 2.398094348 |
| SELENIUM | OSBPL1A | decreases expression | Comparative Toxicogenomics Database | NA | NA |
| Allococaine | OSBPL1A | decreases expression | Comparative Toxicogenomics Database | NA | NA |
| Tetradioxin | OSBPL1A | decreases expression | Comparative Toxicogenomics Database | NA | NA |
| cytarabine | OSR1 | decreases expression | Comparative Toxicogenomics Database | NA | NA |
| ZINC | P2RY6 | decreases expression | Comparative Toxicogenomics Database | -0.213298498 | 1.816031429 |
| cytarabine | PAMR1 | decreases expression | Comparative Toxicogenomics Database | NA | NA |
| carmustine | PCYT1A | decreases expression | Comparative Toxicogenomics Database | -0.229051159 | 1.033375774 |
| Caspan | PDK4 | decreases expression | Comparative Toxicogenomics Database | NA | NA |
| estradiol | PDK4 | decreases expression | Comparative Toxicogenomics Database | NA | NA |
| benzo[a]pyrene | PDK4 | decreases expression | Comparative Toxicogenomics Database | NA | NA |
| diuron | PDK4 | decreases expression | Comparative Toxicogenomics Database | NA | NA |
| Tetradioxin | PDK4 | decreases expression | Comparative Toxicogenomics Database | NA | NA |
| CADMIUM | PER2 | decreases expression | Comparative Toxicogenomics Database | NA | NA |
| acetaminophen | PER2 | decreases expression | Comparative Toxicogenomics Database | NA | NA |
| formaldehyde | PER2 | decreases expression | Comparative Toxicogenomics Database | NA | NA |
| Bexarotene | PER2 | decreases expression | Comparative Toxicogenomics Database | NA | NA |
| prednisolone | PER2 | decreases expression | Comparative Toxicogenomics Database | NA | NA |
| diuron | PER2 | decreases expression | Comparative Toxicogenomics Database | NA | NA |
| benzo[a]pyrene | PGAP3 | decreases expression | Comparative Toxicogenomics Database | NA | NA |
| quercetin | PGM1 | decreases expression | Comparative Toxicogenomics Database | NA | NA |
| Caspan | PGM1 | decreases expression | Comparative Toxicogenomics Database | NA | NA |
| acetaminophen | PGM1 | decreases expression | Comparative Toxicogenomics Database | NA | NA |
| 7,8-Benzoflavone | PGM1 | decreases expression | Comparative Toxicogenomics Database | NA | NA |
| formaldehyde | PGM1 | decreases expression | Comparative Toxicogenomics Database | NA | NA |
| Vitinoin | PGM1 | decreases expression | Comparative Toxicogenomics Database | NA | NA |
| Demecolcine | PGM1 | decreases expression | Comparative Toxicogenomics Database | NA | NA |
| vincristine | PGM1 | decreases expression | Comparative Toxicogenomics Database | NA | NA |
| Tetradioxin | PGM1 | decreases expression | Comparative Toxicogenomics Database | NA | NA |
| hydralazine | PHC1 | decreases expression | Comparative Toxicogenomics Database | NA | NA |
| quercetin | PHYHD1 | decreases expression | Comparative Toxicogenomics Database | NA | NA |
| Tetradioxin | PHYHD1 | decreases expression | Comparative Toxicogenomics Database | NA | NA |
| Rosuvastatin | PI3 | decreases expression | Comparative Toxicogenomics Database | -0.216544332 | 1.124295897 |
| gedunin | PIK3CD | decreases expression | Comparative Toxicogenomics Database | -0.26086583 | 1.271543176 |
| celastrol | PIK3CD | decreases expression | Comparative Toxicogenomics Database | -0.26086583 | 1.271543176 |
| benzo[a]pyrene | PIK3CD | decreases expression | Comparative Toxicogenomics Database | -0.26086583 | 1.271543176 |
| Caspan | PITX1 | decreases expression | Comparative Toxicogenomics Database | NA | NA |
| cytarabine | PITX1 | decreases expression | Comparative Toxicogenomics Database | NA | NA |
| Andriol | PLAGL1 | decreases expression | Comparative Toxicogenomics Database | NA | NA |
| norgestrel | PLAGL1 | decreases expression | Comparative Toxicogenomics Database | NA | NA |
| progesterone | PMEPA1 | decreases expression | Comparative Toxicogenomics Database | -0.249387946 | 1.53509695 |
| Enterolactone | PMEPA1 | decreases expression | Comparative Toxicogenomics Database | -0.249387946 | 1.53509695 |
| Difenoconazole | PMEPA1 | decreases expression | Comparative Toxicogenomics Database | -0.249387946 | 1.53509695 |
| atrazine | PMEPA1 | decreases expression | Comparative Toxicogenomics Database | -0.249387946 | 1.53509695 |
| Diacetylmorphine | PMEPA1 | decreases expression | Comparative Toxicogenomics Database | -0.249387946 | 1.53509695 |
| curcumin | PMEPA1 | decreases expression | Comparative Toxicogenomics Database | -0.249387946 | 1.53509695 |
| CHROMIUM | PMEPA1 | decreases expression | Comparative Toxicogenomics Database | -0.249387946 | 1.53509695 |
| genistein | PMEPA1 | decreases expression | Comparative Toxicogenomics Database | -0.249387946 | 1.53509695 |
| estradiol | PMEPA1 | decreases expression | Comparative Toxicogenomics Database | -0.249387946 | 1.53509695 |
| resveratrol | PMEPA1 | decreases expression | Comparative Toxicogenomics Database | -0.249387946 | 1.53509695 |
| GDC-0941 | PMEPA1 | decreases expression | Comparative Toxicogenomics Database | -0.249387946 | 1.53509695 |
| COUMESTROL | PMEPA1 | decreases expression | Comparative Toxicogenomics Database | -0.249387946 | 1.53509695 |
| 7646-79-9 | PMEPA1 | decreases expression | Comparative Toxicogenomics Database | -0.249387946 | 1.53509695 |
| benzo[a]pyrene | PODN | decreases expression | Comparative Toxicogenomics Database | NA | NA |
| bezafibrate | PPARG | decreases expression | Comparative Toxicogenomics Database | NA | NA |
| valsartan | PPARG | decreases expression | Comparative Toxicogenomics Database | NA | NA |
| pomiferin | PPARG | decreases expression | Comparative Toxicogenomics Database | NA | NA |
| Mehp | PPARG | decreases expression | Comparative Toxicogenomics Database | NA | NA |
| simvastatin | PPARG | decreases expression | Comparative Toxicogenomics Database | NA | NA |
| metformin | PPARG | decreases expression | Comparative Toxicogenomics Database | NA | NA |
| ARSENIC | PPARG | decreases expression | Comparative Toxicogenomics Database | NA | NA |
| Honokiol | PPARG | decreases expression | Comparative Toxicogenomics Database | NA | NA |
| 5-Fluorouracil | PPARG | decreases expression | Comparative Toxicogenomics Database | NA | NA |
| ns-398 | PPARG | decreases expression | Comparative Toxicogenomics Database | NA | NA |
| Puerarin | PPARG | decreases expression | Comparative Toxicogenomics Database | NA | NA |
| nimesulide | PPARG | decreases expression | Comparative Toxicogenomics Database | NA | NA |
| 17-Hydroxyandrostan-3-one | PPARG | decreases expression | Comparative Toxicogenomics Database | NA | NA |
| ciglitazone | PPARG | decreases expression | Comparative Toxicogenomics Database | NA | NA |
| rosavin | PPARG | decreases expression | Comparative Toxicogenomics Database | NA | NA |
| curcumin | PPARG | decreases expression | Comparative Toxicogenomics Database | NA | NA |
| GW9662 | PPARG | decreases expression | Comparative Toxicogenomics Database | NA | NA |
| Melatonin | PPARG | decreases expression | Comparative Toxicogenomics Database | NA | NA |
| stavudine | PPARG | decreases expression | Comparative Toxicogenomics Database | NA | NA |
| indomethacin | PPARG | decreases expression | Comparative Toxicogenomics Database | NA | NA |
| celecoxib | PPARG | decreases expression | Comparative Toxicogenomics Database | NA | NA |
| genistein | PPARG | decreases expression | Comparative Toxicogenomics Database | NA | NA |
| estradiol | PPARG | decreases expression | Comparative Toxicogenomics Database | NA | NA |
| oxygen | PPARG | decreases expression | Comparative Toxicogenomics Database | NA | NA |
| Osajin | PPARG | decreases expression | Comparative Toxicogenomics Database | NA | NA |
| rapamycin | PPARG | decreases expression | Comparative Toxicogenomics Database | NA | NA |
| resveratrol | PPARG | decreases expression | Comparative Toxicogenomics Database | NA | NA |
| chlorpyrifos | PPARG | decreases expression | Comparative Toxicogenomics Database | NA | NA |
| isoniazid | PPARG | decreases expression | Comparative Toxicogenomics Database | NA | NA |
| 2,6-Di-tert-butyl-4-methylphenol | PPARG | decreases expression | Comparative Toxicogenomics Database | NA | NA |
| benzo[a]pyrene | PPARG | decreases expression | Comparative Toxicogenomics Database | NA | NA |
| troglitazone | PPARG | decreases expression | Comparative Toxicogenomics Database | NA | NA |
| cholesterol | PPARG | decreases expression | Comparative Toxicogenomics Database | NA | NA |
| Capsaicin | PPARG | decreases expression | Comparative Toxicogenomics Database | NA | NA |
| 7646-79-9 | PPARG | decreases expression | Comparative Toxicogenomics Database | NA | NA |
| rosiglitazone | PPARG | decreases expression | Comparative Toxicogenomics Database | NA | NA |
| ursodiol | PPARG | decreases expression | Comparative Toxicogenomics Database | NA | NA |
| testosterone | PPARG | decreases expression | Comparative Toxicogenomics Database | NA | NA |
| venlafaxine | PREX2 | decreases expression | Comparative Toxicogenomics Database | NA | NA |
| 7646-79-9 | PREX2 | decreases expression | Comparative Toxicogenomics Database | NA | NA |
| ZINC | PYGO1 | decreases expression | Comparative Toxicogenomics Database | NA | NA |
| progesterone | RAI14 | decreases expression | Comparative Toxicogenomics Database | -0.258702919 | 1.267330206 |
| calcitriol | RAI14 | decreases expression | Comparative Toxicogenomics Database | -0.258702919 | 1.267330206 |
| estradiol | RAI14 | decreases expression | Comparative Toxicogenomics Database | -0.258702919 | 1.267330206 |
| benzo[a]pyrene | RAI14 | decreases expression | Comparative Toxicogenomics Database | -0.258702919 | 1.267330206 |
| 7646-79-9 | RAI14 | decreases expression | Comparative Toxicogenomics Database | -0.258702919 | 1.267330206 |
| testosterone | RAI14 | decreases expression | Comparative Toxicogenomics Database | -0.258702919 | 1.267330206 |
| quercetin | RRM2 | decreases expression | Comparative Toxicogenomics Database | -0.243767014 | 1.737993167 |
| Silica | RRM2 | decreases expression | Comparative Toxicogenomics Database | -0.243767014 | 1.737993167 |
| Dasatinib | RRM2 | decreases expression | Comparative Toxicogenomics Database | -0.243767014 | 1.737993167 |
| progesterone | RRM2 | decreases expression | Comparative Toxicogenomics Database | -0.243767014 | 1.737993167 |
| gedunin | RRM2 | decreases expression | Comparative Toxicogenomics Database | -0.243767014 | 1.737993167 |
| 5-Fluorouracil | RRM2 | decreases expression | Comparative Toxicogenomics Database | -0.243767014 | 1.737993167 |
| bicalutamide | RRM2 | decreases expression | Comparative Toxicogenomics Database | -0.243767014 | 1.737993167 |
| Dronabinol | RRM2 | decreases expression | Comparative Toxicogenomics Database | -0.243767014 | 1.737993167 |
| calcitriol | RRM2 | decreases expression | Comparative Toxicogenomics Database | -0.243767014 | 1.737993167 |
| Demecolcine | RRM2 | decreases expression | Comparative Toxicogenomics Database | -0.243767014 | 1.737993167 |
| celastrol | RRM2 | decreases expression | Comparative Toxicogenomics Database | -0.243767014 | 1.737993167 |
| vincristine | RRM2 | decreases expression | Comparative Toxicogenomics Database | -0.243767014 | 1.737993167 |
| 67526-95-8 | RRM2 | decreases expression | Comparative Toxicogenomics Database | -0.243767014 | 1.737993167 |
| piroxicam | RRM2 | decreases expression | Comparative Toxicogenomics Database | -0.243767014 | 1.737993167 |
| genistein | RRM2 | decreases expression | Comparative Toxicogenomics Database | -0.243767014 | 1.737993167 |
| estradiol | RRM2 | decreases expression | Comparative Toxicogenomics Database | -0.243767014 | 1.737993167 |
| COPPER | RRM2 | decreases expression | Comparative Toxicogenomics Database | -0.243767014 | 1.737993167 |
| Pinosylvin | RRM2 | decreases expression | Comparative Toxicogenomics Database | -0.243767014 | 1.737993167 |
| resveratrol | RRM2 | decreases expression | Comparative Toxicogenomics Database | -0.243767014 | 1.737993167 |
| benzo[a]pyrene | RRM2 | decreases expression | Comparative Toxicogenomics Database | -0.243767014 | 1.737993167 |
| troglitazone | RRM2 | decreases expression | Comparative Toxicogenomics Database | -0.243767014 | 1.737993167 |
| LUCANTHONE | RRM2 | decreases expression | Comparative Toxicogenomics Database | -0.243767014 | 1.737993167 |
| GDC-0941 | RRM2 | decreases expression | Comparative Toxicogenomics Database | -0.243767014 | 1.737993167 |
| O,P'-DDT | RRM2 | decreases expression | Comparative Toxicogenomics Database | -0.243767014 | 1.737993167 |
| 7646-79-9 | RRM2 | decreases expression | Comparative Toxicogenomics Database | -0.243767014 | 1.737993167 |
| Tetradioxin | RRM2 | decreases expression | Comparative Toxicogenomics Database | -0.243767014 | 1.737993167 |
| testosterone | RRM2 | decreases expression | Comparative Toxicogenomics Database | -0.243767014 | 1.737993167 |
| estradiol | RWDD3 | decreases expression | Comparative Toxicogenomics Database | NA | NA |
| benzo[a]pyrene | RYR2 | decreases expression | Comparative Toxicogenomics Database | NA | NA |
| quercetin | SDPR | decreases expression | Comparative Toxicogenomics Database | NA | NA |
| quercetin | SELENBP1 | decreases expression | Comparative Toxicogenomics Database | NA | NA |
| estriol | SELENBP1 | decreases expression | Comparative Toxicogenomics Database | NA | NA |
| Glycidamide | SELENBP1 | decreases expression | Comparative Toxicogenomics Database | NA | NA |
| (17S)-17-hydroxy-13,17-dimethyl-1,2,6,7,8,14,15,16-octahydrocyclopenta[a]phenanthren-3-one | SELENBP1 | decreases expression | Comparative Toxicogenomics Database | NA | NA |
| genistein | SELENBP1 | decreases expression | Comparative Toxicogenomics Database | NA | NA |
| estradiol | SELENBP1 | decreases expression | Comparative Toxicogenomics Database | NA | NA |
| resveratrol | SELENBP1 | decreases expression | Comparative Toxicogenomics Database | NA | NA |
| benzo[a]pyrene | SELENBP1 | decreases expression | Comparative Toxicogenomics Database | NA | NA |
| estrone | SELENBP1 | decreases expression | Comparative Toxicogenomics Database | NA | NA |
| 635-65-4 | SELENBP1 | decreases expression | Comparative Toxicogenomics Database | NA | NA |
| methoxychlor | SELENBP1 | decreases expression | Comparative Toxicogenomics Database | NA | NA |
| COUMESTROL | SELENBP1 | decreases expression | Comparative Toxicogenomics Database | NA | NA |
| 7646-79-9 | SELENBP1 | decreases expression | Comparative Toxicogenomics Database | NA | NA |
| Tetradioxin | SELENBP1 | decreases expression | Comparative Toxicogenomics Database | NA | NA |
| diethylstilbestrol | SELENBP1 | decreases expression | Comparative Toxicogenomics Database | NA | NA |
| quercetin | SEMA3C | decreases expression | Comparative Toxicogenomics Database | -0.210040407 | 1.386851531 |
| calcitriol | SEMA3C | decreases expression | Comparative Toxicogenomics Database | -0.210040407 | 1.386851531 |
| 7646-79-9 | SEMA3C | decreases expression | Comparative Toxicogenomics Database | -0.210040407 | 1.386851531 |
| Tetradioxin | SEMA3C | decreases expression | Comparative Toxicogenomics Database | -0.210040407 | 1.386851531 |
| progesterone | SERTAD4 | decreases expression | Comparative Toxicogenomics Database | NA | NA |
| CADMIUM | SERTAD4 | decreases expression | Comparative Toxicogenomics Database | NA | NA |
| atrazine | SERTAD4 | decreases expression | Comparative Toxicogenomics Database | NA | NA |
| estradiol | SERTAD4 | decreases expression | Comparative Toxicogenomics Database | NA | NA |
| Adehl | SESN1 | decreases expression | Comparative Toxicogenomics Database | NA | NA |
| gedunin | SESN1 | decreases expression | Comparative Toxicogenomics Database | NA | NA |
| (17S)-17-hydroxy-13,17-dimethyl-1,2,6,7,8,14,15,16-octahydrocyclopenta[a]phenanthren-3-one | SESN1 | decreases expression | Comparative Toxicogenomics Database | NA | NA |
| Dronabinol | SESN1 | decreases expression | Comparative Toxicogenomics Database | NA | NA |
| adenine | SESN1 | decreases expression | Comparative Toxicogenomics Database | NA | NA |
| calcitriol | SESN1 | decreases expression | Comparative Toxicogenomics Database | NA | NA |
| formaldehyde | SESN1 | decreases expression | Comparative Toxicogenomics Database | NA | NA |
| TPEN | SESN1 | decreases expression | Comparative Toxicogenomics Database | NA | NA |
| celastrol | SESN1 | decreases expression | Comparative Toxicogenomics Database | NA | NA |
| Premarin | SESN1 | decreases expression | Comparative Toxicogenomics Database | NA | NA |
| 4-Hydroxytamoxifen | SESN1 | decreases expression | Comparative Toxicogenomics Database | NA | NA |
| piroxicam | SESN1 | decreases expression | Comparative Toxicogenomics Database | NA | NA |
| colchicine | SESN1 | decreases expression | Comparative Toxicogenomics Database | NA | NA |
| Myristicin | SESN1 | decreases expression | Comparative Toxicogenomics Database | NA | NA |
| 635-65-4 | SESN1 | decreases expression | Comparative Toxicogenomics Database | NA | NA |
| Tetradioxin | SESN1 | decreases expression | Comparative Toxicogenomics Database | NA | NA |
| testosterone | SESN1 | decreases expression | Comparative Toxicogenomics Database | NA | NA |
| theophylline | SH2D2A | decreases expression | Comparative Toxicogenomics Database | -0.283338745 | 2.467099567 |
| progesterone | SLAIN1 | decreases expression | Comparative Toxicogenomics Database | NA | NA |
| formaldehyde | SLAIN1 | decreases expression | Comparative Toxicogenomics Database | NA | NA |
| COPPER | SLAIN1 | decreases expression | Comparative Toxicogenomics Database | NA | NA |
| progesterone | SLC14A1 | decreases expression | Comparative Toxicogenomics Database | NA | NA |
| phenobarbital | SLC14A1 | decreases expression | Comparative Toxicogenomics Database | NA | NA |
| estradiol | SLC14A1 | decreases expression | Comparative Toxicogenomics Database | NA | NA |
| melphalan | SLC14A1 | decreases expression | Comparative Toxicogenomics Database | NA | NA |
| benzo[a]pyrene | SLC19A3 | decreases expression | Comparative Toxicogenomics Database | NA | NA |
| 3'-Azido-3'-deoxythymidine | SLC19A3 | decreases expression | Comparative Toxicogenomics Database | NA | NA |
| quercetin | SMIM3 | decreases expression | Comparative Toxicogenomics Database | -0.2458786 | 1.455555423 |
| Caspan | SMIM3 | decreases expression | Comparative Toxicogenomics Database | -0.2458786 | 1.455555423 |
| formaldehyde | SMIM3 | decreases expression | Comparative Toxicogenomics Database | -0.2458786 | 1.455555423 |
| estradiol | SMIM3 | decreases expression | Comparative Toxicogenomics Database | -0.2458786 | 1.455555423 |
| GDC-0941 | SMIM3 | decreases expression | Comparative Toxicogenomics Database | -0.2458786 | 1.455555423 |
| Tetradioxin | SMIM3 | decreases expression | Comparative Toxicogenomics Database | -0.2458786 | 1.455555423 |
| quercetin | SNTB1 | decreases expression | Comparative Toxicogenomics Database | NA | NA |
| melphalan | SNTB1 | decreases expression | Comparative Toxicogenomics Database | NA | NA |
| benzo[a]pyrene | SNTB1 | decreases expression | Comparative Toxicogenomics Database | NA | NA |
| quercetin | SORBS2 | decreases expression | Comparative Toxicogenomics Database | NA | NA |
| progesterone | SORBS2 | decreases expression | Comparative Toxicogenomics Database | NA | NA |
| acetaminophen | SORBS2 | decreases expression | Comparative Toxicogenomics Database | NA | NA |
| carmustine | SORBS2 | decreases expression | Comparative Toxicogenomics Database | NA | NA |
| estradiol | SORBS2 | decreases expression | Comparative Toxicogenomics Database | NA | NA |
| benzo[a]pyrene | SORBS2 | decreases expression | Comparative Toxicogenomics Database | NA | NA |
| 7646-79-9 | SORBS2 | decreases expression | Comparative Toxicogenomics Database | NA | NA |
| Columbamine | SOX17 | decreases expression | Comparative Toxicogenomics Database | NA | NA |
| acetaminophen | SOX17 | decreases expression | Comparative Toxicogenomics Database | NA | NA |
| N-NITROSODIETHYLAMINE | SOX17 | decreases expression | Comparative Toxicogenomics Database | NA | NA |
| resveratrol | SOX17 | decreases expression | Comparative Toxicogenomics Database | NA | NA |
| Enterolactone | SSBP2 | decreases expression | Comparative Toxicogenomics Database | NA | NA |
| formaldehyde | SSBP2 | decreases expression | Comparative Toxicogenomics Database | NA | NA |
| N-NITROSODIETHYLAMINE | SSBP2 | decreases expression | Comparative Toxicogenomics Database | NA | NA |
| COUMESTROL | SSBP2 | decreases expression | Comparative Toxicogenomics Database | NA | NA |
| progesterone | SSPN | decreases expression | Comparative Toxicogenomics Database | NA | NA |
| estradiol | SSPN | decreases expression | Comparative Toxicogenomics Database | NA | NA |
| cytarabine | ST6GALNAC3 | decreases expression | Comparative Toxicogenomics Database | NA | NA |
| Caspan | SULF1 | decreases expression | Comparative Toxicogenomics Database | -0.230481487 | 2.378923735 |
| 5-azacytidine | SULF1 | decreases expression | Comparative Toxicogenomics Database | -0.230481487 | 2.378923735 |
| SELENIUM | SULF1 | decreases expression | Comparative Toxicogenomics Database | -0.230481487 | 2.378923735 |
| estradiol | SULF1 | decreases expression | Comparative Toxicogenomics Database | -0.230481487 | 2.378923735 |
| Tetradioxin | SULF1 | decreases expression | Comparative Toxicogenomics Database | -0.230481487 | 2.378923735 |
| Bortezomib | SUSD4 | decreases expression | Comparative Toxicogenomics Database | NA | NA |
| estradiol | SUSD4 | decreases expression | Comparative Toxicogenomics Database | NA | NA |
| benzo[a]pyrene | SUSD4 | decreases expression | Comparative Toxicogenomics Database | NA | NA |
| Tetradioxin | SUSD4 | decreases expression | Comparative Toxicogenomics Database | NA | NA |
| Decitabine | SVEP1 | decreases expression | Comparative Toxicogenomics Database | NA | NA |
| cytarabine | SVEP1 | decreases expression | Comparative Toxicogenomics Database | NA | NA |
| 7646-79-9 | SVEP1 | decreases expression | Comparative Toxicogenomics Database | NA | NA |
| progesterone | SVIL | decreases expression | Comparative Toxicogenomics Database | NA | NA |
| acetaminophen | SVIL | decreases expression | Comparative Toxicogenomics Database | NA | NA |
| 4-Hydroxytamoxifen | SVIL | decreases expression | Comparative Toxicogenomics Database | NA | NA |
| estradiol | SVIL | decreases expression | Comparative Toxicogenomics Database | NA | NA |
| 7646-79-9 | SVIL | decreases expression | Comparative Toxicogenomics Database | NA | NA |
| Tetradioxin | SVIL | decreases expression | Comparative Toxicogenomics Database | NA | NA |
| calcitriol | SYNGR1 | decreases expression | Comparative Toxicogenomics Database | NA | NA |
| Vitinoin | SYNGR1 | decreases expression | Comparative Toxicogenomics Database | NA | NA |
| COPPER | SYNGR1 | decreases expression | Comparative Toxicogenomics Database | NA | NA |
| mifepristone | SYNGR1 | decreases expression | Comparative Toxicogenomics Database | NA | NA |
| testosterone | TBX15 | decreases expression | Comparative Toxicogenomics Database | NA | NA |
| quercetin | TET1 | decreases expression | Comparative Toxicogenomics Database | NA | NA |
| Caspan | TET1 | decreases expression | Comparative Toxicogenomics Database | NA | NA |
| melphalan | TET1 | decreases expression | Comparative Toxicogenomics Database | NA | NA |
| benzo[a]pyrene | TET1 | decreases expression | Comparative Toxicogenomics Database | NA | NA |
| Tetradioxin | TET1 | decreases expression | Comparative Toxicogenomics Database | NA | NA |
| diazepam | TGFBR3 | decreases expression | Comparative Toxicogenomics Database | NA | NA |
| vincristine | TGFBR3 | decreases expression | Comparative Toxicogenomics Database | NA | NA |
| SELENIUM | TGFBR3 | decreases expression | Comparative Toxicogenomics Database | NA | NA |
| benzo[a]pyrene | TGFBR3 | decreases expression | Comparative Toxicogenomics Database | NA | NA |
| cytarabine | TGFBR3 | decreases expression | Comparative Toxicogenomics Database | NA | NA |
| Tetradioxin | TGFBR3 | decreases expression | Comparative Toxicogenomics Database | NA | NA |
| atrazine | THBS4 | decreases expression | Comparative Toxicogenomics Database | NA | NA |
| melphalan | THSD4 | decreases expression | Comparative Toxicogenomics Database | NA | NA |
| 7646-79-9 | THSD4 | decreases expression | Comparative Toxicogenomics Database | NA | NA |
| theophylline | TMEM63A | decreases expression | Comparative Toxicogenomics Database | NA | NA |
| quercetin | TNS1 | decreases expression | Comparative Toxicogenomics Database | NA | NA |
| acetaminophen | TNS1 | decreases expression | Comparative Toxicogenomics Database | NA | NA |
| estradiol | TNS1 | decreases expression | Comparative Toxicogenomics Database | NA | NA |
| benzo[a]pyrene | TNS1 | decreases expression | Comparative Toxicogenomics Database | NA | NA |
| AM580 | TNS1 | decreases expression | Comparative Toxicogenomics Database | NA | NA |
| rosiglitazone | TNS1 | decreases expression | Comparative Toxicogenomics Database | NA | NA |
| monensin | TRPM2 | decreases expression | Comparative Toxicogenomics Database | -0.234690133 | 2.05762397 |
| Decitabine | TRPM2 | decreases expression | Comparative Toxicogenomics Database | -0.234690133 | 2.05762397 |
| quercetin | ZBED3 | decreases expression | Comparative Toxicogenomics Database | NA | NA |
| CROTONALDEHYDE | ZBED3 | decreases expression | Comparative Toxicogenomics Database | NA | NA |
| atrazine | ZBED3 | decreases expression | Comparative Toxicogenomics Database | NA | NA |
| acetaminophen | ZBTB16 | decreases expression | Comparative Toxicogenomics Database | NA | NA |
| Vitinoin | ZBTB16 | decreases expression | Comparative Toxicogenomics Database | NA | NA |
| resveratrol | ZBTB16 | decreases expression | Comparative Toxicogenomics Database | NA | NA |
| Tamibarotene | ZBTB16 | decreases expression | Comparative Toxicogenomics Database | NA | NA |
| Retinoic_acid | ZBTB47 | decreases expression | Comparative Toxicogenomics Database | NA | NA |
| METHYL_METHANESULFONATE | ZBTB47 | decreases expression | Comparative Toxicogenomics Database | NA | NA |
| quercetin | ZC2HC1C | decreases expression | Comparative Toxicogenomics Database | NA | NA |
| arsenite | ZFP28 | decreases expression | Comparative Toxicogenomics Database | NA | NA |
| acetaminophen | ZNF229 | decreases expression | Comparative Toxicogenomics Database | NA | NA |
| Cianidanol | ZNF229 | decreases expression | Comparative Toxicogenomics Database | NA | NA |
| atrazine | ZNF415 | decreases expression | Comparative Toxicogenomics Database | NA | NA |
| Decitabine | ZNF415 | decreases expression | Comparative Toxicogenomics Database | NA | NA |
| oxygen | ZNF415 | decreases expression | Comparative Toxicogenomics Database | NA | NA |
| formaldehyde | ZNF420 | decreases expression | Comparative Toxicogenomics Database | NA | NA |
| arsenite | ZNF471 | decreases expression | Comparative Toxicogenomics Database | NA | NA |
| Silica | ZNF568 | decreases expression | Comparative Toxicogenomics Database | NA | NA |
| benzo[a]pyrene | ZNF568 | decreases expression | Comparative Toxicogenomics Database | NA | NA |
| formaldehyde | ZNF569 | decreases expression | Comparative Toxicogenomics Database | NA | NA |
| arsenite | ZNF582 | decreases expression | Comparative Toxicogenomics Database | NA | NA |
| arsenite | ZNF583 | decreases expression | Comparative Toxicogenomics Database | NA | NA |
| arsenite | ZNF667 | decreases expression | Comparative Toxicogenomics Database | NA | NA |
| arsenite | ZNF677 | decreases expression | Comparative Toxicogenomics Database | NA | NA |
| arsenite | ZNF737 | decreases expression | Comparative Toxicogenomics Database | NA | NA |
| VALPROIC_ACID | ZNF853 | decreases expression | Comparative Toxicogenomics Database | NA | NA |
| VALPROIC_ACID | ZNF880 | decreases expression | Comparative Toxicogenomics Database | NA | NA |
| atrazine | ZSCAN16 | decreases expression | Comparative Toxicogenomics Database | NA | NA |
| arsenite | ZSCAN18 | decreases expression | Comparative Toxicogenomics Database | NA | NA |
