# Supplementary material for: Epigenomic dysregulation-mediated alterations of key biological pathways and tumor immune evasion are hallmarks of gingivo-buccal oral cancer
Source: Clin Epigenetics. 2019 Dec 3;11:178. doi: 10.1186/s13148-019-0782-2 (PMC6889354; doi:10.1186/s13148-019-0782-2)
Supplement: Supplementary file 3 — Additional file 3: Table S3. Significantly enriched GO terms in OSCC-GB patients based on 209 genes significantly differentially methylated in their promoter regions and related information. [file 13148_2019_782_MOESM3_ESM.docx]

**Supplementary Table S3: Significantly enriched GO terms in OSCC-GB patients based on 209 genes significantly differentially methylated in their promoter regions and related information.**

| **GO Term** | **No. Genes** | **% Associated Genes** | **Corrected P-Value** | **Associated Genes Found** |
| --- | --- | --- | --- | --- |
| response to prostaglandin E | 5 | 14.29 | 0.000 | *ADCY6, CCR7, P2RY6, PPARG, TGFBR3* |
| negative regulation of dendritic cell apoptotic process | 3 | 37.50 | 0.000 | *CCR7, CXCL12, LILRB1* |
| response to fatty acid | 8 | 7.34 | 0.001 | *ADCY6, CCR7, CD36, CHPT1, P2RY6, PDK4, PPARG, TGFBR3* |
| cellular response to fatty acid | 5 | 6.94 | 0.006 | *ADCY6, CHPT1, P2RY6, PDK4, PPARG* |
| regulation of leukocyte apoptotic process | 6 | 5.00 | 0.008 | *CCR7, CD274, CXCL12, LILRB1, PCYT1A, PIK3CD* |
| response to caffeine | 3 | 11.54 | 0.009 | *DNMT3B, PPARG, RYR2* |
| cellular response to prostaglandin E stimulus | 3 | 11.54 | 0.009 | *ADCY6, P2RY6, PPARG* |
| negative regulation of tumor necrosis factor superfamily cytokine production | 4 | 6.15 | 0.016 | *CD274, CD34, HAVCR2, LILRB1* |
| chemokine-mediated signaling pathway | 5 | 4.59 | 0.017 | *CCL24, CCR7, CCRL2, CXCL12, OSR1* |
| T cell costimulation | 4 | 5.41 | 0.019 | *CCR7, CD274, CD80, LCK* |
| regulation of macrophage derived foam cell differentiation | 3 | 6.98 | 0.023 | *CD36, PITX1, PPARG* |
| endodermal cell differentiation | 3 | 6.00 | 0.025 | *COL8A1, LAMA3, SOX17* |
| embryonic hindlimb morphogenesis | 3 | 6.52 | 0.025 | *OSR1, PITX1, ZBTB16* |
| monocyte differentiation | 3 | 6.12 | 0.025 | *GPR68, IL34, PPARG* |
| positive regulation of striated muscle tissue development | 4 | 4.55 | 0.026 | *AKAP6, CDON, SOX17, TGFBR3* |
| negative regulation of G0 to G1 transition | 3 | 5.17 | 0.030 | *MAGI2, PHC1, RRM2* |
| type I interferon signaling pathway | 4 | 4.00 | 0.030 | *BST2, IFITM1, MX2, OAS2* |
| regulation of filopodium assembly | 3 | 5.36 | 0.031 | *CCR7, PALM, TRPM2* |
| interleukin-1 beta secretion | 3 | 4.92 | 0.032 | *AIM2, CCR7, CD36* |
| regulation of interleukin-12 production | 3 | 4.55 | 0.037 | *CCR7, CD36, LILRB1* |
| regulation of interleukin-10 production | 3 | 4.29 | 0.041 | *CD274, CD34, LILRB1* |
| natural killer cell mediated cytotoxicity | 3 | 4.29 | 0.041 | *GZMB, HAVCR2, LILRB1* |
