# Supplementary material for: Epigenomic dysregulation-mediated alterations of key biological pathways and tumor immune evasion are hallmarks of gingivo-buccal oral cancer
Source: Clin Epigenetics. 2019 Dec 3;11:178. doi: 10.1186/s13148-019-0782-2 (PMC6889354; doi:10.1186/s13148-019-0782-2)
Supplement: Supplementary file 4 — Additional file 4: Figure S1. Scatter diagram showing the relationship of average β values of the DMPs found in the present study with those in the TCGA study, irrespective of whether these probes were also significantly differentially methylated in the TCGA study. Each point on the scatter diagram indicates for a DMP of the present study the average β value over the 44 OSCC-GB patients included in the validation subset and, for the TCGA study, averaged over the 31 OSCC-GB patients. Figure S2. Integrated unsupervised hierarchical clustering and heatmap using quantitative methylation level of 3741 DMPs mapped to gene promoter from (A) 44 OSCC-GB patients included in this study and (B) 31 OSCC-GB patients included in TCGA study. Corresponding clusters obtained from two sets of patients showed similar methylation pattern and phenotypic features. [White box in top panels of (A) and (B) indicates unavailability of the respective clinical information]. [file 13148_2019_782_MOESM4_ESM.docx]

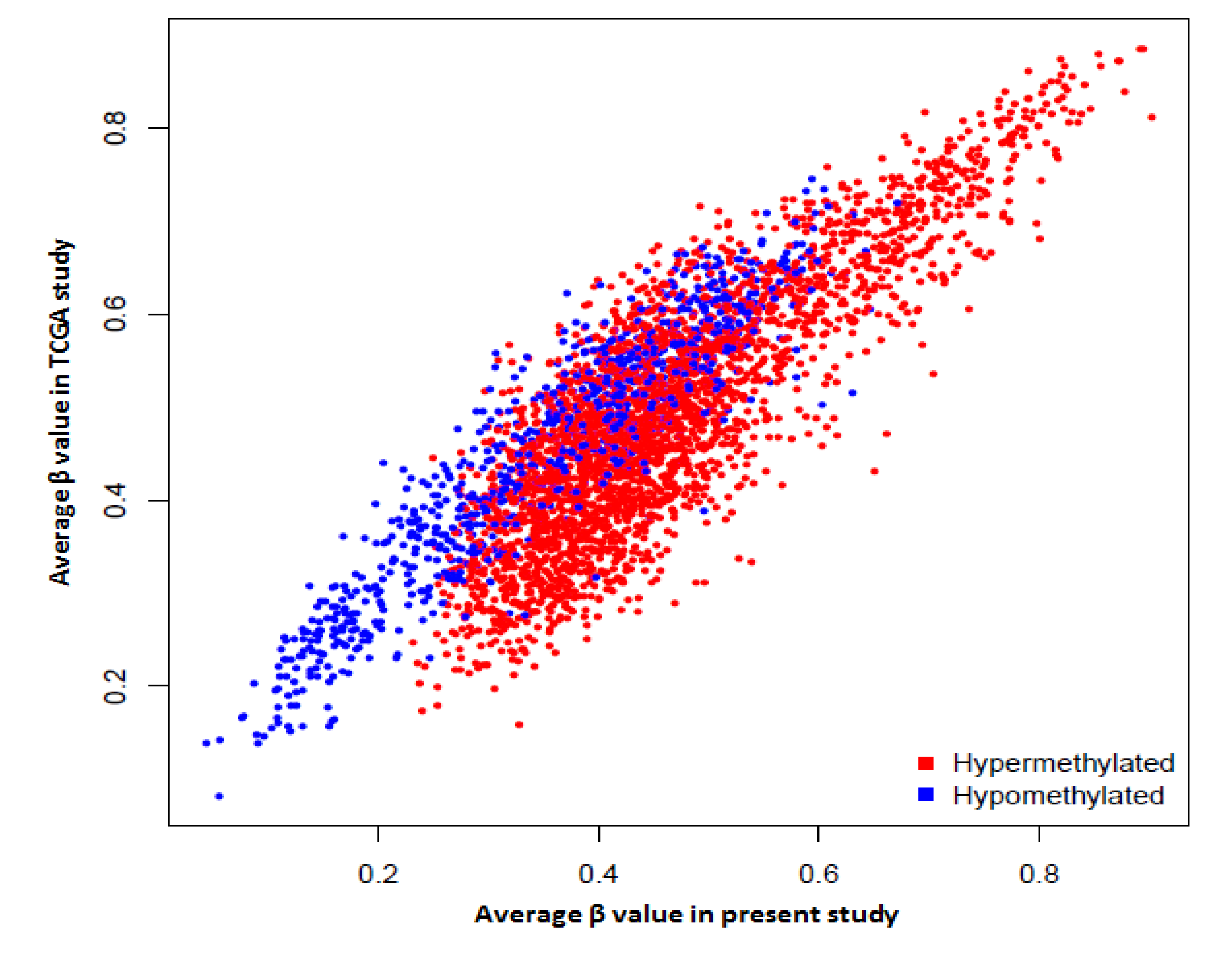


**Figure S1:** Scatter diagram showing the relationship of average β values of the DMPs found in the present study with those in the TCGA study, irrespective of whether these probes were also significantly differentially methylated in the TCGA study. Each point on the scatter diagram indicates for a DMP of the present study the average β value over the 44 OSCC-GB patients included in the validation subset and, for the TCGA study, averaged over the 31 OSCC-GB patients.

**
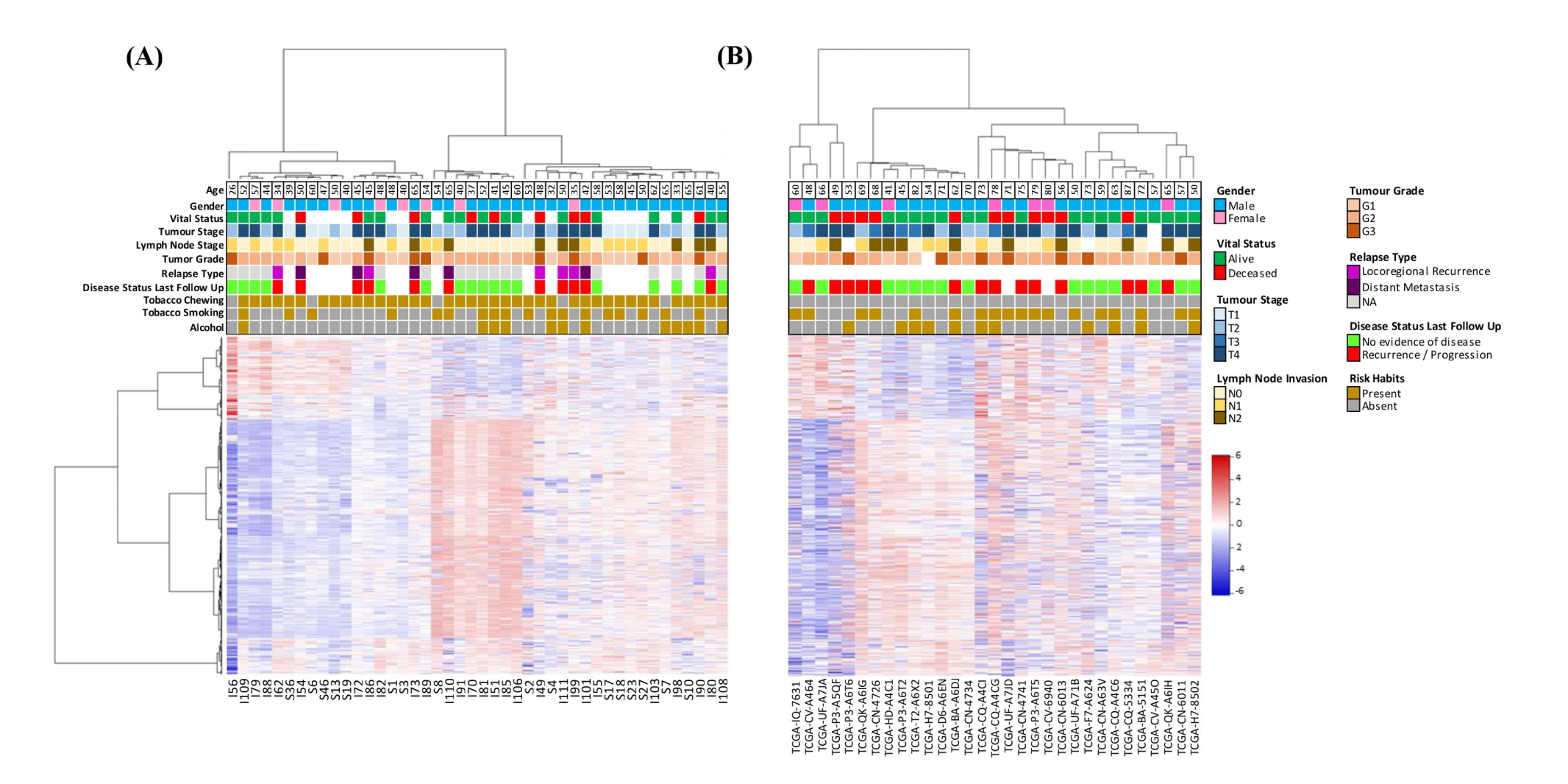
**

**Figure S2:** Integrated unsupervised hierarchical clustering and heatmap using quantitative methylation level of 3741 DMPs mapped to gene promoter from (A) 44 OSCC-GB patients included in this study and (B) 31 OSCC-GB patients included in TCGA study. Corresponding clusters obtained from two sets of patients showed similar methylation pattern and phenotypic features. [White box in top panels of (A) and (B) indicates unavailability of the respective clinical information]
